# Discovering viral genomes in human metagenomic data by predicting unknown protein families

**DOI:** 10.1038/s41598-017-18341-7

**Published:** 2018-01-08

**Authors:** Mauricio Barrientos-Somarribas, David N. Messina, Christian Pou, Fredrik Lysholm, Annelie Bjerkner, Tobias Allander, Björn Andersson, Erik L. L. Sonnhammer

**Affiliations:** 1 Department of Cell and Molecular Biology, Science for Life Laboratory, Karolinska Institutet, PO Box 285, SE-171 77 Stockholm, Sweden; 2 Stockholm Bioinformatics Center, Department of Biochemistry and Biophysics, Stockholm University, Science for Life Laboratory, Box 1031, SE-171 21 Solna, Sweden; 3 IFM Bioinformatics and Swedish e-Science Research Centre (SeRC), Linköping University, SE-581 83 Linköping, Sweden; 4 Karolinska Institutet, Department of Microbiology, Tumor- and Cell Biology, Laboratory for Clinical Microbiology, Karolinska University Hospital, SE-171 76 Stockholm, Sweden

## Abstract

Massive amounts of metagenomics data are currently being produced, and in all such projects a sizeable fraction of the resulting data shows no or little homology to known sequences. It is likely that this fraction contains novel viruses, but identification is challenging since they frequently lack homology to known viruses. To overcome this problem, we developed a strategy to detect ORFan protein families in shotgun metagenomics data, using similarity-based clustering and a set of filters to extract bona fide protein families. We applied this method to 17 virus-enriched libraries originating from human nasopharyngeal aspirates, serum, feces, and cerebrospinal fluid samples. This resulted in 32 predicted putative novel gene families. Some families showed detectable homology to sequences in metagenomics datasets and protein databases after reannotation. Notably, one predicted family matches an ORF from the highly variable Torque Teno virus (TTV). Furthermore, follow-up from a predicted ORFan resulted in the complete reconstruction of a novel circular genome. Its organisation suggests that it most likely corresponds to a novel bacteriophage in the microviridae family, hence it was named bacteriophage HFM.

## Introduction

Characterization of the human virome is crucial for our understanding of the role of the microbiome in health and disease. The shift from culture-based methods to metagenomics in recent years, combined with the development of virus particle enrichment protocols, has made it possible to efficiently study the entire flora of human viruses and bacteriophages associated with the human microbiome. These methods have led to the discovery of numerous human viruses^1^ and human-resident bacteriophages^2^, and have made it possible to characterize the virus content of entire collections of clinical samples^3,4^.

Traditional characterization of virome datasets has largely relied on homology-based approaches^5–8^. These methods can accurately identify sequences from characterized virus families and distant relatives, but they are unable to annotate viral sequences that have little or no sequence similarity to known viruses. Therefore, a substantial fraction of microbiome datasets cannot be classified, despite the recent rapid increase of sequence information in public databases. For instance, the recent identification of the crAssphage^9^ illustrates how sequence homology-based methods have failed to recognize a bacteriophage genome constituting 1.7% of all available fecal metagenomic data.

In viral-enriched metagenomics datasets, we expect that a fraction of these unclassifiable sequences originate from protein coding segments from unknown viruses and other kinds of “biological dark matter”^9^. Consequently, the detection of coding sequences with no homologs, or ORFans^10^, in such datasets can be a first step towards the discovery of novel viral species, since novel protein sequences can be used as anchors for the characterization of entire viral genomes.

However, the discovery of novel ORFans in the “dark matter” fraction of metagenomic datasets is challenging. Due to technical limitations, such as insufficient coverage, short read lengths and sequencing errors, only partial ORFs can be reconstructed and the origin and function of incomplete genes are difficult to predict^11^.

Most current metagenomics gene finders rely on hidden Markov Models, which model statistical differences between coding and non-coding nucleotide frequencies and other features to estimate the probability that an open reading frame encodes a protein^12–16^. However, novel protein-coding genes can also be detected by the alignment of unknown related protein sequences, combined with the use of K_A_/K_S_ ratios to detect sequences that are under selection pressure. The advantage of this method is that it searches for conserved signals directly within the dataset using a fixed statistical model, and thus do not depend on a previous training procedure. Ab-initio K_A_/K_S_ methods are best applied when analysing together diverse datasets, since it leverages protein diversity to make accurate predictions. For instance, this strategy was used by the Global Oceanic Survey (GOS), which identified ~1700 putative novel ORFan protein families^17^.

In the present study, we applied a K_A_/K_S_ – based strategy to detect ORFans in the “dark matter” fraction of virome datasets from human patients. The analysis resulted in the identification of 32 putative novel ORFan protein families with strong support, two of which could be assigned a viral origin. Additionally, PCR-based cloning and sequencing starting from one of the novel families resulted in the complete genome sequence of a previously unknown 5,752 bp circular genome present in the human digestive tract. Additional analysis suggests that this is the genome of a novel bacteriophage.

## Results

### Sample sequencing and preprocessing

We analysed shotgun metagenomics datasets produced explicitly for viral discovery. Each library consists of DNA or RNA extracted from a pool of samples from patients with common clinical manifestations. This pooling strategy has proved to be a cost-effective strategy to identify novel viruses, since each sample is not sequenced individually, while preserving sensitivity^18^. Samples in this study originate from nasopharyngeal aspirates, serum, feces and cerebrospinal fluid (csf). Briefly, pools of patient material were subjected to filtration and nuclease treatment to enrich the viral fraction while depleting host and bacterial nucleic acid^18^. DNA and RNA libraries were prepared separately from each pool, yielding a total of 17 sequenced libraries. Table 1 summarizes the resulting sequencing data, and more detailed information about each library is presented in Supplementary Table S1.

**Table 1:** Summary of the sequenced viral-enriched libraries.

Sample type	DNA Libraries	RNA Libraries	454 Platform	Total Reads
Feces	1	1	Titanium	1 459 816
Serum	4	3	GS FLX & Titanium	1 095 915
Nasopharyngeal Swabs	2	2	GS & GS FLX	703 790
Nasopharyngeal & Throat Swabs	1	1	Titanium	432 919
Cerebrospinal Fluid	1	1	Titanium	209 748
			**Total Reads**	3 902 188

The libraries analysed were prepared by pooling patient specimens of 5 different sample types. At least one DNA and one RNA library for each sample type, but in some cases more libraries were sequenced. The libraries were sequenced during the period of 2008 through 2012, during which the 454-pyrosequencing platform evolved, which is reflected in the different total number of reads per library.

**Figure 1:**
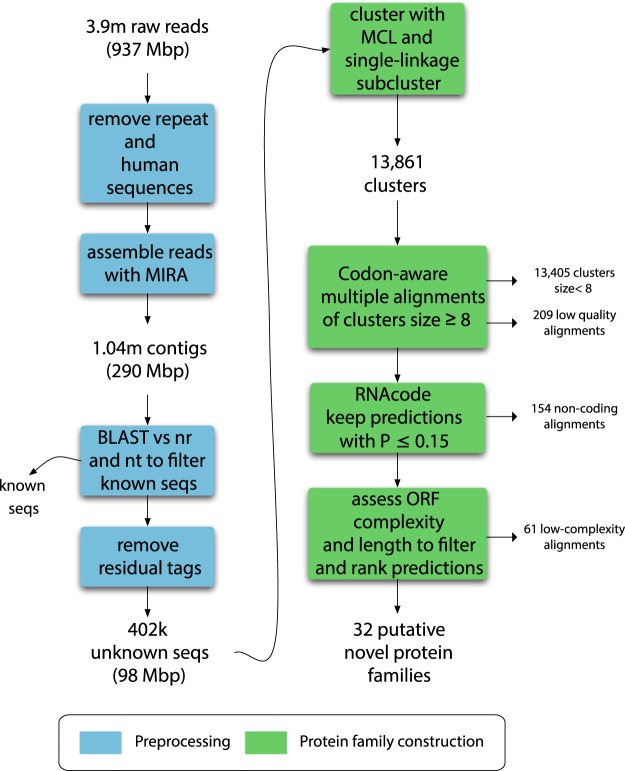
Flowchart of the ORFan protein family prediction pipeline. The diagram starts with the raw set of reads from the libraries described in Table 1. Squares in blue describe the preprocessing steps performed to obtain a data set consisting of unannotated sequences. The unannotated sequences were subsequently processed through our prediction pipeline (in green) resulting in 32 predicted protein families.

All datasets were previously analysed with our previously published pipeline^7^, but a sizable fraction of remained unannotated. Therefore, we decided to combine the libraries to mine for putative novel protein families in the uncharacterized fraction. The outline of our pipeline is described in Fig. 1. The pre-processing steps are aimed at producing a non-redundant set of unclassifiable sequences for further investigation, and are analogous to homology-based analysis pipelines^7,8^. These include trimming adapters, removal of low complexity sequences and host contamination, extending reads via sequence assembly, and removing sequences with significant matches to NCBI nt and nr, since our aim was to investigate unannotated sequences. The quality and human sequence filtering resulted in the removal of 37% of the initial reads. The subsequent assembly of the remaining 2.44 million reads produced 1.04 million contigs, increasing the average sequence length by 16%, to 278.6 nt, and significantly reducing redundancy. Still, 94% of the sequences after assembly were singletons, which is indicative of the complexity of the data set. Both contigs and singletons were included in our analyses. Finally, a conservative screening against NCBI nt and nr to discard all sequences that were classifiable by homology methods resulted in a final data set consisting of 402,288 sequences. We hypothesized that these sequences could be a rich source of putative novel protein families.

### Protein family prediction

To predict protein-coding sequences in the unannotated fraction, we based our strategy on RNAcode, a gene predictor that evaluates multiple sequence alignments using a K_A_/K_S_-based evolutionary model to distinguish between coding and non-coding sequences. In short (see Methods and supplementary materials for additional results and description), we generated candidate protein families by building high-quality multiple sequence alignments from clusters of similar but divergent sequences. These alignments were subsequently evaluated with RNAcode for coding potential, and a calibration procedure allowed us to select a suitable threshold for using this method with short NGS reads. Finally, the adjusted RNAcode prediction was combined with other measures such as predicted ORF length and sequence complexity to select and rank high-quality protein family candidates. The steps are summarized by the green boxes in Fig. 1.

**Figure 2:**
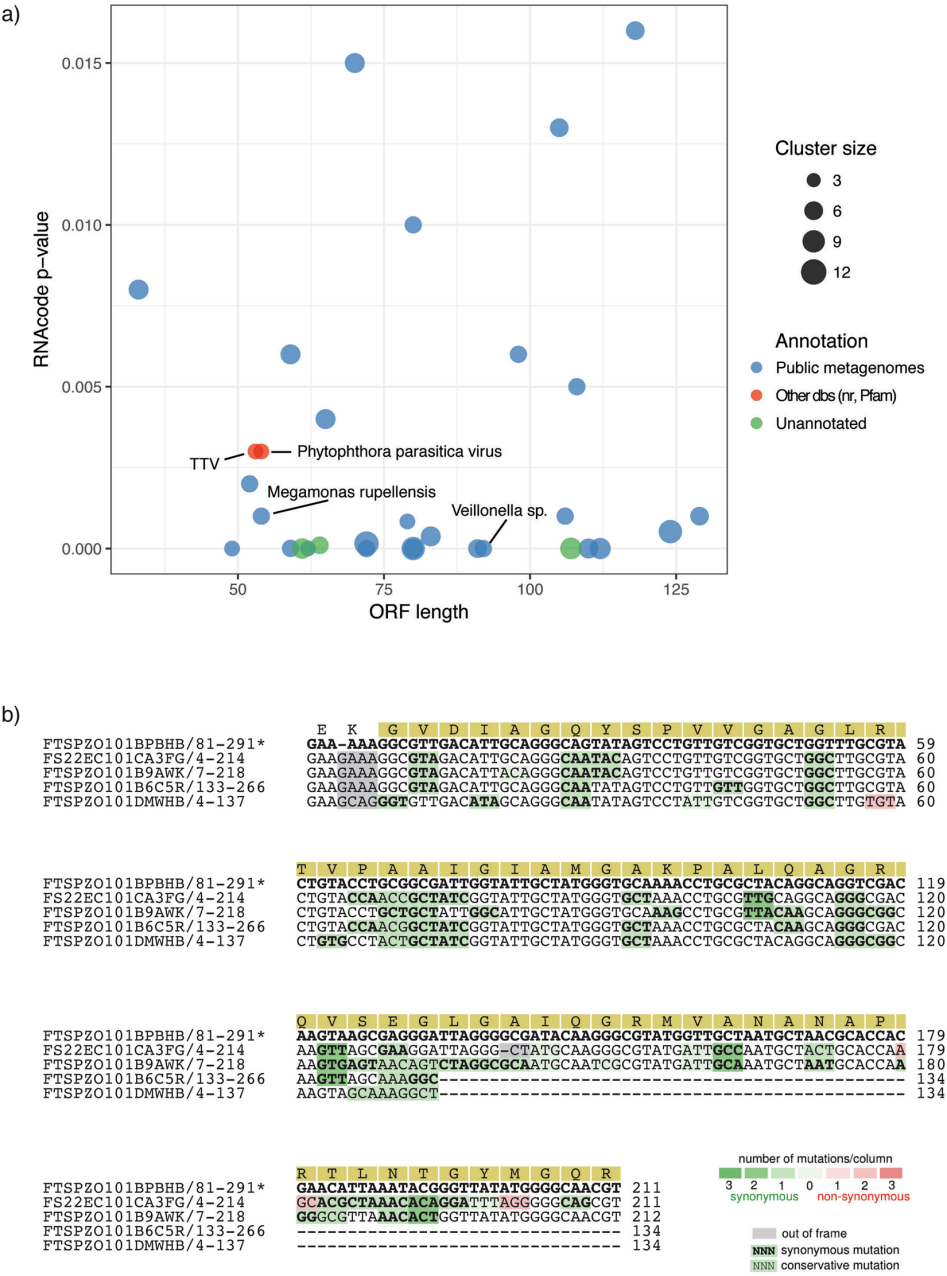
(**a**) Summary of the resulting 32 high confidence families. The scatterplot summarizes the basic statistics of the predicted proteins. The X and Y axis encode for ORF length and RNAcode p-value respectively, while the size of the dots are scaled by number of sequences in the alignment. Protein families are colored based on their hits to the different databases. (**b**) Example of RNAcode output for predicted ORFan family 457. The multiple alignment for cluster 457 is shown with the RNAcode-predicted peptide sequence on the top and the high-scoring segment highlighted in yellow. Codons colored in green indicate the presence of synonymous mutations, suggesting that selective pressures act on those sites to preserve the amino acid. In contrast, pink or red codons indicate non-synonymous mutations which do not preserve the amino acid encoding.

We applied this method to the 402 288 unannotated sequences from our data sets, and this resulted in a shortlist set of 32 predicted protein families. Figure 2a summarizes the main attributes of the predicted families, including ORF length, and predicted RNA-code p-value and sequences in the alignment. Detailed results of the protein family building procedure are available in Supplementary Figure 1.

### Annotation of predicted ORFan protein families

We screened the 32 high-confidence ORFan families against public metagenomics databases in search for homologous proteins detected in other microbiomes: the NCBI environmental databases (env_nt and env_nr) and the MetaHIT integrated gene catalogue^19^. Most of the families (27/32) had a hit to at least one metagenomics database. Summary of the hits are available in Table 2 and Fig. 2a, and more detailed per-cluster hit information is available in in Supplementary Table S2.

**Table 2:** Summary of how many of the 32 predicted novel ORFan protein families have hits to various microbiome or other databases.

Protein family origin	# families	Microbiome Databases			NCBI nr	No Hits
		MetaHIT	NCBI env	HMRGD		
Fecal	23	23	human gut (22); marine & human gut (1)	0	2	0
Fecal & CSF	1	1	gut (1)	0	0	0
Serum	1	0	—	0	1	0
Serum & CSF	5	0	wastewater (1)	0	1	3
Serum, CSF & Mucus	2	1	wastewater (1)	0	1	0

The families are grouped by the source of their samples.

Interestingly, all 24 families that originated from fecal samples matched nucleotide or peptide sequences in the MetaHIT integrated gene catalogue (Supplementary Table S3). Additionally, these families also showed similarity to sequences from other gut metagenomes in the env nt database, which is expected. In contrast, all but one of the clusters comprised of sequences originating from non-fecal libraries (csf, nasopharyngeal aspirates, and/or serum) did not show hits to gut microbiome-derived proteins. We did not find hits to other human microbiome datasets for the non-fecal families, but three clusters (457, 540 and 1217) matched sequences in wastewater and marine metagenomes (Table S4).

We also re-screened all protein families against updated versions of NCBI nr and nt databases (March 2016) and the Human Microbiome Project reference genomes data (HMRGD). This was necessary due to the high probability that some of these proteins were included in data sets produced since the original screening (performed in 2011). Five candidate protein families were found to match annotated proteins from the NCBI nr database (Table 3). No sequences matched NCBI nt, suggesting that the candidate protein families may not originate from well characterized organisms. Searches against the Rfam database to exclude the possibility that the clusters encode RNA genes also yielded no significant matches (E < 0.01).

**Table 3:** Protein family hits to described proteins.

Family	DB	Tool	Best Hit (protein)	Best hit (species)	Curated Annotation
1217	nr	blastp & hmmsearch	unknown	Veillonella sp. CAG:933	Bacterial protein
532	nr	hmmsearch	hypothetical protein	M. rupellensis	Bacterial protein
565b	nr	blastp	hypothetical protein H257_12751	A. astaci	Putative replication protein, viral or bacterial
	nr	hmmsearch	putative replication protein	Phytophthora parasitica virus	
956b	nr	blastp & hmmsearch	hypothetical protein	C. trachomatis	Torque Teno virus ORF

Four of the 32 ORFan protein families match proteins in the NR database. The table describes for each family: (1) the database of the hit, (2) the tool used to detect the similarity, (3) the description of the highest scoring hit, (4) the annotated species for the highest scoring hit, and (5) our manually curated annotation for the protein based on all the significant hits for the protein family. Manual annotation was required since the best hit for a sequence does not always correspond to the most plausible annotation, due to wrong metadata or to the discovery of a distant relative of a protein conserved in many different organisms.

Two putative viral protein families were detected: 565b and 956b. Cluster 565b has multiple hits to replication proteins from several viruses, with the highest similarity to a fungal virus domain from the Phyphtopora parasitica virus, although it also matches hypothetical proteins from fungal species. Cluster 956b has a significant hit to a Torque teno virus (TTV) ORF, consistent with the fact that the sequences came from serum libraries, and that TTV is present in blood. Also, while the match to Torque teno virus is highly significant, only ~50% of the residues are identical, suggesting that it may represent a new member of this highly variable virus family. Even though these families cannot strictly be considered ORFans after these findings, these hits confirm that our approach can predict protein-coding genes from viruses.

Beside the viral protein hits, only two other families,1217 and 532, matched the protein databases. In both cases, the hits corresponded to hypothetical proteins from bacteria (Table 3). Three families did not match any of the databases. All of these were composed of sequences from the serum and csf libraries.

However, we also observed some anomalies in our annotation. Cluster 179b, from fecal origin, has only a nucleotide level hit to the MetaHIT integrated gene catalogue but no protein level hit associated with it (Supplementary Table S2). Further investigation revealed that the match occurred in the 5′ UTR region of the gene, explaining the lack of a peptide hit and consequently the family could be a false positive. The p-value threshold optimization showed that while the 0.15 cut-off showed the best separation of classes, it was possible to find non-coding sequences below the threshold, which explains the occurrence of false positives. We speculate that since 5′ UTRs are often conserved, RNAcode could have overestimated the coding potential. Cluster 179b scored poorly (ranked 31 out of 32 protein families using the composite score.

### Discovery of a novel virus-like genome

Since the origin of most of the predicted protein families remained unknown after reannotation, the families remained good candidates for novel viral discovery. Thus, we designed outward primers from selected protein families to detect small circular genomes using PCR. This procedure resulted in the detection of a 5.8 kb fragment derived from primers designed from cluster 179a.

**Figure 3:**
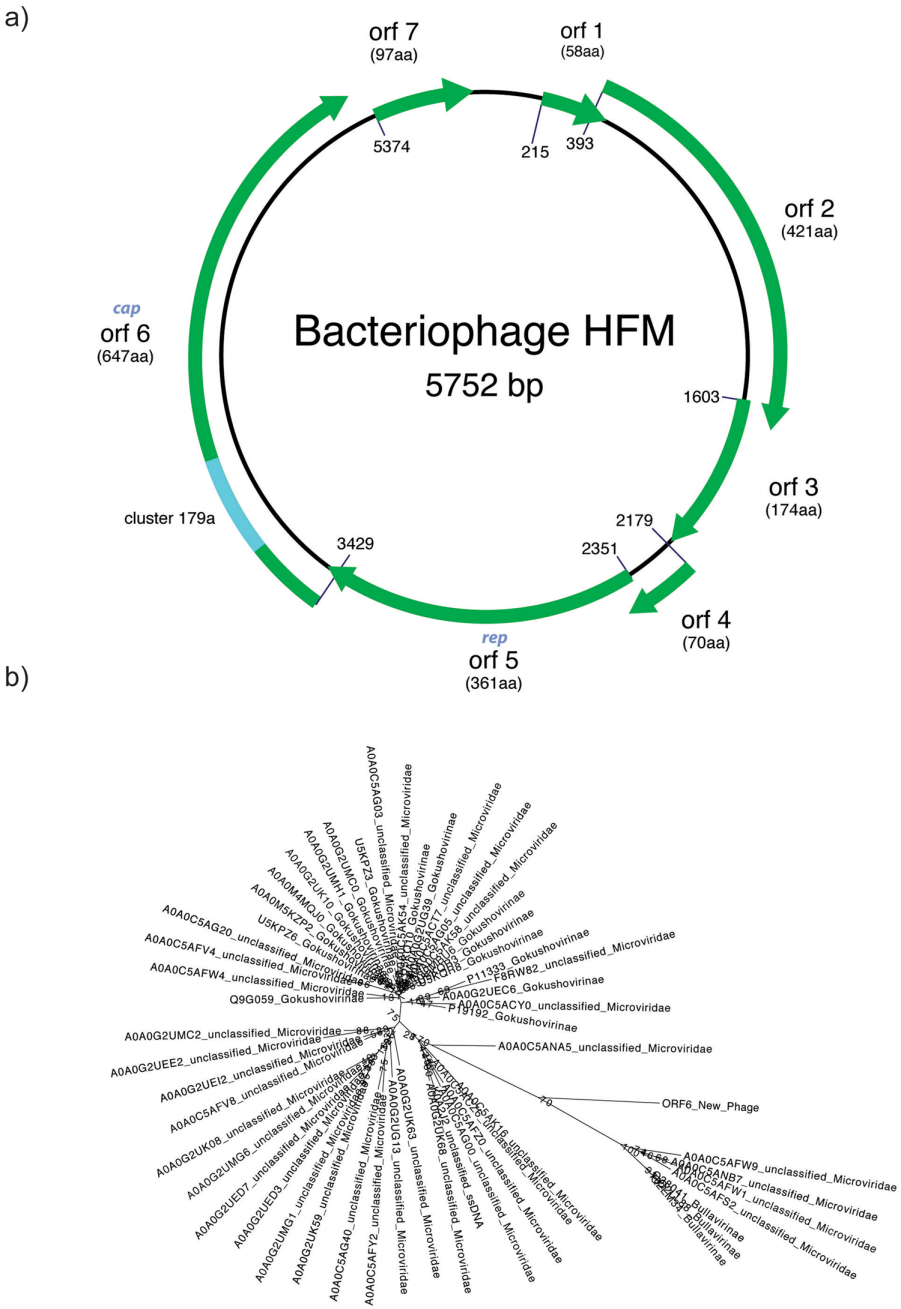
(**a**) Diagram of the bacteriophage HFM genome. This circular fragment was amplified from fecal samples using primers designed based on cluster 179a. The genome contains 7 candidate ORFs, all of which are located in the same strand and cover ~93% of the genome. Annotation suggests viral provenance due to the presence viral-like protein motifs such as a phage capsid motif (cap) and a replication protein (rep). The protein family (cluster 179a) from which the primers were designed is highlighted in light blue in ORF 6. (**b**) Phylogenetic tree showing the position of bacteriophage HFM in relation to 54 clearly annotated Microviridae genomes from the public databases. Due to lack of homology, it was impossible to include more distantly related sequences. It is a maximum likelihood tree, calculated using RAxML with 1000 bootstraps.

We sequenced this fragment using both Illumina and Sanger sequencing, and the reads were assembled into a 5,752 bp circular contig (European Nucleotide Archive accession number ERZ376945) (Fig. 3a). This genome has not been previously characterized and, aside from one protein, does not show similarity to any annotated genomes. However, it does show high (~90%) similarity to a few uncharacterized contigs from assembled gut metagenomes in the NCBI environmental nucleotide database, but there are important differences (Supplementary Figure S3). None of the contigs in the public databases are marked as circular, and they have discrepancies at their 5′ and 3′ ends compared to with our circular assembly. The differences could be attributed either to misassemblies or to recombination or integration events.

Annotation of the circular genome led to the detection of seven putative open reading frames that cover more than 93% of the genome. All the ORFs were oriented in the same direction and exhibited high degrees of homology (id > 70%) to proteins in the MetaHIT integrated gene catalogue (Supplementary Table S5; Supplementary material). Furthermore, three ORFs have significant hits to other proteins: ORF 3 shows similarity to hypothetical proteins, ORF 5 matches replication proteins from different organisms, and most interestingly, ORF 6 shows some similarity to the phage capsid F protein family in the Pfam database. We also observed two small ORFs (<50aa) in the gap between ORF 7 and ORF 1 with similarities to MetaHIT genes, but these were excluded from the final annotation due to their size.

Although our pipeline can detect new genomes without homology to known proteins, the fortuitous presence of the ORF 6 phage capsid F protein motif allows for classification of this genome. This suggests that it belongs to a single stranded DNA phage, further supported by the fact that all detected ORFs are on the same strand. The overall genome structure and ORF map is similar to that of the family Microviridae^20^, a family of circular ssDNA bacteriophages, with regard to the distribution and orientation of the ORFs. Phylogenetic analysis of the ORF 6 fragment was performed using the sequences from the capsid F protein family with proper taxonomical annotation. However, since all known putative homologues belong to the family Microviridae, this suggests that bacteriophage HFM is related to this viral family. Of the two subfamilies Bullavirinae and Gokushovirinae, our phage appears to be closer to Bullavirinae, although not closely related (Fig. 3b). Thus, it appears likely that the organism is a bacteriophage, which we have tentatively named bacteriophage HFM (Human Fecal Microbiome). PHACTS^21^ annotation of the genome predicted a gram-negative bacterium as host, but provided no prediction regarding the possible lifestyle of the phage (Supplementary Figure S4).

A specific PCR assay was designed and used to detect bacteriophage HFM in the individual fecal samples included in the original library. Only one of the ten available samples tested positive in the first and second PCR (Supplementary Figure S6). PCR products from both reactions were purified and capillary sequenced to verify the specificity of the amplification. The resulting sequence aligned perfectly with the bacteriophage HFM genome.

### Virus detection using other pipelines

**Figure 4:**
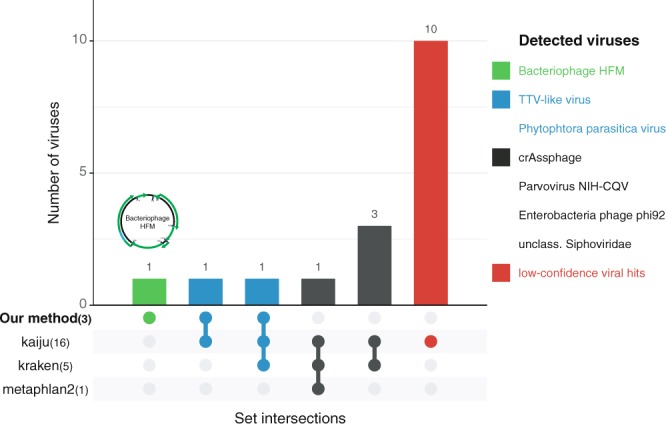
Detection of known viruses using homology-based methods. Our method was used to detect unknown viruses, while Kaiju, Kraken and Metaphlan2 were used to detect known viruses. UpSet^49^ plot showing the overlaps between the viral species detected by each tool and our method. The number of species detected by each tool is stated between parentheses next to the tool name, and the bar reflects the number of viruses detected by a specific combination of tools. The Phytophtora parasitica virus was detected as a novel family by our method as it was not a known virus at that time, and the family matching TTV only had very weak similarity.

Our approach was designed to discover protein families that lack homology to known proteins. To test whether these could have been detected using other virus finding tools, we analyzed our “dark matter” dataset with three standard tools for taxonomic annotation: Kraken^22^, Metaphlan2^23^, and Kaiju^24^ (Fig. 4). Note however that these tools were run four years after this dark matter dataset was defined, which gives them the additional knowledge of all the viruses that were described after the initial analysis. Of our 32 predicted protein families, only two were detected by the other tools: TTV-like (family 956b) and Phytophtora parasitica virus (family 565b), detected by Kaiju and Kraken. However, none of the reads labelled as viral by the three tools align to the genome of bacteriophage HFM, demonstrating that only our approach was able to detect this novel phage. In addition, at the time our method was run, the two other viruses had little or no homologs in the databases.

The other tools further reported 14 viral hits not detected by our method, of which four were supported by at least two of the tools, see Fig. 4. These include known viruses, such as crAssphage, Parvovirus NIH-CQV^25^, Enterobacteria phage phi92, and an unclassified Siphoviridae. The other ten hits were low confidence. The likely reason that these viruses were not detected using our method is that we applied our method with stringent cut-offs and only report the best supported families. This shows that our method is complementary to homology-based methods.

## Discussion

The field of microbiome research has advanced substantially in recent years, and its role in the characterization of the human virome has been fundamental. However, many aspects of the analysis of complex microbiome datasets remain a challenge, including the identification and characterization of new unknown organisms and genes. Recent studies have shown the power of a large sample sizes to discover previously unknown viruses^26,27^. Nevertheless, in settings where such large sample numbers are unavailable (e.g. rare diseases), our pooling strategy coupled with viral enrichment has been shown to reveal a sizable fraction of unknowns, but novel strategies to leverage these datasets are needed.

The identification of ORFan genes in metagenomics datasets can be a starting point for novel virus discovery, but two main challenges must be addressed. First, the characteristics of metagenomics sequence data can confound ab-initio gene prediction, since these datasets are composed of short sequence fragments burdened with sequencing errors. Second, the large amount of contaminating host and bacterial sequences often limits the coverage of the target genomes. These challenges can be overcome by combining diverse datasets and applying a calibrated method for short reads.

In this article, we applied an RNAcode-based strategy to predict novel ORFans in viral-enriched short-read metagenomic datasets. Using this approach, we identified 32 putative ORFan protein families from 17 DNA and RNA libraries from feces, serum, nasopharyngeal aspirates and cerebrospinal fluid. Reannotation of the sequences with updated databases suggest that our predicted families are likely to represent real ORFan gene families, since most of the families have high similarity to contigs from metagenomic sample assemblies but no significant hits to characterized proteins. Moreover, three predicted families did not match sequences in any of the databases. These are ORFan proteins by definition, but further validation is necessary to rule out false positives.

Based on the predicted protein families, we were able to amplify, characterize and validate the presence a circular DNA fragment of 5.8 kb, which we originally conjectured to be a small circular genome or a plasmid. The presence of phage capsid and replication protein motifs in the annotated ORFs, in conjunction with the fact that the fragment was isolated from fecal samples, suggest that the fragment is the genome of a bacteriophage, preliminarily named bacteriophage HFM. Further experiments are needed to investigate the nature and ubiquity of this putative bacteriophage, as recent studies provide increasing evidence that the bacteriophage portion of the microbiome may profoundly affect our health^28^.

The results of this study highlight the importance of developing methods for viral discovery that do not rely on homology to complement homology-based approaches. Although the analysis of the “dark matter” dataset with Kraken, Kaiju and Metaphlan2 yielded hits to 16 viral species, none of them predicted sequences belonging to bacteriophage HFM as viral. Besides bacteriophage HFM, our method also detected the presence of viral proteins from a TTV-like virus and Phytophtora parasitica virus, which also serve as additional evidence to support the hits to these viruses in Kaiju and Kraken.

Our approach requires the construction of high-quality multiple sequence alignments and we tested different strategies to guarantee a high-quality alignment input for RNAcode. This requirement limited the amount of putative families we could process. It is possible that crAssphage and the parvovirus NIH-CQV were not detected due to the stringent criteria used. Further work towards ensuring high quality input for RNAcode could thus increase the sensitivity and applicability of the proposed strategy.

Despite our positive results, there are several factors that could potentially influence the reliability and wider applicability of our approach. These include the choice of clustering algorithm. MCL performed the best in our pilot experiments, but the high memory requirements of MCL may make it unsuitable for larger datasets. Also, the addition of a single-linkage subclustering step at 95% similarity threshold aimed at reducing redundancy and improving multiple sequence alignment construction, but at the same time it could have artificially reduced the real diversity present in the dataset. Another limitation, inherent to RNAcode, is that one sequence must be selected in the multiple alignment as a reference for the analysis. Wherever possible, we reordered our multiple alignments such that the sequence designated as reference contained the most common codon variants, thereby ensuring that RNAcode would see the least amount of conserved changes and score the coding potential conservatively. It is likely that the effect of different choices in reference sequence is low in most cases, but highly dependent on the nature of the alignment.

The main advantage of our RNAcode-based strategy is that its efficacy does not depend on having a suitably trained model appropriate for the type of sample. This means that reliable gene prediction is possible for novel kinds of datasets, as long as there is sufficient conserved signal for the available ORFans. In this work, we showcased the potential of this strategy by analysing a combination of human virome datasets, which resulted in the characterization of a novel bacteriophage and a set of ORFan proteins that can be used as anchors for further exploration of potential novel viruses and bacteriophages.

Despite our contribution to *ab initio* protein family detection, prediction of novel viral ORFan genes from viral-enriched data remains a challenging problem. Our predictions rely on statistical models of evolutionary change which are sensitive to the quality of the multiple alignments they evaluate and some assumptions might not hold for certain proteins. As longer reads become more affordable and viral information in sequence databases increases, the detection of viral ORFs genes will become less complex. However, achieving adequate depth of coverage and lowering error rates remain major issues limiting ORFan gene prediction, and these will have to be addressed by improvements in sequencing technology, viral enrichment techniques, and bioinformatics methods.

## Methods

A schematic overview of the entire analysis scheme except for sample collection and preparation is shown in Fig. 1.

### Sample preparation, library construction, and sequencing

Samples were collected from serum, nasopharyngeal and throat swabs, feces, and cerebrospinal fluid of anonymized patients. The study, including patient sampling and protocols was approved by the local ethics committee at the Karolinska Institute, The Regional Ethical Review Board, Stockholm, Dnr 02–212, 02–422, and 04–836/4. All experiments were performed in accordance with relevant guidelines and regulations.

The samples were processed according to the protocol described in a previous study^7^. Briefly, samples were pooled, centrifuged, enriched for viral particles using filtration and enzymatic digestion, and DNA and RNA were extracted and amplified separately using random-primed PCR. Amplicons between approximately 400 and 1,500 bp were size selected, yielding 17 libraries which were subjected to 454 sequencing^29^. Supplementary Table S1 summarizes the results of sequencing these libraries. In aggregate, almost 4 million reads were sequenced comprising 937 Mbp with an average read length of 240.2 nucleotides.

### Pre-assembly removal of repetitive content and human sequences

To maximize the contig lengths in the assembly and therefore the potential discovery of novel protein families, sequence reads from all 17 libraries, both RNA and DNA fractions, were combined and analysed together. Furthermore, to reduce the risk of misassemblies^30^, highly repetitive and human sequences were removed as follows.

Reads were first analysed using RepeatMasker version 3.2.8 to identify known biological repeats and low-complexity segments. RepeatMasker was run with the parameters ‘-species primates’. Reads were discarded if more than 70% repetitive or if the highest scoring region (under a 1/-5 model for unmasked and masked positions, respectively) was shorter than 50 nucleotides.

To remove human sequence, reads were searched against the NCBI human transcripts and human genome databases^31^ using NCBI BLAST 2.2.23^32^. Reads were discarded if a match was found at ≥90% identity over ≥80% of the query sequence.

### Assembly

Reads were assembled using MIRA 3.0.5^33^ with the parameters ‘-job = denovo, genome, accurate, 454′. From the ACE file produced by the assembler, contigs were extracted into FASTA format, and the following statistics were determined for each contig and included in each FASTA header: number of reads, contig coverage (minimum, maximum, and mean), the samples which the reads derive from, the number of reads originating from DNA and RNA, and the number of reads from each library. Reads assembled into 1,042,291 contigs totalling 290,369,822 nucleotides, with an average length of 278.6 nucleotides.

### Post-assembly filtering of known sequences

To remove contigs which matched known genes, the 1,042,291 assembled reads were used as queries in a blastn search of the NCBI non-redundant nucleotide database (nt). The 613,840 sequences which did not have a match against nt were used as queries in a blastx search of the nr non-redundant protein database. Of those, 479,558 sequences did not have a match in the nr database. The nt and nr databases were downloaded from ftp://ftp.ncbi.nih.gov/blast/db on March 1 and March 10, 2011, respectively. Both searches were performed using NCBI BLAST version 2.2.24+^34^ with a conservative E-value cutoff of 1e-5 (Sayers *et al*. 2010) with the following parameters: ‘-evalue 1e-5 -outfmt 7 -num_descriptions 1 -num_alignments 1 -num_threads 8′.

To expect on average less than one hit by chance when accounting for the number of searches performed, the E-value cutoff was set initially to 1e^−7^ (one hit in (1,042,291 * 2) searches ≈4.8e^−7^). While the E-value is a measure of the false positive rate and not an indication that all true matches will be found, in order to be conservative, we considered as “known” and therefore eliminated from further analysis any database hit which matched one of our query sequences at a significance ~100x lower than that (E = 1e^−5^).

To remove any residual untrimmed tag sequences which could confound clustering, we applied the TagCleaner and PRINSEQ algorithms and to predict and remove tags. We used TagCleaner version 0.11-standalone, obtained from the URL http://tagcleaner.sourceforge.net on April 1, 2011^35^. PRINSEQ is a web-based tool available at http://edwards.sdsu.edu/prinseq_beta/, which we ran with the parameters “{“derep0”:“true”, “derep1”:“true”, “derep2”:“true”, “derep3”:“true”, “derep4”:“true”, “tailsite”:“1”, “trimsite”:“1”, “trimtype”:“2”, “trimrule”:“1”}” on April 1, 2011^36^. After applying TagCleaner and PRINSEQ, 402,288 contigs remained, totalling 97,899,385 nucleotides with an average length of 243.4 nt.

### Clustering unknown sequences

The 402,288 assembled sequence contigs were then aligned to one another with NCBI-BLAST 2.2.25^37^ in an all-versus-all comparison using the command ‘blastall -p blastp -e 1e-5 -m 9 -v 10000 -b 10000 -a 8 -K 100000′.

The all-versus-all BLAST results were preprocessed for use with MCL version 10–20138 using the UNIX command-line tools grep and cut to extract the relevant columns from the tabular BLAST output (“grep–no-filename -v “#“ -v blast_tab.txt | cut -f 1, 2, 11 > blast_tab.abc”) and the program mcxload (included with MCL) using the parameters “–stream-mirror–stream-neg-log10 -stream-tf ‘ceil (200)”.

We chose to run MCL with an inflation value (−I) of 6 and -scheme = 7 after experimenting with a range of inflation, scheme, and k-nearest-neighbor settings, finding that the improvement in clustering performance plateaued with those parameters. The jury scores, which measure how accurately the mcl process was computed (see http://micans.org/mcl/man/mclfaq.html), were high across most of the range of inflation values, specifically 87, 95, and 98 out of 100 for the parameters we chose. MCL produced 248,813 clusters, from which we selected for analysis those with sizes between 3 and 250 sequences, yielding a total of 13,861. Highly similar sequences, which are likely duplicate copies of the same sequence with sequence errors, are interpreted incorrectly by RNAcode as independent instances and cause poor performance. To eliminate this source of bias, we sub-clustered the MCL clusters using a single-linkage approach implemented in Perl, aggregating sequences within a cluster that were 95% similar or greater, and then choosing the longest sequence to represent the subcluster. Finally, to reduce the size of the dataset, all clusters greater than size 7 were selected, yielding a total of 456 clusters that were further analyzed.

### Calibrating RNAcode for use with short-read sequence data

Known coding and non-coding nucleotide multiple alignments of *Methanococcus jannaschii* sequence were obtained from http://www.tbi.univie.ac.at/papers/SUPPLEMENTS/RNAcode/. Custom Perl code was used to derive four pairs of known coding and non-coding sequence test sets consisting of alignments 25, 50, 75, and 100 nucleotides in length. The number of alignments in each set were as follows: 25 nt coding, 470; 25 nt non-coding, 160; 50 nt coding, 542; 50 nt non-coding, 181, 75 nt coding, 564; 75 nt non-coding, 102; 100 nt coding, 570; 100 nt non-coding, 43. Each alignment was used as input to RNAcode, and the P-values were plotted versus their length to create Supplementary Figure 1.

### Prediction of novel protein families

For each cluster created by MCL, we made multiple alignments of the DNA sequences corresponding to that cluster using a codon-aware aligner, MACSE v0.8b2 (Ranwez *et al*. 2011), with the parameters “-s -60 -f -10 -g -10” based on the author’s recommendation to lower frameshift and stop codon penalties in less reliable sequence data.

Alignments were trimmed or split manually where necessary, discarding low quality alignments with e.g. poor overlap, low-complexity sequences, areas of homopolymer runs, low sequence variation, etc. The remaining alignments were used as input to RNAcode version 0.3pre^39^, downloaded from http://github.com/wash/rnacode. The software was run using default parameters on all the multiple alignments. To gather the statistics used in Supplementary Table S2, sequences in the multiple alignment were translated into amino acids according to the frame predicted by RNAcode using CodonAlign (http://www.hiv.lanl.gov/content/sequence/CodonAlign/codonalign.html) and custom Perl code. Seg^40^ was run with default parameters on the resulting predicted amino acid sequences, and sequences more than 50% low-complexity were discarded. For all RNAcode predictions with a P-value ≤ 0.15, a combined score was calculated by summing the log_10_ of the RNAcode score, the seg complexity score in bits, and the log_10_ of the predicted ORF length. This composite score was then used to rank predictions. In addition to reporting segments of high coding potential and the corresponding P-values, RNAcode generates diagrams of the multiple alignments with codons annotated as synonymous, conservative, or nonsynonymous changes, as shown in Fig. 2d and Supplemental Fig. 2.

### Similarity searches of putative novel ORFan families

To assess whether the predicted novel protein families showed any significant matches to other metagenomic sequences or known genes, we compared them against four nucleotide databases and four protein databases, as follows: (1) All of the nucleotide sequences from the predicted protein families were searched against the NCBI nt and env_nt databases (downloaded on March 2016), the Human Microbiome Project Reference Genomes Data assembly nucleotide fasta (http://hmpdacc.org/HMRGD/) and the MetaHIT gene catalogue (Li *et al*. 2014) using blastn from Blast 2.2.28+^34^ with the parameters: -max_target_seqs. 10 -outfmt 11. Alignments were post-filtered to an E-value cutoff of E ≤ 1e^−3^. (2) All of the predicted amino acid sequences from the predicted protein families were searched against the NCBI nr and env_nr (March 2016) databases, the Human Microbiome Project Reference Genomes Data (HMPRGD) assembly peptide fasta and the peptide version of the MetaHIT gene catalogue using blastp from Blast 2.2.28+ with the parameters: –max_target_seqs. 10 -outfmt 11. Alignments were post-filtered to an E-value cutoff of E ≤ 1e^−3^. (3) Amino acid multiple alignments from all of the predicted protein families were searched against the four aforementioned protein databases separately using hmmsearch from the HMMER3 suite, version 3.1b2^41^. A cutoff of 10^−3^ for sequence and domain E-value was used as the criterion for statistical significance.

We also searched the predicted novel families against the Rfam database^42^, submitting nucleotide sequences to the Rfam batch sequence search server with default parameters (E-value cutoff of E < 0.01) at http://rfam.sanger.ac.uk/search#tabview=tab1 on December 13, 2011.

### Initial assembly of a cluster 179a-derived amplicon from an NGS library

Primers directed towards the ends were designed from several of the clusters based on the possibility that they could be derived from short, circular genomes. For cluster 179a, the primers were: CTACAACACCGGGAATAAAGTTATACGTCA and GTTTAAGTCGTCGCCGAAGTTTCTTT. A long-range PCR using standard conditions resulted in an approximately 5 kb product.

Fragments were sequenced with the Illumina MiSeq, yielding a total of 3 640 000 raw reads of length 2 × 300. Reads were quality filtered with nesoni clip version 0.126 (https://github.com/Victorian-Bioinformatics-Consortium/nesoni), parameters –min qual 30,–min-length 75 and later assembled with the Iterative Virus Assembler (IVA) version 0.11.0^43^ with default parameters. From the assembled contigs, we extracted the contig of length which contained our primer sequences. The quality of the contig was verified by mapping back the quality filtered Illumina reads with BWA-MEM version 0.7.12^44^ using default parameters and visualizing alignments with the Integrative Genomics Viewer^45^.

### Gap closing of 179a-derived amplicon

The amplification strategy consisted in two overlapping amplicons covering the complete viral genome. Primer set was designed using sequence information obtained by NGS, inspecting highly conserved genomic regions of the IVA assembly. Two DNA amplicons of a length around 2–3 Kb were expected. PCR kit used was Platinum Taq DNA Polymerase High Fidelity (Cat. No. 11304–102, Thermo Fisher Scientific). PCR conditions were 2 minutes at 94 °C for initial denaturalization; 30 cycles of seconds at 94 °C (denature), 30 seconds at 59.5 °C (annealing) and 4 minutes at 68 °C (extension); and a final extension hold of 4 minutes at 68 °C. Non-template controls were also included to discard contamination. After PCR, DNA products were inspected by DNA agarose gel electrophoresis and purified using illustra GFX PCR DNA and Gel Band Purification Kit (Cat. No. 28–9034–70, GE Healthcare Life Sciences). DNA concentration was then quantified using Qubit® dsDNA HS Assay Kit (Cat. No. Q32854, Thermo Fisher Scientific). PCR products obtained were sequenced by capillary sequencing after adjusting DNA concentration. Sequencing primers were also placed in highly conserved genomic regions of the IVA assembly. This resulted in 11 sequencing reactions aiming to cover the entire circular genome in both directions. DNA sequences and chromatograms were manually inspected, and combined with the original contig from the Illumina data they formed a single circular contig using the Sequencher Software (Gene Codes).

### Annotation of bacteriophage HFM

Naive ORF prediction was carried out with EMBOSS version 6.5.7^46^ getorf tool with parameters ‘-circular Y -find 1’,which lists all amino acid sequences between a start and a stop codon in the 6 open reading frames. The resulting 52 putative ORFs were size filtered (length >50) and evaluated against the following database searches: 1) blastp against NCBI nr and env_nr, 2) blastp against MetaHIT^19^, 3) blastp against the HMP HMPRGD peptide database 4) hmmscan against Pfam release 29^47^ and 5) hmmscan against vFam-A build February 2014^48^. To determine the final set of ORFs, any ORF with a database hit with query coverage >80%, sequence id >50% and e-value <1 were kept, yielding a total of 7 ORFs. Then, all hits for the selected ORFs were inspected manually to determine the most likely annotation. The ORF6 protein sequence and all 1657 sequences in the Pfam full alignment of Phage_F (PF02305) were aligned to the Pfam HMM with hmmalign. Unalignable regions in the N- and C-terminus were removed, keeping a conserved core region of about 150 residues. Sequences with >50% gap residues were removed, and sequences >90% identical to other sequences were removed, leaving 331 representative and non-redundant sequences. 54 of these had proper taxonomic annotation (all as Microviridae) and were used to calculate a maximum likelihood tree using RAxML with 1000 bootstraps using the PROTCATBLOSUM62 model.

### Complementary virus prediction strategies

The complementary virus detection strategy was done by combining three tools: Kraken^22^, Kaiju^24^ and Metaphlan2^23^. Metaphlan2 version 2.6.0 was run with default parameters and database. Kraken version 0.10.5-beta was run using the MiniKraken database available from the website (https://ccb.jhu.edu/software/kraken/) with default parameters, and results were filtered using the kraken-filter script with a 0.05 threshold. Kaiju version 1.5.0 was run in greedy mode with parameters ‘-x –m 20 –a greedy –e 1′ against a Progenomes[ref] + RefSeq viral database downloaded in June 2017. Kaiju viral hits were filtered to contain at least 2 sequences matching the same viral species with a custom python script.

### Follow-up PCR in individual patient samples

The presence of the identified virus in individual samples was determined by PCR and capillary sequencing. Primers placed in highly conserved regions were designed as above. Of note, two rounds of amplifications were designed in case not enough DNA yield was obtained after first PCR. Here, primers mapped against the capsid region amplifying a DNA fragment of 267 bp and 155 bp in the first and second PCR, respectively. Before inspecting the individual samples, the presence of the virus in extracted DNA from the DNA pools previously tested by NGS was also determined. They corresponded to FESC A + C, FESC B and FESC D pools. In here, only FESC D library became positive by PCR. Then, viral determinations were performed in the 10 individual samples merged in FESC D pool (samples F31–45). PCR products obtained in both first and Nested PCR were then column-purified and capillary sequenced to confirm the specificity of the amplification. Of note, DNA fragments not corresponding to the amplicon size expected but with similar length than the size expected were also purified and capillary sequenced. After sequence inspection, the virus was only determined in 1 out 10 reactions (sample F32), where the contigs obtained from first and Nested PCR overlapped after sequence trimming, and perfectly mapped against the contig generated by NGS.

### Data availability

All sequencing datasets generated and analysed during this study are available in the European Nucleotide Archive under project accession number PRJEB17838. Additionally, the assembled and annotated sequence of the bacteriophage HFM is available with accession number ERZ376945 under the same project. Scripts to generate this work are available at https://github.com/maubarsom/ORFan-proteins.

## Electronic supplementary material

Supplementary Information
