# Supplementary material for: Discovering viral genomes in human metagenomic data by predicting unknown protein families
Source: Sci Rep. 2018 Jan 8;8:28. doi: 10.1038/s41598-017-18341-7 (PMC5758519; doi:10.1038/s41598-017-18341-7)
Supplement: Supplementary file 1 — Supplementary Information [file 41598_2017_18341_MOESM1_ESM.pdf]

Discovering viral genomes in human metagenomic data by predicting unknown protein families

Mauricio Barrientos-Somarribas, David N. Messina, Christian Pou, Fredrik Lysholm, Annelie Bjerkner, Tobias Allander, Björn Andersson and Erik L.L. Sonnhammer

Supplementary Material

Supplementary Figure S1. Detection of high-confidence protein families by clustering and RNAcode calibration. a) Distribution of cluster sizes from MCL. The size distribution was split into three categories: singletons, clusters of size two, and clusters of size 3 and above (candidate for multiple sequence alignment) b) Comparison of cluster size distribution for the filtered MCL clusters (3 < size < 250) before and after subclustering. Cluster size decreases post-subclustering, indicating that the original clusters contained many sequences with low diversity c) Calibration of RNAcode for short sequences. The plot describes the distribution of RNAcode's p-values for simulated alignments of varying lengths from coding and non-coding regions of the *Methanococcus jannaschii* genome. RNAcode separates coding from non-coding sequences up to a length of 50bp with a p-value of 0.15.

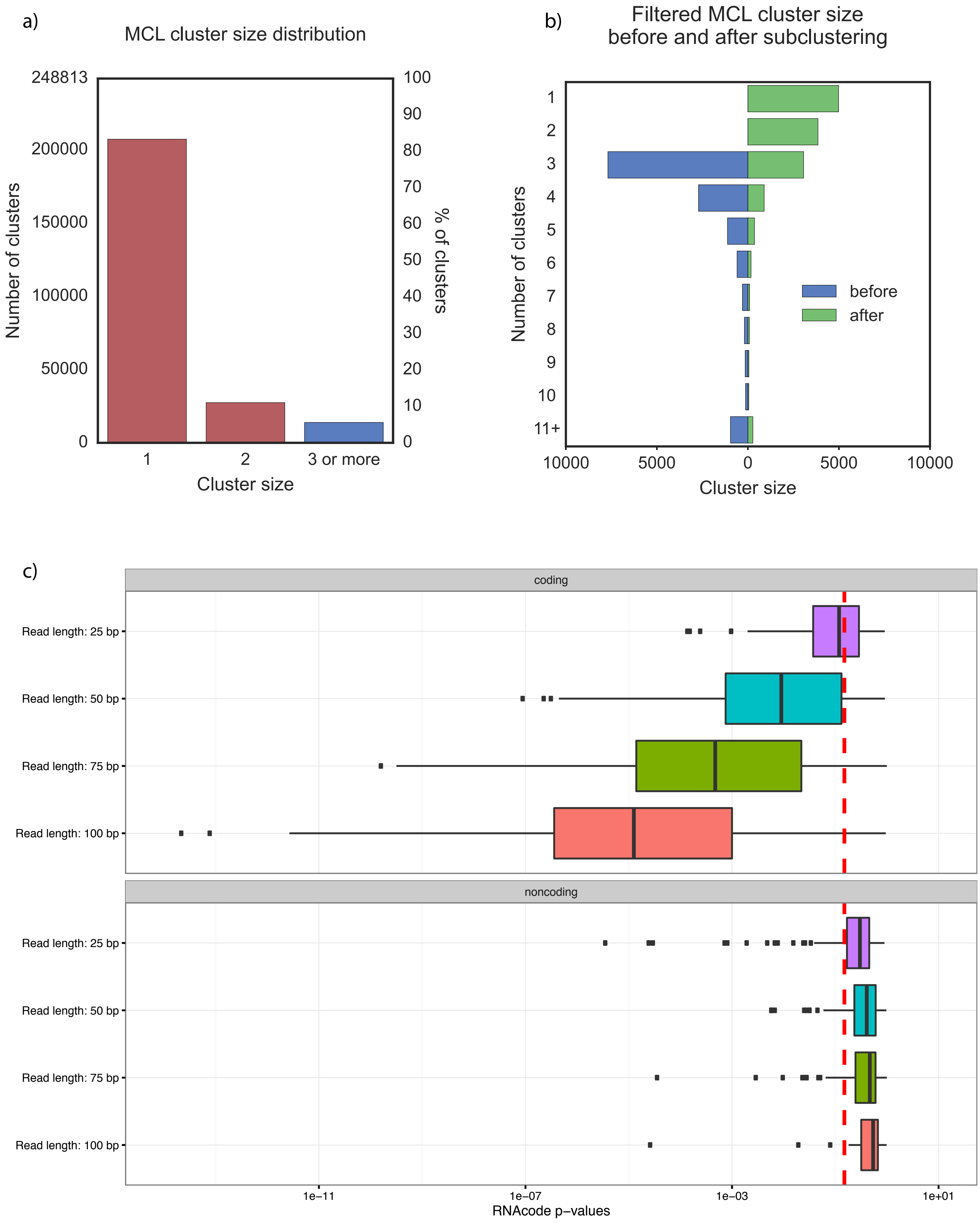
a)
MCL cluster size distribution
Number of clusters
% of clusters
248813
200000
150000
100000
50000
0
100
90
80
70
60
50
40
30
20
10
0
1
2
3 or more
Cluster size
b)
Filtered MCL cluster size
before and after subclustering
Number of clusters
1
2
3
4
5
6
7
8
9
10
11+
before
after
10000
5000
0
5000
10000
Cluster size
c)
coding
noncoding
Read length: 25 bp
Read length: 50 bp
Read length: 75 bp
Read length: 100 bp
1e−11
1e−07
1e−03
1e+01
RNAcode p−values

Supplementary Figure S2. RNAcode output for the 32 predicted protein families.

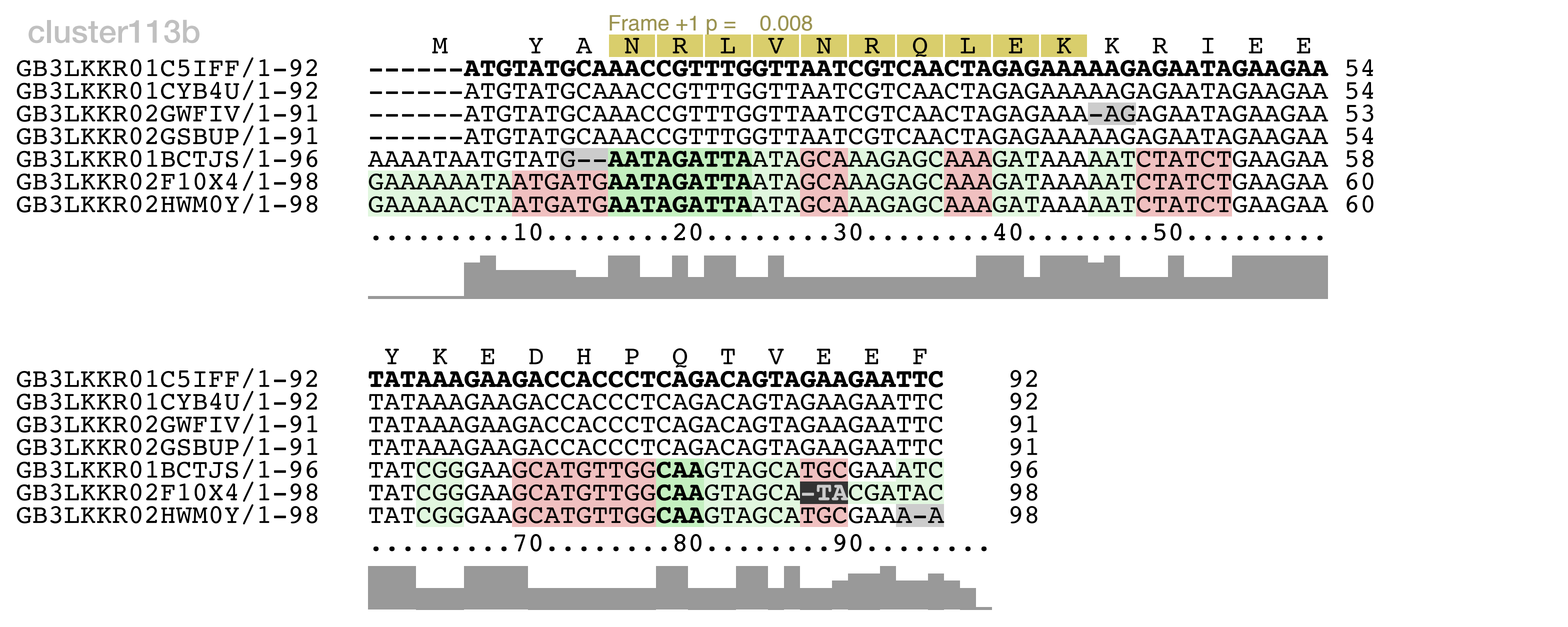

cluster113b
Frame +1 p = 0.008
M Y A N R L V N R Q L E K K R I E E
GB3LKKR01C5IFF/1-92 ------ATGTATGCAAACCGTTTGGTTAATCGTCAACTAGAGAAAAAGAGAATAGAAGAA 54
GB3LKKR01CYB4U/1-92 ------ATGTATGCAAACCGTTTGGTTAATCGTCAACTAGAGAAAAAGAGAATAGAAGAA 54
GB3LKKR02GWFIV/1-91 ------ATGTATGCAAACCGTTTGGTTAATCGTCAACTAGAGAAA-AGAGAATAGAAGAA 53
GB3LKKR02GSBUP/1-91 ------ATGTATGCAAACCGTTTGGTTAATCGTCAACTAGAGAAAAAGAGAATAGAAGAA 54
GB3LKKR01BCTJS/1-96 AAAATAATGTATG--AATAGATTAATAGCAAAGAGCAAAGATAAAAATCTATCTGAAGAA 58
GB3LKKR02F10X4/1-98 GAAAAAATAATGATGAATAGATTAATAGCAAAGAGCAAAGATAAAAATCTATCTGAAGAA 60
GB3LKKR02HWM0Y/1-98 GAAAAACTAATGATGAATAGATTAATAGCAAAGAGCAAAGATAAAAATCTATCTGAAGAA 60
.........10........20........30........40........50.........
Y K E D H P Q T V E E F
GB3LKKR01C5IFF/1-92 TATAAAGAAGACCACCCTCAGACAGTAGAAGAATTC 92
GB3LKKR01CYB4U/1-92 TATAAAGAAGACCACCCTCAGACAGTAGAAGAATTC 92
GB3LKKR02GWFIV/1-91 TATAAAGAAGACCACCCTCAGACAGTAGAAGAATTC 91
GB3LKKR02GSBUP/1-91 TATAAAGAAGACCACCCTCAGACAGTAGAAGAATTC 91
GB3LKKR01BCTJS/1-96 TATCGGGAAGCATGTTGGCAAGTAGCATGCGAAATC 96
GB3LKKR02F10X4/1-98 TATCGGGAAGCATGTTGGCAAGTAGCA-TACGATAC 98
GB3LKKR02HWM0Y/1-98 TATCGGGAAGCATGTTGGCAAGTAGCATGCGAAA-A 98
.........70........80........90........

cluster179a

```
GB3LKKR01D9539/1-314                                                                 257
GB3LKKR01B7VFA/1-313                                                                 256
GB3LKKR01A1AS6/1-174                                                                 175
GB3LKKR01DZB8X/1-177                                                                 136
GB3LKKR02JP9RY/1-314                                                                 257
                        .........10........20........30........40........50.........

                                  *  I  I  K  V  V  V  L  I  L  F  Y     V  S  A  F
GB3LKKR01D9539/1-314              TAGATAATAAAGGTGGTGGTGCTTATACTCTTTT---ATGTATCCGCGTT 197
GB3LKKR01B7VFA/1-313              TAGATAATAAAGGTGGTGGTGCTTATACTTCTTT---ATGTATCCGCGTT 196
GB3LKKR01A1AS6/1-174              TAGATAATAACGGTGCTGCACCGTATACTTTCTTATTATGTATCCGCGTC 175
GB3LKKR01DZB8X/1-177              -------------------------------------------------- 76
GB3LKKR02JP9RY/1-314              TAGATAATTCTGGTCCAACTTCGTACACTTCTTT---ATGTATCCGCGTC 197
                        .........70........80........90........100.......110........

                                          Frame -2 p =   0.005
                         R  Y  C  C  F  F  S  Q  I  M  P  F  N  H  R  A  L  P  N
GB3LKKR01D9539/1-314    CCGGTACTGCTGCTTTTTCAGTCAGATTATGCCGTTTAATCATCGTGCTGCACTTCCTAA 137
GB3LKKR01B7VFA/1-313    CCGGTACTGCTGCTTTTTCAGTCAGATTATGCCGTTTAATCATCGTGCTGCACTTCCAAA 136
GB3LKKR01A1AS6/1-174    CCGGTGTTGCTGCATTTTCAGTCAGGTTATGCCGTTTAATCATCGTGCTGCACTTCCAAA 127
GB3LKKR01DZB8X/1-177    -------TTCTGCATTTTCAGTCAGGTTATGCCGTTTAATCATCGTGCTGCACTTCCAAA 16
GB3LKKR02JP9RY/1-314    CCGGTGTTGCTGCATTTTTAGTCAGGTTATGCCGTTTAATCGTCGTGCTGCACTTCCAAA 137
                        .........130.......140.......150.......160.......170........

                           S  L  M  S  W  F  R  I  A  N  S  P  I  F  N  Y  S  D  N  P
GB3LKKR01D9539/1-314    TAGTCTGATGTCTTGGTTTCGTATTGCTAATAGTCCGATTTTTAATTATTCTGATAATCC 80
GB3LKKR01B7VFA/1-313    TAGTTTGATGTCGTGGCTTCGTATTGCTAATAGTCCTATTATCAATTATCCTAAGAACAC 79
GB3LKKR01A1AS6/1-174    TAGGTTGATA-------------------------------------------------- 67
GB3LKKR01DZB8X/1-177    TAGTTTGATGTCGTGGCTTCGTATTGCTAATAGTCCTATTCTCAATTATCCTAAGAATAC 1
GB3LKKR02JP9RY/1-314    TAGTTTGATGTCGTGGCTTCGTATTGCTAATAGTCCTATTATCAATTATCCTAAGAACAC 80
                        .........190.......200.......210.......220.......230........

                           L  P  S  G  G  V  L  F  K  S  S  P  K  F  L  S  V  N  A  D
GB3LKKR01D9539/1-314    TTTGCCTTCTGGTGGAGTTTTGTTTAAGTCGTCGCCGAAGTTTCTTTCTGTGAATGCAGA 23
GB3LKKR01B7VFA/1-313    TGTGCCTACTACTGATGCTCTTCTCAAGACATCGGCGAAGTTTGTTACTGTGAATGCAGA 22
GB3LKKR01A1AS6/1-174    ------------------------------------------------------------ 7
GB3LKKR01DZB8X/1-177    TCTGCCTATTGATAGTTCTCTTCTCAAGACAGCGTCGAAGTTTCTTACTGTGAATGCAGA 1
GB3LKKR02JP9RY/1-314    TGTGCCTACTACTGATGCTCTTCTCAAGACATCGGCGAAGTTTGTTACTGTGAATGCAGA 23
                        .........250.......260.......270.......280.......290........

                           T  Y  L  G  Y  W
GB3LKKR01D9539/1-314    TACTTATTTAGGTTATTGG    1
GB3LKKR01B7VFA/1-313    TACTTATTTAGGTTATTGG    1
GB3LKKR01A1AS6/1-174    -------------------    1
GB3LKKR01DZB8X/1-177    TACT---------------    1
GB3LKKR02JP9RY/1-314    TACTTATTTAGGTTATTGG    1
                        .........310.......320
```

cluster179b

```
                                                            Frame -1 p =   0.010
                                         *  I  S  I  C  I  H  S  K  K  L  R  R  C
GB3LKKR01C2BL0/1-231                   TAAATAAGTATCTGCATTCACAGTAAGAAACTTCGACGCTG 172
GB3LKKR01EB2BL/1-168                   TAAATAAGTATCTGCATTCACAGTAAGAAACTTCGACGCTG 169
GB3LKKR02ITVHK/1-237                   TAAATAAGTATCTGCATTCACAGTAACAAACTTCGCCGATG 179
GB3LKKR02JWM4O/1-158                   TAAATAAGTATCTGCACTCACAGAAAGAAACTTCGGCGACG 159
GB3LKKR02IKPFQ/1-162                   ----------------------------------------- 103
                       .........10........20........30........40........50.........

                        L  E  K  R  T  I  N  R  Q  S  I  L  R  I  I  E  N  R  T  I
GB3LKKR01C2BL0/1-231   TCTTGAGAAGAGAACTATCAATAGGCAGAGTATTCTTAGGATAATTGAGAATAGGACTAT 112
GB3LKKR01EB2BL/1-168   TCTTGAGAAGAGAACTATCAATAGGCAGAGTATTCTTAGGATAATTGAGAATAGGACTAT 119
GB3LKKR02ITVHK/1-237   CCTTGAGAAGAGCATCAGTAGTAGGCACAGTGTTCTTAGGATAATTGATAATAGGACTAT 119
GB3LKKR02JWM4O/1-158   ACTTAAACAAAACTCCACCAGTAGGCACAGTGTTCTTAGGATAATTGATAATAGGACTAT 119
GB3LKKR02IKPFQ/1-162   ----------------ACCAGAAGGCAAAGGATTACCAGAATAATTAAAAATCGGACTAT 43
                       .........70........80........90........100.......110........

                        S  N  T  K  P  R  H  Q  T  I  W  K  C  S  T  M  I  K  R  H
GB3LKKR01C2BL0/1-231   TAGCAATACGAAGCCACGACATCAAACTATTTGGAAGTGCAGCACGATGATTAAACGGCA 52
GB3LKKR01EB2BL/1-168   TAGCAATACGAAGCCACGACATCAAACTATTTGGAAGTGCACCACGAT------------ 59
GB3LKKR02ITVHK/1-237   TAGCAATACGAAGCCACGACATCAAACTATTTGGAAGTGCAGCACGATGATTAAACGGCA 59
GB3LKKR02JWM4O/1-158   TAGCAATACGAAGCCACGACATCAAACTATTTGGAAGT---------------------- 59
GB3LKKR02IKPFQ/1-162   TAGCAATACGAAGCCAAGACATCAGACTATTAGGAAGTGCAGCACGATGATTAAACGGCA 1
                       .........130.......140.......150.......160.......170........

                        N  L  T  E  K  C  S  N  T  G  T  R  I  H  I  R  S  I  R
GB3LKKR01C2BL0/1-231   TAACCTGACTGAAAAATGCAGCAACACCGGGACGCGGATACATATAAGAAGTATACGG 1
GB3LKKR01EB2BL/1-168   ---------------------------------------------------------- 1
GB3LKKR02ITVHK/1-237   TAACCTGACTAA-AAATGCAGCAACACCGGGACGCGGATACATAAAAGAAGTGTACGA 1
GB3LKKR02JWM4O/1-158   ---------------------------------------------------------- 1
GB3LKKR02IKPFQ/1-162   TAATCTGACTGAAAAAAGCAGCAGTACCGGAACGCGGATACATAAAAGAAGTATAAGC 1
                       .........190.......200.......210.......220.......230......
```

cluster182a

```
GB3LKKR02H8MH7/1-364                                                                306
GB3LKKR01C3KIQ/1-328                                                                269
GB3LKKR02H78NR/169-302                                                              75
GB3LKKR02I75TX/1-273                                                                214
GB3LKKR01AM7C8/22-109                                                               30
GB3LKKR02IRLPC/358-499                                                              83
contig08842/31-253                                                                  164
GB3LKKR02H8NEG/1-364                                                                305
GB3LKKR01EC368/1-365                                                                306
contig04034/238-604                                                                 308
                         .........10........20........30........40........50.........

GB3LKKR02H8MH7/1-364                                                                247
GB3LKKR01C3KIQ/1-328                                                                209
GB3LKKR02H78NR/169-302                                                              15
GB3LKKR02I75TX/1-273                                                                154
GB3LKKR01AM7C8/22-109                                                               1
GB3LKKR02IRLPC/358-499                                                              23
contig08842/31-253                                                                  104
GB3LKKR02H8NEG/1-364                                                                245
GB3LKKR01EC368/1-365                                                                247
contig04034/238-604                                                                 248
                         .........70........80........90........100.......110........

GB3LKKR02H8MH7/1-364                                                                188
GB3LKKR01C3KIQ/1-328                                                                149
GB3LKKR02H78NR/169-302                                                              1
GB3LKKR02I75TX/1-273                                                                95
GB3LKKR01AM7C8/22-109                                                               1
GB3LKKR02IRLPC/358-499                                                              1
contig08842/31-253                                                                  44
GB3LKKR02H8NEG/1-364                                                                185
GB3LKKR01EC368/1-365                                                                188
contig04034/238-604                                                                 188
                         .........130.......140.......150.......160.......170........

                                                                    *  R  T  Q  K  P
GB3LKKR02H8MH7/1-364                                                TAACGGACTCAAAAACC 128
GB3LKKR01C3KIQ/1-328                                                TAACGGACTCAAAAACC 89
GB3LKKR02H78NR/169-302                                              ----------AAAATCC 1
GB3LKKR02I75TX/1-273                                                TAACGGACTCAAAAACC 35
GB3LKKR01AM7C8/22-109                                               ----------------- 1
GB3LKKR02IRLPC/358-499                                              --CGGACCTTAAAAACC 1
contig08842/31-253                                                  AACGGCCTTAAAAAACC 1
GB3LKKR02H8NEG/1-364                                                TAACGGACTCAAAAACC 126
GB3LKKR01EC368/1-365                                                TAACGGACTCAAAAACC 128
contig04034/238-604                                                 TAACGGTCTTAAAAACC 128
                         .........190.......200.......210.......220.......230........

                                                  Frame -1 p =  5.2e-04
                           A  K  T  N  A  T  S  M  G  V  R  K  E  G  M  G  L  Q  Y
GB3LKKR02H8MH7/1-364     AGCAAAAACTAATGCAACAAGCATGGGAGTACGAAAAG-AGGGGATGGGTCTACAATACA 68
GB3LKKR01C3KIQ/1-328     AGCAAAAACTAATGCAACAAGCATGGGAGTACGAAAAAGAGGGGATGGGTCTACAATACA 29
GB3LKKR02H78NR/169-302   AGCAAAAACTAATGCAACAAGCATGGGAGTACGAAAAAGAGGGGATGGGTCTACAATACA 1
GB3LKKR02I75TX/1-273     AGCAAAAACTAATGCAACAAGCATGGGAGTACGAAAAAGAGGGGATGGGTCTACAATACA 1
GB3LKKR01AM7C8/22-109    -------------------------------------GAAGAAG-TGGGGCTACAATATA 1
GB3LKKR02IRLPC/358-499   AACAAAAACTAATGCAACAGGCATGGGAGTATGAAAAAGAAGGCATGGGTCTGCAATATA 1
contig08842/31-253       AACAAAAACTAATGCAACAAGCGTGGGAGTATGAAAAAGAAGGAATGGGGCTACAATATA 1
GB3LKKR02H8NEG/1-364     AGCAAAAACTAATGCAACAAGCATGGGAGTACGAAAAAGAGGGGATGGGTCTACAATACA 67
GB3LKKR01EC368/1-365     AGCAAAAACTAATGCAACAAGCATGGGAGTACGAAAAA-AGGGGATGGGTCTACAATACA 68
contig04034/238-604      AGCAAAAACTAATGAAACAAGCATGGGAATACGAAAAGGAAGGAATGGGCCTGCAATACA 68
                         .........250.......260.......270.......280.......290........

                         N  Y  G  Q  Q  A  A  D  A  E  Y  K  R  N  L  Q  M  W  K  E
GB3LKKR02H8MH7/1-364     ACTATGGGCAACAAGCAGCAGACGCTGAATATAAACGAAATCTACAAATGTGGAAAGAAA 8
GB3LKKR01C3KIQ/1-328     ACTATGGGCAACAAGCAGCAGACGCTGAATATAAACGAAATCTACAAATGTGGAAAGAAA 1
GB3LKKR02H78NR/169-302   ACTATGGGCAACAAGCAGCAGACGCTGAATATAAACGAAATCTACAAATGTGGAAAGAAA 1
GB3LKKR02I75TX/1-273     ACTATGGGCAACAAGCAGCAGACGCTGAATATAAACGAAATCTACAAATGTGGAAAGAAA 1
GB3LKKR01AM7C8/22-109    ATTACGGACAACAAGCGGCAGACGCTGAATACAAGAGAAATCTAGCAGCAAAC-AAGACA 1
GB3LKKR02IRLPC/358-499   ATTACGGACAACAAGCGGCAGATGCAGAGTATAAACGAAATCTGCAGATGTGGAAAGACA 1
contig08842/31-253       ATTACGGACAACAAGCGGCAGACGCTGAATACAAGAGAAATCTGCAAATGTGGAAAGACA 1
GB3LKKR02H8NEG/1-364     ACTATGGGCAACAAGCAGCAGACGCTGAATATAAACGAAATCTACAAATGTGGAAAGAAA 7
GB3LKKR01EC368/1-365     ACTATGGGCAACAAGCAGCAGACGCTGAATATAAACGAAATCTACAAAGGTGGAAAGAAA 8
contig04034/238-604      ACTATGGACAACAAGCAGCAGACGCTGAATACAAACGAAACCTTCAGATGTGGAAAGATA 8
                         .........310.......320.......330.......340.......350........

                         T  N
GB3LKKR02H8MH7/1-364     CCAAC    1
GB3LKKR01C3KIQ/1-328     CCAAC    1
GB3LKKR02H78NR/169-302   CCAAC    1
GB3LKKR02I75TX/1-273     CCAAC    1
GB3LKKR01AM7C8/22-109    GTAGC    1
GB3LKKR02IRLPC/358-499   CCAAT    1
contig08842/31-253       CCAAC    1
GB3LKKR02H8NEG/1-364     CCAAC    1
GB3LKKR01EC368/1-365     CCAAC    1
contig04034/238-604      CTAAT    1
                         .......
```

cluster182b

```
GB3LKKR02JSIBW/4-194                                                          30
GB3LKKR02G4CF7/1-191                                                          55
GB3LKKR02GLIMM/1-188                                                          18
GB3LKKR02JJI0V/1-222                                                          54
GB3LKKR02JAAI8/38-264                                                         60
GB3LKKR01EDLON/5-176                                                          59
GB3LKKR02GK10E/263-445                                                        60
GB3LKKR02IIMCY/1-227                                                          59
contig10047/1-228                                                             60
GB3LKKR01DC008/1-71                                                           0
GB3LKKR02J3V1O/1-105                                                          0
                          .........10........20........30........40........50.........

                                                                 Frame +2 p =  1.5e-04
                                           *  C  N  K  R  G  S  M  K  R  G  M  G
GB3LKKR02JSIBW/4-194                       TAATGCAACAAGCGTGGGAGTATGAAAA-G-AGGAATGGGG--  82
GB3LKKR02G4CF7/1-191                       TAATGCAACAAGCGTGGGAGTATGAAAAAGAAGGAATGGGG--  113
GB3LKKR02GLIMM/1-188                       CCAAAACAATTGCCGAAGCAAATAAAATCGCAGGA-GTGGACA  77
GB3LKKR02JJI0V/1-222                       TAATGCAACAAGCATGGGAGTATGAAAAAGAGGGAATGGGT--  112
GB3LKKR02JAAI8/38-264                      TAATGCAACAGGCATGGGAGTATGAAAAAGAAGGCATGGGT--  118
GB3LKKR01EDLON/5-176                       TAATGCAACAAGCGTGGGAGTATGAAAAAGAAGGAATGGGG--  117
GB3LKKR02GK10E/263-445                     TAATGCAACAAGCATGGGAGTACGAAAAGAGGGGGATGGGT--  118
GB3LKKR02IIMCY/1-227                       TAATGCAACAAGCATGGGAGTACGAAAAAGNGGGGATGGGG--  117
contig10047/1-228                          TAATGCAACAAGCATGGGAGTACGAAAAAGAGGGGATGGGT--  118
GB3LKKR01DC008/1-71                        -------------------------------------------  0
GB3LKKR02J3V1O/1-105                       -------------------------------------------  0
                          .........70........80........90........100.......110........

                          L   Q  Y  N  Y  G  Q  Q  A  A  D  A  E  Y  K  R  I  C  K
GB3LKKR02JSIBW/4-194      -CTACAATATAATTACGGACAACAAGCGGCAGACGCTGAATACAAGAG-AATCTGCAAAT 140
GB3LKKR02G4CF7/1-191      -CTACAATATAATTACGGACAACAAGCGGCAGACGCTGAATACAAGAGAAATCTGCAAAT 172
GB3LKKR02GLIMM/1-188      CAGAAGGGGCAAAACTAGACAACGAATGGAAGACGCTGAATACAAGAGAAATCTGCAAAT 137
GB3LKKR02JJI0V/1-222      -CTGCAATACAACTATGGACAACAAGCGGCAGATGCTGAATACAAGCGGAACCTACAAAT 171
GB3LKKR02JAAI8/38-264     -CTGCAATATAATTACGGACAACAAGCGGCAGATGCAGAGTATAAACG-AATCTGCAGAT 176
GB3LKKR01EDLON/5-176      -CTACAATATAATTACGGACAACAAGCGGCAGACGCTGAATACAAGAGAAATCTGC---- 172
GB3LKKR02GK10E/263-445    -CTACAATACAACTATGGGCAACAAGCAGCAGACGC-GAATATAAACGAAATCTAAAATG 176
GB3LKKR02IIMCY/1-227      TCTACAATACAACTATGGGCAACAAGCAGCAGACACTGAATATAAACG-AATCTACAAAT 176
contig10047/1-228         -CTACAATACAACTATGGGCAACAAGCAGCAGACGCTGAATATAAACGAAATCTACAAAT 177
GB3LKKR01DC008/1-71       ----------------------------------------TATAAACGAAATCTGCAGAT 20
GB3LKKR02J3V1O/1-105      -ATATCGCGAGATCATGGGCAACAAGCAGCAGACGCTGAATATAAACGAAATCTACAAAT 59
                          .........130.......140.......150.......160.......170........

                          C  G  K  T  P  T  L  E  H  K  E  P  R  W  K  T  Q
GB3LKKR02JSIBW/4-194      GTGGAAAGACACCAACTTTGGAGCACAAAGAGCCGAGATGGAAAACGCAG  191
GB3LKKR02G4CF7/1-191      GTGGAAAGACACCAACTTT-------------------------------  191
GB3LKKR02GLIMM/1-188      GTGGAAAGACACCAACTTTGGAGCACAAAGAGCCGAGATGGAAAACGCAG  188
GB3LKKR02JJI0V/1-222      GTGGAAAGACACCAACTTTGGAGCACAAAGAGCCGAGATGGAAGATGCAG  222
GB3LKKR02JAAI8/38-264     GTGGAAAGACACCAATTTTGGAGCACAAAGAGACGAAATGGAGAAAGCGG  227
GB3LKKR01EDLON/5-176      --------------------------------------------------  172
GB3LKKR02GK10E/263-445    TAGGAAA-------------------------------------------  183
GB3LKKR02IIMCY/1-227      GTGGAAAGAAACCAACTTTGGAGCGCAAAGGAATGAAATGGAAAAAGCAG  227
contig10047/1-228         GTGGAAAGAAACCAACTTTGGAGCGCAAAGGAATGAAATGGAAAAAGCAG  228
GB3LKKR01DC008/1-71       GTGGAAAGACACCAATTTTGGAGCACAAAGAGACGAAATGGAGAAAGCGG  71
GB3LKKR02J3V1O/1-105      GTGGAAAGAAACCAACTTTGGAGCGCAAAGGAATGAAATGGAAAAA----  105
                          .........190.......200.......210.......220.......23
```

cluster211a

Frame -3 p = 3.9e-06

```
                   A  V  A  G  G  T              Y  R  D  W  L  E  T  V
GB3LKKR01EWCHU/1-169    GCAGTAGCAGGAGGTAC---------------ATACAGGGATTGGTTGGAAACAGTGT 110
GB3LKKR02IEAKO/31-199   GCAGTAGCAGGAGGTAC---------------ATACAGGGATTGGTTGGAAACAGTGT 110
GB3LKKR02FP5VJ/14-182   GCAGTAGCAGGAGGTAC---------------ATACAGGGATTGGTTGGAAACAGTGT 110
GB3LKKR02F78VO/1-176    ATGCTAAACAGAATTGCAGTAAGCGGTGGAACTTATAGAGATTGGTTAGAAACAGTAT 125
                      .........10........20........30........40........50.........

                      Y  T  A  G  K  Y  L  D  R  P  E  T  P  V  F  I  G  G  M  T
GB3LKKR01EWCHU/1-169  ACACAGCAGGAAAATACCTTGACAGGCCCGAAACACCTGTATTTATCGGAGGTATGACAC 50
GB3LKKR02IEAKO/31-199 ACACAGCAGGAAAATACCTTGACAGGCCCGAAACACCTGTATTTATCGGAGGTATGACAC 50
GB3LKKR02FP5VJ/14-182 ACACAGCAGGAAAATACCTTGACAGGCCCGAAACACCTGTATTTATCGGAGGTATGACAC 50
GB3LKKR02F78VO/1-176  ATACGGCAGGAAAATACCTTGACAGACCGGAAACACCGGTATTTATCGGCGGTATGACCC 65
                      .........70........80........90........100.......110........

                      Q  Y  I  E  F  D  E  V  I  S  K  S  A  T  E  S  L  Q  H  A
GB3LKKR01EWCHU/1-169  AATATATCGAATTCGACGAAGTGATTTCAAAAAGTGCGACAGAAAGTTTACAACATGCTA 5
GB3LKKR02IEAKO/31-199 AATATATCGAATTCGACGAAGTGATTTCAAAAAGTGCGACAGAAAGTTTACAACATGCTA 5
GB3LKKR02FP5VJ/14-182 AATATATCGAATTCGACGAAGTGATTTCAAAAAGTGCGACAGAAAGTTTACAACATGCTA 5
GB3LKKR02F78VO/1-176  AATATATCGAGTTCGATGAAGTAATTTCAAAATCAGCAACAGAAACAATATACGGC---- 5
                      .........130.......140.......150.......160.......170........

                      K
GB3LKKR01EWCHU/1-169  AA   1
GB3LKKR02IEAKO/31-199 AA   1
GB3LKKR02FP5VJ/14-182 AA   1
GB3LKKR02F78VO/1-176  --   1
                      ....
```

cluster211b

```
                                                     *  V  N  N  G  R  T  Q  L  A
GB3LKKR02FZEFS/108-325                              TAAGTTAACAATGGACGCACTCAACTTGCAA 60
GB3LKKR02I4GU4/159-256                              TAAGTTAACAATGGACGCACTCAACTTGCAA 60
GB3LKKR01A3RF7/9-215                                TAAATTAACAATGGATGCACTCAACTTGCAG 60
GB3LKKR01EE5WR/1-203                                GAAATTGACAATGGACGCGCTGAACCTGCAA 44
GB3LKKR02JMT8K/92-304                               CAGTCTATCAATGGACGCACTCAACTTAGCA 54
contig03712/344-517                                 AAAATTAACAATGGATGCGCTAAACCTGCAA 60
GB3LKKR01EP7G6/106-331                              AAGAATT-AAGTGAAACAAAGAAAGTTTAGA 59
                         .........10.........20.........30.........40.........50.........

                                        Frame +3 p =  3.4e-14
                         T  K  V  Y  N  M  L  N  R  I  A           V           S  G
GB3LKKR02FZEFS/108-325   CAA-AAGTTTACAACATGTTAAACAGGATCGCA------------------GTAAGTGGT 101
GB3LKKR02I4GU4/159-256   CAAAAAGTT-TCAACATGTTAAACAGGATCGCA------------------GTAAGT--- 98
GB3LKKR01A3RF7/9-215     CAAAAAGTTTACAACATGCTAAACAGAATTGCA------------------GTAAGCGGT 102
GB3LKKR01EE5WR/1-203     CAGAAAGTTTACAACATGCTAAACAGGATCGCA------------------GTATCAGGA 86
GB3LKKR02JMT8K/92-304    CAAAAAGTTTACAACATGTTAAACAGAATTGCA------------------GTAAGTGGC 96
contig03712/344-517      CAGAAAGTTTACAACATGCTAAACAGAATCGCA------------------GTAGCAGGA 102
GB3LKKR01EP7G6/106-331   AAGGAATACTACTACATGTTCTATGAATTATATACTAAGAGAATGTCAGCA-GAAGCGGT 118
                         .........70.........80.........90.........100.......110........

                         G  T  Y  R  D  W  L  E  T  V  Y  T  A  G  K  Y  L  D  R  P
GB3LKKR02FZEFS/108-325   GGAACATATAGGGATTGGCTGGAAACAGTATATACGGCGGGAAAGTACCTTGACAGGCCG 161
GB3LKKR02I4GU4/159-256   ------------------------------------------------------------ 98
GB3LKKR01A3RF7/9-215     GGAACTTATAGAGATTGGTTAGAAACAGTATATACGGCAGGAAAATACCTTGACAGA-CG 161
GB3LKKR01EE5WR/1-203     GGTACCTACCGAGACTGGCTGGAAACAGTGTATACAGCAGGAAAATATCTTGACAGACCC 146
GB3LKKR02JMT8K/92-304    GGAACATACAGAGACTGGTTAGAAACAGTATTCACAGGCGGAGAATACATGGAAAGATGT 156
contig03712/344-517      GGTACATACAGGGATTGGTTGGAAACAGTGTACACAGCAGGAAAATACCTTGACAGGCCC 162
GB3LKKR01EP7G6/106-331   GGAACTTATAGAGATTGGTTAGAAACAGTATATACGGCAGGAAATAACCTTGACAGACCG 178
                         .........130.......140.......150.......160.......170........

                         E  T  P  V  F  I  G  G  M  T  Q  Y  I  E  F  D  E  V  I
GB3LKKR02FZEFS/108-325   GAAACACCGGTATTTATCGGCGGCATGACTCAATACATTGAATTCGATGAAGTGATC 218
GB3LKKR02I4GU4/159-256   --------------------------------------------------------- 98
GB3LKKR01A3RF7/9-215     GAAACACCGGTA-TTATCGGGG-TATGAC-CAATATATCGAGTTCGATG-------- 207
GB3LKKR01EE5WR/1-203     GAAACACCTGTGTTTATCGGAGGTATGACACAATATATCGAATTCGACGAAGTGATC 203
GB3LKKR02JMT8K/92-304    GAAACACCAATATTCGAAGGTGGAACAAGCCAAGAAATTGTATTTCAAGAAGTAATC 213
contig03712/344-517      GAAACACCTGTA--------------------------------------------- 174
GB3LKKR01EP7G6/106-331   GAAACACCGGTATTTATCGGCGGTATGACCCAATATATCGAGTTCGCT--------- 226
                         .........190.......200.......210.......220.......230.....
```

cluster241a

Frame -3 p = 0.002

```
                         H  Y  Q  C  E  E  P  G  Y  I  M  G  L  M  A  I  T  P  M
GB3LKKR02IEC5V/306-457    CACTACCAATGCGAAGAGCCGGGATACATTATGGGGCTAATGGCAATCACACCGATGA 95
GB3LKKR01EIO7Z/1-110      CACTACCAATGTGAAGAACCGGGCTACATTATGGGATTAATGGCAATCACACCGATGA 95
GB3LKKR02HOXTL/22-174     CACTACCAATGCGAAGAGCCGGGATACATTATGGGGCTAATGGCAATCACACCGATGA 95
GB3LKKR01DJQRC/358-498    -----------CGAAGAGCCGGGATACATTATGGGGCTAATGGCAATCACACCGATGA 82
GB3LKKR01CUDIG/48-200     AATTACCTATGCGAAGAGCCGGGATACATTATGGGGCTAATGGCAATCACACCGATGA 95
                        .........10.........20.........30.........40.........50.........

                        I  D  Y  S  Q  G  N  D  F  D  L  N  L  Q  T  I  D  D  L  H
GB3LKKR02IEC5V/306-457  TTGATTATTCACAAGGAAACGACTTTGATCTAAACTTGCAAACAATAGATGATCTGCATA 35
GB3LKKR01EIO7Z/1-110    TTGATTATTCGCAAGGAAACGACTTTGATTTAAACTTGCAAACAATGGAT---------- 35
GB3LKKR02HOXTL/22-174   TTGATTATTCACAAGGAAATGACTTTGATTTAAACTTGCAAACAATGGATGATCTCCATA 35
GB3LKKR01DJQRC/358-498  TTGATTATTCACAAGGAAATGACTTTGATTTAAACTTGCAAACAATGGATGATCTCCATA 22
GB3LKKR01CUDIG/48-200   TTGATTATTCACAAGGAAATGACTTTGATTTAAACTTGCAAACAATGGATGATCTCCATA 35
                        .........70.........80.........90.........100.......110........

                        K  P  A  L  D  G  I  G  Y  Q  D
GB3LKKR02IEC5V/306-457  AACCGGCATTAGATGGTATCGGCTACCAGGAT     1
GB3LKKR01EIO7Z/1-110    --------------------------------     1
GB3LKKR02HOXTL/22-174   AACCGGCACTAGATGGAATTGAAGATTAACAG     1
GB3LKKR01DJQRC/358-498  AACCGGCACTAGATGGAATTGGATACCAAGAC     1
GB3LKKR01CUDIG/48-200   AACCGGCACTAGATGGAATTGGATACCAAGAC     1
                        .........130.......140.......150..
```

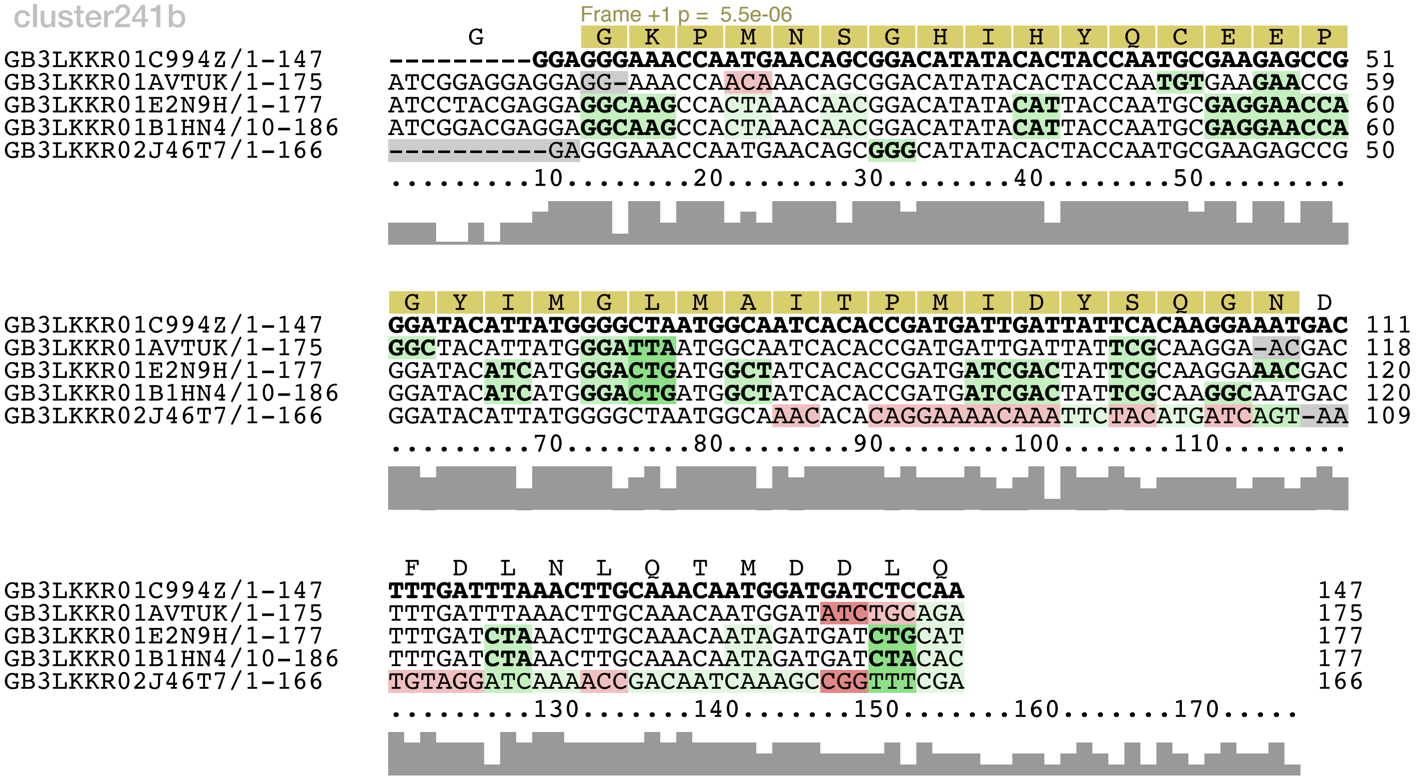
cluster241b
Frame +1 p = 5.5e-06
G G K P M N S G H I H Y Q C E E P
GB3LKKR01C994Z/1-147 ---------GGAGGGAAACCAATGAACAGCGGACATATACACTACCAATGCGAAGAGCCG 51
GB3LKKR01AVTUK/1-175 ATCGGAGGAGGAGG-AAACCAACAAACAGCGGACATATACACTACCAATGTGAAGAACCG 59
GB3LKKR01E2N9H/1-177 ATCCTACGAGGAGGCAAGCCACTAAACAACGGACATATACATTACCAATGCGAGGAACCA 60
GB3LKKR01B1HN4/10-186 ATCGGACGAGGAGGCAAGCCACTAAACAACGGACATATACATTACCAATGCGAGGAACCA 60
GB3LKKR02J46T7/1-166 ----------GAGGGAAACCAATGAACAGCGGGCATATACACTACCAATGCGAAGAGCCG 50
.........10.........20.........30.........40.........50.........
G Y I M G L M A I T P M I D Y S Q G N D
GB3LKKR01C994Z/1-147 GGATACATTATGGGGCTAATGGCAATCACACCGATGATTGATTATTCACAAGGAAATGAC 111
GB3LKKR01AVTUK/1-175 GGCTACATTATGGGATTAATGGCAATCACACCGATGATTGATTATTCGCAAGGA-ACGAC 118
GB3LKKR01E2N9H/1-177 GGATACATCATGGGACTGATGGCTATCACACCGATGATCGACTATTCGCAAGGAAACGAC 120
GB3LKKR01B1HN4/10-186 GGATACATCATGGGACTGATGGCTATCACACCGATGATCGACTATTCGCAAGGCAATGAC 120
GB3LKKR02J46T7/1-166 GGATACATTATGGGGCTAATGGCAAACACACAGGAAAACAAATTCTACATGATCAGT-AA 109
.........70.........80.........90.........100.........110.........
F D L N L Q T M D D L Q
GB3LKKR01C994Z/1-147 TTTGATTTAAACTTGCAAACAATGGATGATCTCCAA 147
GB3LKKR01AVTUK/1-175 TTTGATTTAAACTTGCAAACAATGGATATCTGCAGA 175
GB3LKKR01E2N9H/1-177 TTTGATCTAAACTTGCAAACAATAGATGATCTGCAT 177
GB3LKKR01B1HN4/10-186 TTTGATCTAAACTTGCAAACAATAGATGATCTACAC 177
GB3LKKR02J46T7/1-166 TGTAGGATCAAAACCGACAATCAAAGCCGGTTTCGA 166
.........130.........140.........150.........160.........170.....

cluster258

```
                                    Frame +3 p =   0.015
                          K  V  P  F  Y  R  K  K  S  F  W  T  M  L  I  S  I  L  T
contig07739/468-671       A-AGGTACCGTTTTATCGTAAGAAGAGCTTTTGGACAATGTTGATTTCCATTCTTACG 59
GB3LKKR02GPGDJ/44-247     A-AAGTACCGTTTTATCGTAAGAAGAGTTTTTGGACATTGTTGATTTCCATTCTTACG 59
GB3LKKR01B1BAU/42-243     A--GGTACCGTTTTATCGTAA-AAGAGTTTTTGGACAATGTTGATTTCCATTCTTACG 57
GB3LKKR01CQ42S/1-190      -------------TATCGTAAGAAGAGTTTTTGGACATTGTTGATTTCCATTCTTACG 45
GB3LKKR02GPY0A/70-273     ACTCGTACCGTTTTATCGTAAGAAGAG-TTTTGGACATTGTTGATTTCCATTCTTACG 59
GB3LKKR02HCE5B/72-255     AGAAGCTTCGGATTACGTTCGTTTGTTGTTTGAACAGTATCCC--------------- 45
GB3LKKR01CZX7K/74-257     AGAAGCTTCGGATTACGTTCGTTTGTTGTTTGAACAGTATCCC--------------- 45
                        .........10........20........30........40........50.........
```

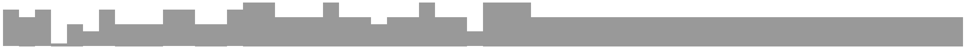

```
                         A  L  S  V  Y  F  A  S  S  C  S  R  K  L  V  Y  R  S  S  G
contig07739/468-671     GCTTTATCTGTTTATTTTGCTTCTTCATGTAGTCGGAAGTTGGTTTATCGTTCTTCCGGT 119
GB3LKKR02GPGDJ/44-247   GCATTATCTGTTTATTTTGCAGCTTCATGTAATCGTAAGATTGTTTATCGTTCTTCCGGT 119
GB3LKKR01B1BAU/42-243   GCTTTATCTGTTTATTTTGCTGCTTCATGTACTCGGAAGTTGGTTTATCGTTCTTCCGGT 117
GB3LKKR01CQ42S/1-190    GCATTATCTGTTTATTTTGCAGCTTCATGTAATCGTAAGATTGTTTATCGTTCTTCCGGT 105
GB3LKKR02GPY0A/70-273   GCATTATCTGTTTATTTTGCAGCTTCATGTAATCGTAAGATTGTTTATCGTTCTTCCGGT 119
GB3LKKR02HCE5B/72-255   ------GGTATTTATTTTGCTGCTTCATGTACTCGGAAGTTGGTTTATCGTTCTTCCGGT 99
GB3LKKR01CZX7K/74-257   ------GGTATTTATTTTGCTGCTTCATGTACTCGGAAGTTGGTTTATCGTTCTTCCGGT 99
                        .........70........80........90........100.......110........
```

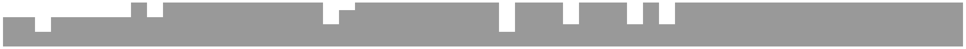

```
                         V  H  C  D  T  V  Q  L  D  V  R  S  N  L  K  L  P  *  Y  A
contig07739/468-671     GTTCATTGTGATACGGTTCAACTTGATGTTCGGTCTAATTTAAAACTTCCTTAATATGCC 179
GB3LKKR02GPGDJ/44-247   GTTCATTGTGACACGGTTCAACTTGATGTTCGGTCTAATTTAAAACTTCCTTAATATGCC 179
GB3LKKR01B1BAU/42-243   GTTCATTGTGATACTGTTCAAGTTGATGTTCGGTCTAATTTAAAGCTTCCTTAATATGCC 177
GB3LKKR01CQ42S/1-190    GTTCATTGTGACACGGTTCTATTTGATGTTCGGTCTAATTTAAAACTTCCTTAATATGCC 165
GB3LKKR02GPY0A/70-273   GTTCATTGTGACACGGTTCTACTTGATGTTCGGTCTAATTTAAAACTTCCTTAATATGCC 179
GB3LKKR02HCE5B/72-255   GTTCATTGTGATACTGTTCAAGTTGATGTTCGGTCTAATTTAAAGCTTCCTTAATATGCC 159
GB3LKKR01CZX7K/74-257   GTTCATTGTGATACTGTTCAAGTTGATGTTCGGTCTAATTTAAAGCTTCCTTAATATGCC 159
                        .........130.......140.......150.......160.......170........
```

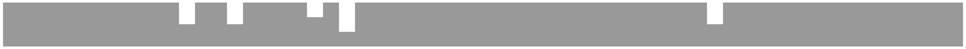

```
                         W  C  *
contig07739/468-671     TGGTGCTAG                 204
GB3LKKR02GPGDJ/44-247   TGGTGCTAG                 204
GB3LKKR01B1BAU/42-243   TGCTGCTGG                 202
GB3LKKR01CQ42S/1-190    TGCTGCTGG                 190
GB3LKKR02GPY0A/70-273   TGCTGCTGG                 204
GB3LKKR02HCE5B/72-255   TGCTGCTGG                 184
GB3LKKR01CZX7K/74-257   TGCTGCTGG                 184
                        .........190.......200...
```

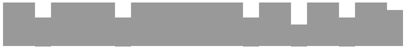

cluster297a

```
                        V  K  D  L  G  F  V  W  I  A  S  I  T  D  K  R  L  R  Y  V
GB3LKKR02JI9WH/42-359   GTTAAAGATTTAGGTTTTGTTTGGATTGCATCTATTACGGACAAGCGTCTTCGGTATGTT 60
GB3LKKR02J4V1J/161-340  GTTAAGGATTTAGGTTTTGTTTGGATTGCATCTATTACGGACAAGCGTCTTCGGTATGTT 60
GB3LKKR02IA3U3/37-354   GTTAAGGATTTAGGTTTTGTTTGGATTGCATCTATTACGGACAAGCGTCTTCGGTATGTT 60
GB3LKKR02JGGMM/177-494  GTTAAGGATTTAGGTTTTGTTTGGATTGCATCTATTACGGACAAGCGTCTTCGGTATGTT 60
GB3LKKR01AFJJ4/82-399   GTTAAGGATTTAGGTTTTGTTTGGATTTCATCTATTACGGACAAGCGTCTTCGGTATGTT 60
                        .........10.........20.........30.........40.........50.........

                        V  K  Y  V  G  K  S  V  Y  M  D  E  R  S  A  D  F  A  K  S
GB3LKKR02JI9WH/42-359   GTCAAGTATGTTGGCAAGTCTGTTTATATGGATGAGCGTTCTGCAGATTTTGCGAAGTCT 120
GB3LKKR02J4V1J/161-340  GTCAAGTATGTTGGCAAGTCTGTTTATATGGATGAGCGTTCTGCAGCTTTTGCGAAGTCT 120
GB3LKKR02IA3U3/37-354   GTCAAGTATGTTGGCAAGTCTGTTTATATGGATGAGCGTTCTGCAGCTTTTGCGAAGTCT 120
GB3LKKR02JGGMM/177-494  GTCAAGTATGTTGGCAAGTCTGTTTATATGGATGAGCGTTCTGCAGCTTTTGCGAAGTCT 120
GB3LKKR01AFJJ4/82-399   GTGAAGTATGTTGGCAAGTCTGTTTATATGGATGAGCGTTCTGTCGGTTTTGCGAAGTCT 120
                        .........70.........80.........90.........100.......110........

                        L  P  I  T  V  G  K  L  K  T  N  L  Y  D  F  L  Q  N  S  R
GB3LKKR02JI9WH/42-359   CTTCCTATTACTGTAGGTAAATTAAAAACTAATCTTTATGACTTTCTTCAGAATAGTAGA 180
GB3LKKR02J4V1J/161-340  CTTCCTATTACTGTAGGTAAATT-AAAACTAATCTTTATGACTTTCTTCAGAATAGTAGA 179
GB3LKKR02IA3U3/37-354   CTTCCTATTACTGTAGGTAAATTAAAAACTAATCTTTATGACTTTCTTCAGAATAGTAGA 180
GB3LKKR02JGGMM/177-494  CTTCCTATTACTGTAGGTAAATTAAAAACTAATCTTTATGACTTTCTTCAGAATAGTAGA 180
GB3LKKR01AFJJ4/82-399   CTTCCTATTACTGTAGGTAAATTAAAAACTAATCTTTATGACTTTCTTCAGAATAGTCGA 180
                        .........130.......140.......150.......160.......170........

                           Frame +1 p =  0.001
                        Y  R  R  K  F  I  S  A  G  V  G  D  Y  F  G  D  F  K  A  P
GB3LKKR02JI9WH/42-359   TATCGCCGTAAATTTATTTCGGCAGGTGTTGGCGATTATTTTGGGGATTTTAAAGCTCCC 240
GB3LKKR02J4V1J/161-340  T----------------------------------------------------------- 180
GB3LKKR02IA3U3/37-354   TATCGTCGTAAATTTGTTTCACCAGGTGTTGGTGATTATTTTGGCGATTTTGAAGCTCCC 240
GB3LKKR02JGGMM/177-494  TATCGTCGTAAATTTGTTTCACCAGGTGTTGGTGATTATTTTGGCGATTTTGAAGCTCCC 240
GB3LKKR01AFJJ4/82-399   TATCGCCGTAAATTTATTTCGGCAGGTGTTGGCGATTATTTGGGAGATTTTAAAGCTCCC 240
                        .........190.......200.......210.......220.......230........

                        G  V  T  S  G  L  W  S  Y  T  D  H  Q  T  G  V  V  Y  R  Y
GB3LKKR02JI9WH/42-359   GGTGTTACTTCTGGTCTTTGGTCTTACACAGATCATCAGACTGGCGTTGTTTATCGGTAC 300
GB3LKKR02J4V1J/161-340  ------------------------------------------------------------ 180
GB3LKKR02IA3U3/37-354   GGTGTTTCTTCTGGTCTTTGGTCTTACACAGATTATAAGACCGGTGTTGTTTATCGTTAC 300
GB3LKKR02JGGMM/177-494  GGTGTTTCTTCTGGTCTTTGGTCTTACACAGATTATAAGACCGGTGTTGTTTATCGTTAC 300
GB3LKKR01AFJJ4/82-399   AGTGTTGCTTCTGGTCTTTGGTCTTACACAGATCATAAGACCGGTGCTGTTTATCGGTAC 300
                        .........250.......260.......270.......280.......290........

                        R  I  P  R  Y  Y
GB3LKKR02JI9WH/42-359   CGCATCCCTCGCTATTAC 318
GB3LKKR02J4V1J/161-340  ------------------ 180
GB3LKKR02IA3U3/37-354   CGTATCCCTCGCTACTAC 318
GB3LKKR02JGGMM/177-494  CGTATCCCTCGCTACTAC 318
GB3LKKR01AFJJ4/82-399   CGCATCCCTCGCTACTAC 318
                        .........310......
```

cluster297b

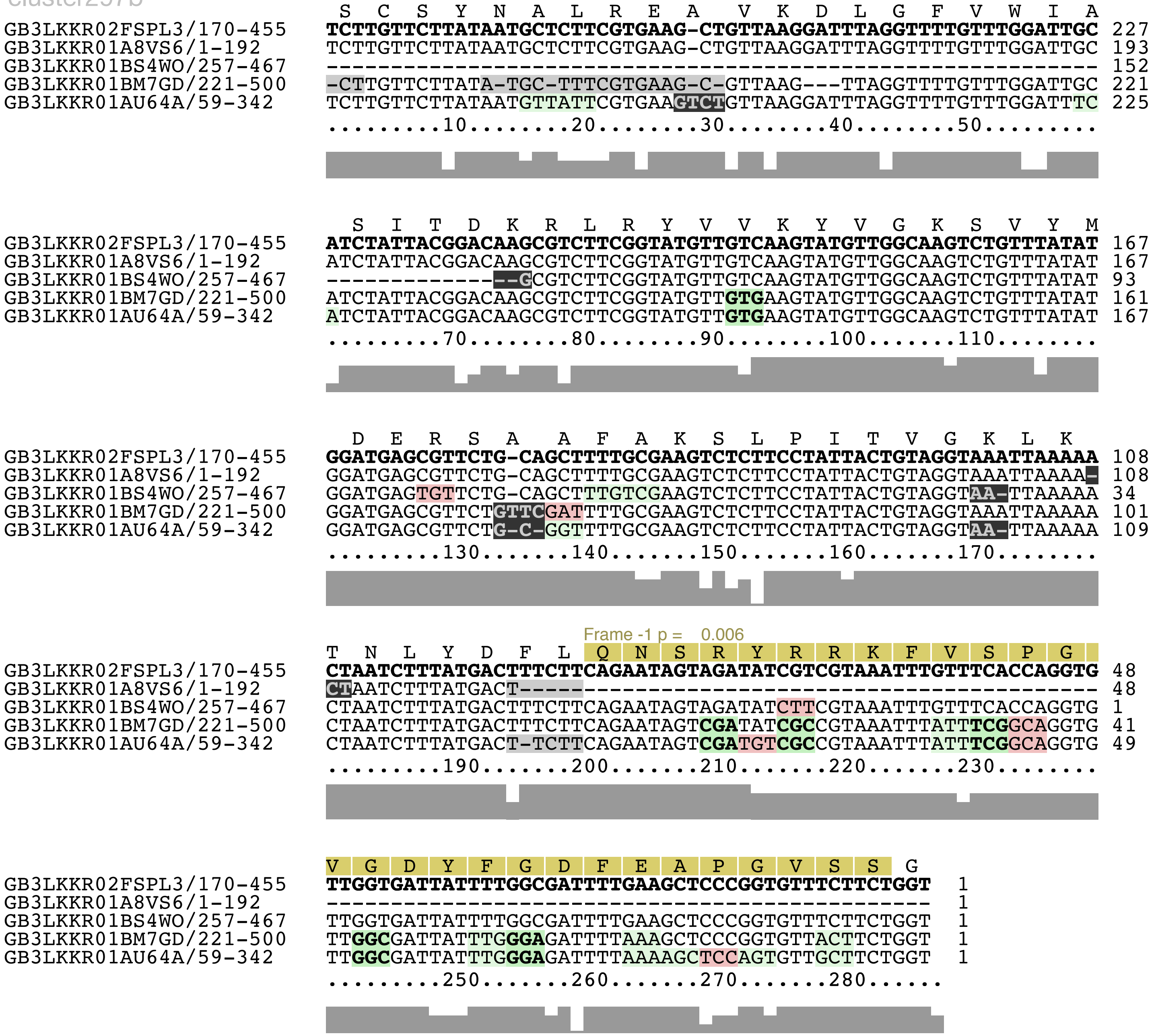

cluster321b

```
                         Frame -1 p =  1.9e-07
                         V  S  A  T  I  P  T  S  S  G  G  A  P  S  G  H  G  P  V  A
GB3LKKR01EYJBS/82-414    GTTTCTGCTACTATTCCCACTTCCAGCGGTGGTGCTCCGTCTGGACATGGTCCAGTTGCA 277
GB3LKKR02G6OMM/1-335     GTTTCTGCTACTACTCCCACTTCCAGCGGTGGTGCTCCGTCTGGACATGGTCCAGTTGCA 277
contig09464/1-118        GTTTCTGCTACTATTCCCACTTCCAGCGGTGGTGCTCCGTCTGGACATGGTCCAGTTGCA 119
GB3LKKR01E1LJ5/1-175     GTTTCTGCTACTATTCCCACTTCCAGCGGTGGTGCTCCGTCTGGACATGGTCCAGTTGCA 176
GB3LKKR01E2CDT/1-134     -------GGATTGATTGAAGTGGGGCGGGTGGTGCTCCGTCAGGTCATGGTCCGGTTGCT 135
contig16546/1-333        GTTTCTGCTACTATACCCACCTCTAGCGGTGGGGCCCCATCCGGTCATGGTCCTGTGGCT 277
GB3LKKR01CSEX1/105-316   ------------------------------------------------------------ 157
GB3LKKR01D984D/221-413   ------------------------------------------------------------ 137
                         .........10........20........30........40........50.........

                         S  G  S  F  G  G  L  A  A  L  A  G  N  P  S  A  Y  A  D  I
GB3LKKR01EYJBS/82-414    TCTGGTTCCTTTGGTGGTCTTGCTGCTCTTGCTGGTAATCCTTCTGCGTATGCGGATATT 217
GB3LKKR02G6OMM/1-335     TCTGGTTCCTTTGGTGGTCTTGCTGCTCTTGCTGGTAATCCTTCTGCGTATGCGGATATT 217
contig09464/1-118        TCTGGTTCCTTTGGTGGTCTTGCTGCTCTTGCTGGTAATCCTTCTGCGTATGCGGATA-- 119
GB3LKKR01E1LJ5/1-175     TCTGGTTCCTTTGGTGGTCTTGCTGCTCTTGCTGGTAATCCTTCTGCGTATGCGGATATT 176
GB3LKKR01E2CDT/1-134     TCTGGTTCCGGAGGTGGTCTTGCTGCTCTTGCCGGTAATCCTTCTGCGTATGCGGATATT 135
contig16546/1-333        TCTGGTGTCGGTGGGGGTCTTGCTGCTCTTGCTGGTAATCCTTCTGCGTATGCGGATATT 217
GB3LKKR01CSEX1/105-316   ----------------------------------------------------------T 97
GB3LKKR01D984D/221-413   ------------------------------------------------------------ 77
                         .........70........80........90........100.......110........

                         Q  L  K  D  A  Q  Q  D  R  E  R  S  A  A  A  L  N  D  A  E
GB3LKKR01EYJBS/82-414    CAGTTGAAAGATGCTCAGCAGGATCGAGAGCGTTCGGCAGCAGCTCTTAATGATGCTGAA 157
GB3LKKR02G6OMM/1-335     CAGTTGAAAGATGCTCAGCAGGAGCGTGAGCGTTCGGCTGCTTCTCTCAATGATGCTGAG 157
contig09464/1-118        ------------------------------------------------------------ 119
GB3LKKR01E1LJ5/1-175     CAGTTGAAAGACGCTCAGCAGGAGCGTGAGCGTTCGGCTGCTTCTTCTAATTGAT----- 157
GB3LKKR01E2CDT/1-134     CAGTTGAAAGATGCTCAGCAG--------------------------------------- 135
contig16546/1-333        CAGTTGAAAGATGCTCAGCAGGATCGAGAGCGTTCGGCAGCAGCTCTTAATGATGCTGAA 157
GB3LKKR01CSEX1/105-316   CAGTTG-AAGATGCTCAGCAGGAGCGTGAGCGTTCGGCTGCTTCTCTTAATGATGCTGAG 37
GB3LKKR01D984D/221-413   ------------------AGGAGCGTGAGCGTTCGGCTGCTTCTCTTAATGATGCTGAG 18
                         .........130.......140.......150.......160.......170........

                         A  E  W  Y  K  S  Q  T  L  D  K  D  L  R  E  R  L  M  K  A
GB3LKKR01EYJBS/82-414    GCTGAATGGTATAAGTCACAGACTTTGGATAAAGATTTGCGTGAACGTTTGATGAAGGCG 97
GB3LKKR02G6OMM/1-335     GCTGATTGGTATAGGTCCCAGACTTTGGATAAAGATTTGCGTGAACGTCTGATGAAGGCT 97
contig09464/1-118        ------------------------------------------------------------ 97
GB3LKKR01E1LJ5/1-175     ------------------------------------------------------------ 97
GB3LKKR01E2CDT/1-134     ------------------------------------------------------------ 90
contig16546/1-333        GCTGAATGGTATAAGTCACAGACTTTGGATAAAGATTTGCGTGAACGTTTGATGAAGGCG 97
GB3LKKR01CSEX1/105-316   GCTGATTGGTATAGGTCCCAGACTTTGGATAAAGATTTGCGTGAACGTCTGATGAAGGCT 1
GB3LKKR01D984D/221-413   GCTGATTGGTATAGGTC-CAGACTTTGGATAAAGATTTGCGTGAACGTCTGATGAAGGCT 1
                         .........190.......200.......210.......220.......230........

                         Q  A  G  L  A  E  A  G  I  T  E  S  T  S  R  A  S  L  N  T
GB3LKKR01EYJBS/82-414    CAGGCAGGACTTGCAGAAGCTGGAATTACTGAATCTACATCGCGTGCAAGTTTGAACACT 37
GB3LKKR02G6OMM/1-335     CAGGCCGGACTTGCTGAAGCTGGAATCACTGAATCTGCTTCGCGTGCAAGTTTGAATCCT 37
contig09464/1-118        ------------------------------------------------------------ 37
GB3LKKR01E1LJ5/1-175     ------------------------------------------------------------ 37
GB3LKKR01E2CDT/1-134     ------------------------------------------------------------ 30
contig16546/1-333        CAGGCAGGACTTGCAGAAGCTGGAATTACTGAATCTACATCGCGTGCAAGTTTGAACACT 37
GB3LKKR01CSEX1/105-316   CAGGCCGGACTTGCTGAAGCTGGAATCACTGAATCTGCTTCGCGTGCAAGTT-AATGCT 1
GB3LKKR01D984D/221-413   CAGGCCGGACTTGCTGAAGCTGGAATCACTGAATCTGCTTCGCGTGCAAGTTTGAATGCT 1
                         .........250.......260.......270.......280.......290........

                         A  I  T  L  S     Y     T  I  D  N  E
GB3LKKR01EYJBS/82-414    GCGATAACTTTGTCT---TATACTATTGATAACGAG 1
GB3LKKR02G6OMM/1-335     GCTGCTGTTCTTGGTCAGTCTGGAGTTGG-TGCGAT 1
contig09464/1-118        ------------------------------------ 1
GB3LKKR01E1LJ5/1-175     ------------------------------------ 1
GB3LKKR01E2CDT/1-134     ------------------------------------ 1
contig16546/1-333        GCGATAACTTTGTCT---TATACTATTGATAACGAG 1
GB3LKKR01CSEX1/105-316   GCGATAACTTTATCC---TATTCTATTGATAACGAG 1
GB3LKKR01D984D/221-413   GCGATAACTTTATCC---TATTCTATTGATAACGAG 1
                         .........310.......320.......330....
```

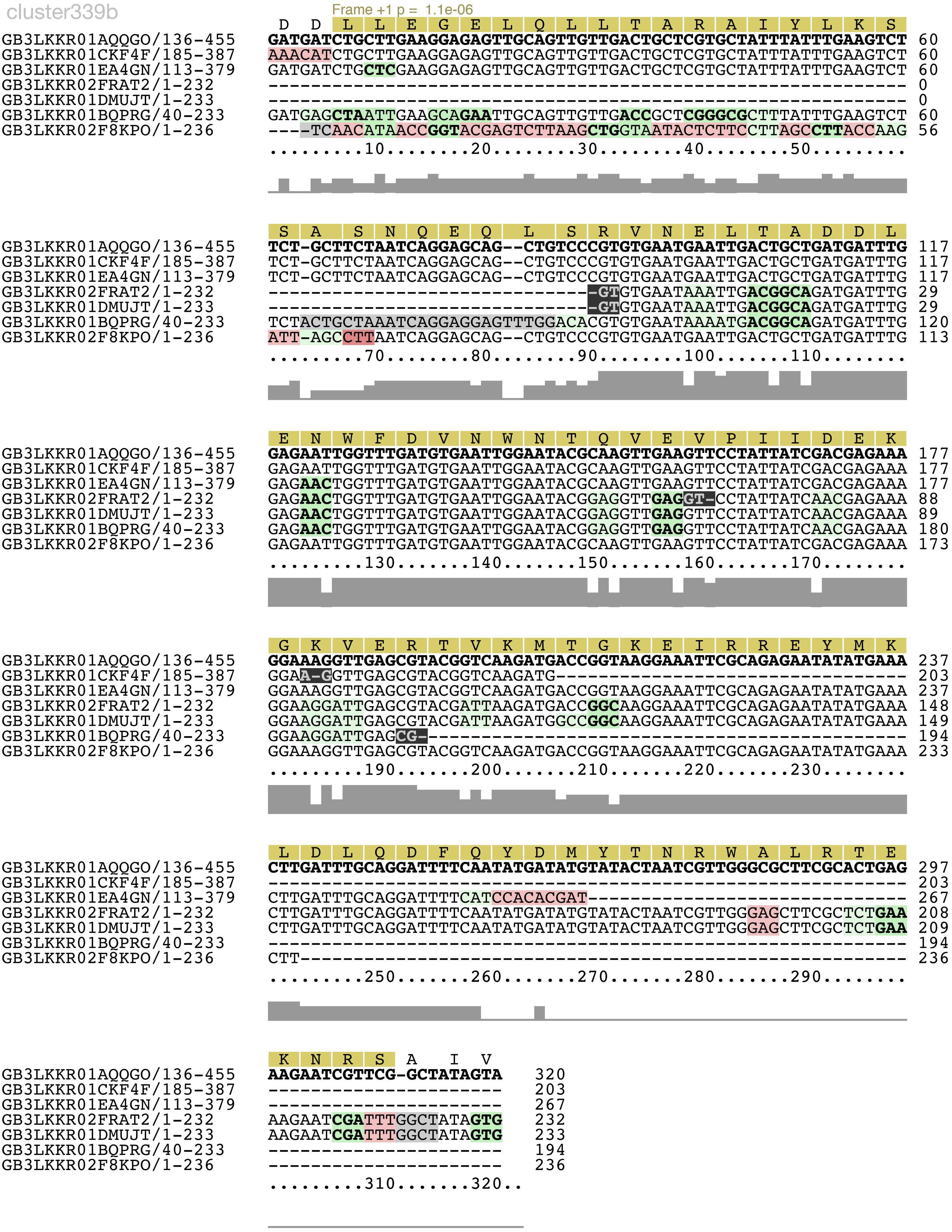
cluster339b
Frame +1 p = 1.1e-06
D D L L E G E L Q L L T A R A I Y L K S
GB3LKKR01AQQGO/136-455 GATGATCTGCTTGAAGGAGAGTTGCAGTTGTTGACTGCTCGTGCTATTTATTTGAAGTCT 60
GB3LKKR01CKF4F/185-387 AAACATCTGCTTGAAGGAGAGTTGCAGTTGTTGACTGCTCGTGCTATTTATTTGAAGTCT 60
GB3LKKR01EA4GN/113-379 GATGATCTGCTCGAAGGAGAGTTGCAGTTGTTGACTGCTCGTGCTATTTATTTGAAGTCT 60
GB3LKKR02FRAT2/1-232 -- 0
GB3LKKR01DMUJT/1-233 -- 0
GB3LKKR01BQPRG/40-233 GATGAGCTAATTGAAGCAGAATTGCAGTTGTTGACCGCTCGGGCGCTTTATTTGAAGTCT 60
GB3LKKR02F8KPO/1-236 ----TCAACATAACCGGTACGAGTCTTAAGCTGGTAATACTCTTCCTTAGCCTTACCAAG 56
S A S N Q E Q L S R V N E L T A D D L
GB3LKKR01AQQGO/136-455 TCT-GCTTCTAATCAGGAGCAG--CTGTCCCGTGTGAATGAATTGACTGCTGATGATTTG 117
GB3LKKR01CKF4F/185-387 TCT-GCTTCTAATCAGGAGCAG--CTGTCCCGTGTGAATGAATTGACTGCTGATGATTTG 117
GB3LKKR01EA4GN/113-379 TCT-GCTTCTAATCAGGAGCAG--CTGTCCCGTGTGAATGAATTGACTGCTGATGATTTG 117
GB3LKKR02FRAT2/1-232 -------------------------------GTGTGAATAAATTGACGGCAGATGATTTG 29
GB3LKKR01DMUJT/1-233 -------------------------------GTGTGAATAAATTGACGGCAGATGATTTG 29
GB3LKKR01BQPRG/40-233 TCTACTGCTAAATCAGGAGGAGTTTGGACACGTGTGAATAAAATGACGGCAGATGATTTG 120
GB3LKKR02F8KPO/1-236 ATT-AGCCTTAATCAGGAGCAG--CTGTCCCGTGTGAATGAATTGACTGCTGATGATTTG 113
E N W F D V N W N T Q V E V P I I D E K
GB3LKKR01AQQGO/136-455 GAGAATTGGTTTGATGTGAATTGGAATACGCAAGTTGAAGTTCCTATTATCGACGAGAAA 177
GB3LKKR01CKF4F/185-387 GAGAATTGGTTTGATGTGAATTGGAATACGCAAGTTGAAGTTCCTATTATCGACGAGAAA 177
GB3LKKR01EA4GN/113-379 GAGAACTGGTTTGATGTGAATTGGAATACGCAAGTTGAAGTTCCTATTATCGACGAGAAA 177
GB3LKKR02FRAT2/1-232 GAGAACTGGTTTGATGTGAATTGGAATACGGAGGTTGAGGT-CCTATTATCAACGAGAAA 88
GB3LKKR01DMUJT/1-233 GAGAACTGGTTTGATGTGAATTGGAATACGGAGGTTGAGGTTCCTATTATCAACGAGAAA 89
GB3LKKR01BQPRG/40-233 GAGAACTGGTTTGATGTGAATTGGAATACGGAGGTTGAGGTTCCTATTATCAACGAGAAA 180
GB3LKKR02F8KPO/1-236 GAGAATTGGTTTGATGTGAATTGGAATACGCAAGTTGAAGTTCCTATTATCGACGAGAAA 173
G K V E R T V K M T G K E I R R E Y M K
GB3LKKR01AQQGO/136-455 GGAAAGGTTGAGCGTACGGTCAAGATGACCGGTAAGGAAATTCGCAGAGAATATATGAAA 237
GB3LKKR01CKF4F/185-387 GGAA-GGTTGAGCGTACGGTCAAGATG--------------------------------- 203
GB3LKKR01EA4GN/113-379 GGAAAGGTTGAGCGTACGGTCAAGATGACCGGTAAGGAAATTCGCAGAGAATATATGAAA 237
GB3LKKR02FRAT2/1-232 GGAAGGATTGAGCGTACGATTAAGATGACCGGCAAGGAAATTCGCAGAGAATATATGAAA 148
GB3LKKR01DMUJT/1-233 GGAAGGATTGAGCGTACGATTAAGATGGCCGGCAAGGAAATTCGCAGAGAATATATGAAA 149
GB3LKKR01BQPRG/40-233 GGAAGGATTGAGCG-- 194
GB3LKKR02F8KPO/1-236 GGAAAGGTTGAGCGTACGGTCAAGATGACCGGTAAGGAAATTCGCAGAGAATATATGAAA 233
L D L Q D F Q Y D M Y T N R W A L R T E
GB3LKKR01AQQGO/136-455 CTTGATTTGCAGGATTTTCAATATGATATGTATACTAATCGTTGGGCGCTTCGCACTGAG 297
GB3LKKR01CKF4F/185-387 -- 203
GB3LKKR01EA4GN/113-379 CTTGATTTGCAGGATTTTCATCCACACGAT------------------------------ 267
GB3LKKR02FRAT2/1-232 CTTGATTTGCAGGATTTTCAATATGATATGTATACTAATCGTTGGGAGCTTCGCTCTGAA 208
GB3LKKR01DMUJT/1-233 CTTGATTTGCAGGATTTTCAATATGATATGTATACTAATCGTTGGGAGCTTCGCTCTGAA 209
GB3LKKR01BQPRG/40-233 -- 194
GB3LKKR02F8KPO/1-236 CTT--- 236
K N R S A I V
GB3LKKR01AQQGO/136-455 AAGAATCGTTCG-GCTATAGTA 320
GB3LKKR01CKF4F/185-387 ---------------------- 203
GB3LKKR01EA4GN/113-379 ---------------------- 267
GB3LKKR02FRAT2/1-232 AAGAATCGATTTGGCTATAGTG 232
GB3LKKR01DMUJT/1-233 AAGAATCGATTTGGCTATAGTG 233
GB3LKKR01BQPRG/40-233 ---------------------- 194
GB3LKKR02F8KPO/1-236 ---------------------- 236

cluster348

```
                       Frame +3 p =  1.4e-08
                          E  I  L  G  L  N  S  T  E  Q  E  E  K  D  N  I  L  R  A
GB3LKKR02JPI9I/6-242        GAGATCCTGGGTCTTAACTCTACTGAGCAAGAAGAAAAAGATAATATTCTTCGAGCTG 60
contig03211/1-228           -------TGGGTCTTAACTCTACTGAGCAAGAAGAAAAAGATAATATTCTTCGAGCTG 51
GB3LKKR02GT5I1/17-126       GTTATTGTGGGTCTTAATTCTACCGAGCAAGAAGAAAAAGATAATATACTTCGAGTTG 60
GB3LKKR01A2JGN/183-393      GTTATCATGGGTCTTAACTCTACTGAGCAAGAAGAGAAAGATAATATTCTTCGAGCTG 60
GB3LKKR02I0PFL/44-279       TTATCATGGGGTCTTAACTCTACTGAGCAAGAAGAAAAAGATAATATTCTTCGAGCTG 60
GB3LKKR02H3Y6W/1-172        ---------------------------------------------------------- 0
GB3LKKR02FQWKY/1-228        ------ATGGGTCTTAACTCTACTGAGCAAGAAGAGAAAGATAATATTCTTCGAGCTG 52
GB3LKKR01AYE55/195-429      TTCATTATGGGTCTTAACAGTACAGAGCAAGAAGAGAAAGATAATATCCTTCGTTCAG 60
GB3LKKR01A5HAT/6-242        GTTATCATGGGTCTTAACTCTACCGAGCAAGAAGAAAAAGATAATATTCTTCGAGCTG 60
contig03850/257-493         GTAATTATGGGGTTAAATGCTACAGAACAAGAAGAAAGGGATGATATATTAAGGGCCG 60
                          .........10........20........30........40........50.........

                          E  I  V  V  Q  R  D  G  L  P  S  G  R  A  L  F  K  C  D  V
GB3LKKR02JPI9I/6-242      AGATAGTAGTACAAAGGGATGGTCTTCCTTCCGGTAGAGCATTATTCAAATGCGATGTCG 120
contig03211/1-228         AGATAGTAGTACAAAGGGATGGTCTTCCTTCCGGTAGAGCATTATTCAAATGCGATGTCG 111
GB3LKKR02GT5I1/17-126     AGATGGTAGTACAAAGGGACGGTCTTTCTTCAGGTAGAGCCTTATTTAAA---------- 110
GB3LKKR01A2JGN/183-393    AGATAGTAGTACAAAGGGATGGTCTTCCTTCCGGTAGAGCATTATTCAAATGCGATGTCG 120
GB3LKKR02I0PFL/44-279     AGATAGTAGTACAAAGGGATGGTCTTCCTTCCGGTAGAGCATTATTCAAATGCGATGTCG 120
GB3LKKR02H3Y6W/1-172      -----GTAGTACAAAGGGACGGTCTTTCTTCAGGTAGAGCCTTATTTAAATGTGATGTTG 55
GB3LKKR02FQWKY/1-228      AGATAGTAGTACAAAGGGATGGTCTTCCTTCCGGTAGAGCATTATTCAAATGCGATGTCG 112
GB3LKKR01AYE55/195-429    AGATTGTAGTACAAAGGGATGGTCTTCCTTCTGGTAGAGCCTTATTTAGGTGTGATGTAG 120
GB3LKKR01A5HAT/6-242      AGATAGTAGTACAAAGGGATGGTCTTCCTTCCGGTAGAGCATTATTCAAATGCGATGTCG 120
contig03850/257-493       AGATAGTGGTTCAAAGGGATGGTCTACCATCAGGTAGAGCTCTTTTTAAATGCTCTACAG 120
                          .........70........80........90........100.......110........

                          E  R  Q  R  C  T  E  F  T  R  E  Q  R  K  Q  Y  D  G  V  Y
GB3LKKR02JPI9I/6-242      AAAGGCAAAGATGTACGGAATTTACAAGGGAACAACGTAAACAATATGATGGAGTGTATG 180
contig03211/1-228         AAAGGCAAAGATGTACGGAATTTACAAGGGAACAACGTAAACAATATGATGGAGTGTATG 171
GB3LKKR02GT5I1/17-126     ------------------------------------------------------------ 110
GB3LKKR01A2JGN/183-393    AAAGGCAAAGATGTACGGAATTTACAAGGGAACAACGTAAACAATATGATGAAGTATATG 180
GB3LKKR02I0PFL/44-279     AAAGGCAAAGATGTACGGAATTTACAAGGGAACAACGTAAACAATATGATGGAGTGTATG 180
GB3LKKR02H3Y6W/1-172      AAAGACAAAGATGTACAGAATTTACAAGAGAACAACGTAAACAATATGATGAGGTATATG 115
GB3LKKR02FQWKY/1-228      AAAGGCAAAGATGTACGGAATTTACAAGGGAACAACGTAAACAATATGATGAAGTATATG 172
GB3LKKR01AYE55/195-429    AAAGGCAAAGATGTACAGAGTTTACTAAAGAACAAACATATCAATAGCCATAGTATCA-G 179
GB3LKKR01A5HAT/6-242      AAAGGCAAAGATGTACGGAATTTACAAGGGAACAACGTAAACAATATGATGAAGTGTATT 180
contig03850/257-493       AAACTCAACGGTGTACCGAATTTACTAGAGAACAACGAAAAGAATATGATGAAGTGTATT 180
                          .........130.......140.......150.......160.......170........

                          G  S  K  L  D  E  Q  F  K  K  N  T  N  P  D  A  D  S  K
GB3LKKR02JPI9I/6-242      GTAGTAAGTTGGATGAACAATTTAAAAAGAATACTAACCCGGATGCGGATTCTAAG  237
contig03211/1-228         GTAGTAAGTTGGATGAACAATTTAAAAAGAATACTAACCCGGATGCGGATTCTAAG  228
GB3LKKR02GT5I1/17-126     --------------------------------------------------------  110
GB3LKKR01A2JGN/183-393    GTAGTAAGTTGGATGAACAATTAATAACGTA-------------------------  211
GB3LKKR02I0PFL/44-279     GTAGTAAGTTGGATGAACAATTTAAAAGAAAGATAATCCAA-ATGCTGATTCCAAG  236
GB3LKKR02H3Y6W/1-172      GTAAAAAATTGGATGAACAATTTAAGAAGAGCACTAATCCAGATGCGGATTCTAAG  172
GB3LKKR02FQWKY/1-228      GTAGTAAGTTGGATGAACAATTTAAAAAGAATACTAA-AGGTATCTATACATTTGC  228
GB3LKKR01AYE55/195-429    GTA-TAAATATAATATGATAAGGACTATAAACACATTCATAACCCTTATCAAGATC  235
GB3LKKR01A5HAT/6-242      CTGGAGTATTAGATTCTATGATGAAGAGTTCTAAAGATAATCCCTCTGCAAATAAA  237
contig03850/257-493       CTGGAGTATTAGATTCTATGATGAAGAGTTCTAAAGATAATCCCTCTGCAAATAAA  237
                          .........190.......200.......210.......220.......230.....
```

cluster375a

```
                                                    Frame -1 p =  0.013
                          F  S  I  T  P  G  I  I  Y  P  V  R  I  Q  F  V  N  A  R  D
GB3LKKR02GFL4E/48-362     TTTTCTATTACGCCGGGTATTATTTATCCGGTTCGTATTCAGTTTGTCAATGCTCGTGAT 256
GB3LKKR01A3N1M/1-255      TTTTCTATTACGCCGGGTATTATTTATCCGGTGCGTATTCAGTTTGTCAATGCTCGTGAT 256
GB3LKKR01B2UZU/181-440    ------------------------------------------------------CGTGAT 201
GB3LKKR02IIYC9/37-316     TTTTCTATTACGCCGGGTATTATTTATCCGGTGCGTATTCAGTTTGTCAATGCTCGTGAT 255
GB3LKKR02GS4KL/162-448    -------------------------AT-CGGTTCGTATTCAGTTTGTCAATGCTCGTGAT 230
GB3LKKR02GHLLT/28-339     TTTTCTGTTACGCCGGGTATTATTTACCCGGTCCGTATTCAGTTTGTCAATGCTCGTGAT 254
                          .........10........20........30........40........50.........

                          R  V  T  L  H  Q  G  I  D  V  R  L  N  P  L  G  V  P  S  F
GB3LKKR02GFL4E/48-362     CGGGTTACTTTGCATCAAGGTATTGATGTCCGTTTAAATCCTTTGGGTGTTCCGTCGTTT 196
GB3LKKR01A3N1M/1-255      CGAGTTACTTTGCATCAAGGTATTGATGTCCGTTCAAATCCTTTGGGTGTTCCGTCGTTT 196
GB3LKKR01B2UZU/181-440    CGAGTTACTTTGCATCAAGGTATTGATGTCCGTTCAAATCCTTTGGGTGTTCCGTCGTTT 141
GB3LKKR02IIYC9/37-316     CGAGTTACTTTGCATCAAGGTATTGATGTCCGTTCGAATCCTTTGGGTGTTCCGTCGTTT 195
GB3LKKR02GS4KL/162-448    CGGGTTACGCTGCATCAAGGTATTGATGTCCGTTCAAATCCCTTGGGTGTTCCATCGTTT 170
GB3LKKR02GHLLT/28-339     CGGGTTACGCTGCATCAAGGT-TTGATGTCCGTTCAAATCCTTTGGGTGTTCCATCGTTT 194
                          .........70........80........90........100.......110........

                          N  P  Y  V  L  R  L  H  R  F  W  V  P  L  Q  L  Y  H  P  E
GB3LKKR02GFL4E/48-362     AATCCTTATGTACTTCGGTTGCATCGGTTTTGGGTTCCTTTGCAGTTGTATCATCCGGAA 136
GB3LKKR01A3N1M/1-255      AATCCTTATGTACTTCGGTTGCATCGGTTTTGGGTTCCTTTGCAGTTGTATCATCCGGAA 136
GB3LKKR01B2UZU/181-440    AATCCTTATGTACTTCGGTTGCATCG-TTTTGGGTTCCTTTGCAGTTGTATCATCCGGAA 82
GB3LKKR02IIYC9/37-316     AATCCTTATGTACTTCGGTTGCATCGGTTTTGGGTCCTTTGCAGCAGTACCGGAACG-CG 136
GB3LKKR02GS4KL/162-448    AACCCTTATGTACTTCGACTGCATCGGTTTTGGGTTCCTATGCAGTTGTATCATCCTGAA 110
GB3LKKR02GHLLT/28-339     AACCCTTATGTACTTCGGCTGCATCGGTTTTGGGTTCCTT-GCAGTTGTATCATCCTGAA 135
                          .........130.......140.......150.......160.......170........

                          M  R  V  N  S  S  K  F  D  M  N  D  L  T  Y  N  F  I  P  G
GB3LKKR02GFL4E/48-362     ATGCGTGTTAATTCGTCTAAGTTCGATATGAATGATTTGACGTATAACTTTATTCCCGGT 76
GB3LKKR01A3N1M/1-255      ATGCGTGTTAATTCGTCTAAGTTCGATATGAATGATTTGACGTATAACTTTATTCCCGGT 76
GB3LKKR01B2UZU/181-440    ATGCGTGTTAATTCGTCTAAGTTCGATATGAATGATTTGACGTATAACTTTATTCCCGGT 22
GB3LKKR02IIYC9/37-316     GATACATAAAAGAAGTATAAGCACCACCACCTTTATTATCTACAACACCGGGAATAAAGT 76
GB3LKKR02GS4KL/162-448    ATGCGAGTTAATTCGTCTAAGTTTGATATGAACGATTTGACGTTTAACTTTATTCCCGGT 50
GB3LKKR02GHLLT/28-339     ATGCGAGTTAATTCGTCTAAGTTTGATATGAACGATTTGACGTTTAACTTTATTCCCAGT 76
                          .........190.......200.......210.......220.......230........

                          V  V  D  N  K  G  G  G  A  Y  T  S  F  M  Y  P  R  S  G  T
GB3LKKR02GFL4E/48-362     GTTGTAGATAATAAAGGTGGTGGTGCTTATACTTCTTTTATGTATCCGCGTTCCGGTACT 16
GB3LKKR01A3N1M/1-255      GTTGTAGATAATAAA--------------------------------------------- 16
GB3LKKR01B2UZU/181-440    GTTGTAGATAATAAAGGTGCTGGTGCTTATACTTCTTTTATGTATCCGCGTTCCGGTACT 1
GB3LKKR02IIYC9/37-316     TATACGTCAAATCATTCATATCGAACTTAGACGAATTAACA------------------- 16
GB3LKKR02GS4KL/162-448    TGTGTAGATAATTCTGGTCCAACTTCGTACACTTCTTTTATGTATCCGCGTCAACGATCG 1
GB3LKKR02GHLLT/28-339     TGTGTAGATAATAACGGTGCTGCACCGTATACTTC-TTTATGTATCCGCGTCCCGGTGTT 16
                          .........250.......260.......270.......280.......290........

                          A  A  F  F  S
GB3LKKR02GFL4E/48-362     GCTGCTTTTTTCAGT 1
GB3LKKR01A3N1M/1-255      --------------- 1
GB3LKKR01B2UZU/181-440    GCTGCTTTTTTCAGT 1
GB3LKKR02IIYC9/37-316     --------------- 1
GB3LKKR02GS4KL/162-448    GGTTCTTTATGTA-- 1
GB3LKKR02GHLLT/28-339     GCTGCATTTTTCAGT 1
                          .........310...
```

cluster406b

```
GB3LKKR02JAOTH/146-515                                                   312
GB3LKKR02JRCYP/15-455                                                    383
GB3LKKR02JUV5A/141-499                                                   300
contig01623/1-405                                                        384
GB3LKKR02F42F9/12-452                                                    382
FTSPZO101CX1PY/23-293                                                    233
                         .........10........20........30........40........50.........

GB3LKKR02JAOTH/146-515                                                   252
GB3LKKR02JRCYP/15-455                                                    323
GB3LKKR02JUV5A/141-499                                                   240
contig01623/1-405                                                        324
GB3LKKR02F42F9/12-452                                                    322
FTSPZO101CX1PY/23-293                                                    174
                         .........70........80........90........100.......110........

GB3LKKR02JAOTH/146-515                                                   192
GB3LKKR02JRCYP/15-455                                                    263
GB3LKKR02JUV5A/141-499                                                   180
contig01623/1-405                                                        264
GB3LKKR02F42F9/12-452                                                    263
FTSPZO101CX1PY/23-293                                                    114
                         .........130.......140.......150.......160.......170........

                             *  L  G  Y  S  R  S  G  F  Y  E  K  I  Q  N  R  S  F
GB3LKKR02JAOTH/146-515       TA-ATTAGGATATAGCAGATCGGGG-TTCTATGAGAAGATACAAAACAGGAGTTTT 133
GB3LKKR02JRCYP/15-455        TAAATTAGGATATAGCAGATCGGGG-TTCCATAAGAAGATACAAAACAGGAGTTT- 205
GB3LKKR02JUV5A/141-499       TAAATTAGGATATAGCAGATTCGGGGTTCTATAAGAAGATACAAAACAGGAGTTTT 120
contig01623/1-405            CAAGTTAGGGTATAGTAGGTCCGG-ATTCTATAAAAAAATACAGAACAGGAATTTT 205
GB3LKKR02F42F9/12-452        TAAATTAGGATATAGCAGAT-CGGGGTTCTATAAGAAGATACAAAACAGGAGTTTT 204
FTSPZO101CX1PY/23-293        ATCGTCTTCGGTGATGCTCGCCCAAAGGGCACACTCCAGGTATGCGCGCGTGAATG 55
                         .........190.......200.......210.......220.......230........

                                                   Frame -2 p =   0.001
                         N  I  R  E  L  A  Q  I  F  D  A  I  I  N  F  K  D  Q  D  W
GB3LKKR02JAOTH/146-515   AATATCCGGGAACTAGCTCAGATATTCGATGCGATCATCAACTTCAAAGATCAAGATTGG 75
GB3LKKR02JRCYP/15-455    AATATCCGGGAACTAGCTCAGATATTCGATGCGATCATCAACTTCAAAGATCAAGATTGG 145
GB3LKKR02JUV5A/141-499   AATATCCGGGAACTAGCTCAGATATTCGACACGATCATCAATTTCAAGGAACAGGATTGG 60
contig01623/1-405        AATATCCGAGAGCTGGCTCAGATATTCGATACGATCATCAACTTCAAAGATCAAGATTGG 145
GB3LKKR02F42F9/12-452    AATATCCGGGAGCT-CTGCAGATAT-CGAGACGATCATCAATTTCAAGGAACAGGATTGG 145
FTSPZO101CX1PY/23-293    TGTCCACGTTACCG-GTCCTTTCGTTCGACACGATCATCAACTTCAAGGATCAAGATTGG 1
                         .........250.......260.......270.......280.......290........

                         T  E  G  K  I  N  R  L  K  R  Y  R  A  M  S  L  M  E  F  N
GB3LKKR02JAOTH/146-515   ACTGAGGGTAAGATTAATAGGCTTAAAAGATATAGGGCTATGAGCCTTATGGAGTTCAAT 18
GB3LKKR02JRCYP/15-455    ACTGAGGGTAAGATTAATAGGCTTAAAAGATATAGGGCTATGAGCCTTATGGAGTTCAAT 86
GB3LKKR02JUV5A/141-499   GCGGAGAGTAAGATAGATAGGCTTAAAAGATATAGAGCCATAAGCCTTATGGAGTTCAAT 2
contig01623/1-405        ACTGAGGGTAAGATTAATAGGCTTAAAAGATATAGGGCTATGAGCCTTATGGAGTTCAAT 86
GB3LKKR02F42F9/12-452    GCGGAGAGTAAGATAGATAGGCTTAAAAGATATAGAGCCATAAGCCTTATGGAGTTCAAT 85
FTSPZO101CX1PY/23-293    ACTGAGGGTAAGATTAATAGGCTTAAGAGGTATAGGGCTATGAGCCTTATGGAGTTCAAC 1
                         .........310.......320.......330.......340.......350........

                         K  S  Y  K  K  K  K  A  *
GB3LKKR02JAOTH/146-515   AAAAGTTATAAAAAGAAAAAGGCATGA                                  1
GB3LKKR02JRCYP/15-455    AAAAGTTATAAAAAGAAAAAGGCATGA                                  26
GB3LKKR02JUV5A/141-499   AAGAATTATAAACGAAAGAAAGCATGA                                  1
contig01623/1-405        AAAAGTTATAAAAAGAAAAAGGCATGA                                  26
GB3LKKR02F42F9/12-452    AAGAATTATAAACGAAAGAAAGCATGA                                  25
FTSPZO101CX1PY/23-293    AAAAGTTATAAAAAG-AAAAGGCATGA                                  1
                         .........370.......380.......390.......400.......410........

GB3LKKR02JAOTH/146-515                               1
GB3LKKR02JRCYP/15-455                                1
GB3LKKR02JUV5A/141-499                               1
contig01623/1-405                                    1
GB3LKKR02F42F9/12-452                                1
FTSPZO101CX1PY/23-293                                1
                         .........430.......440...
```

cluster457

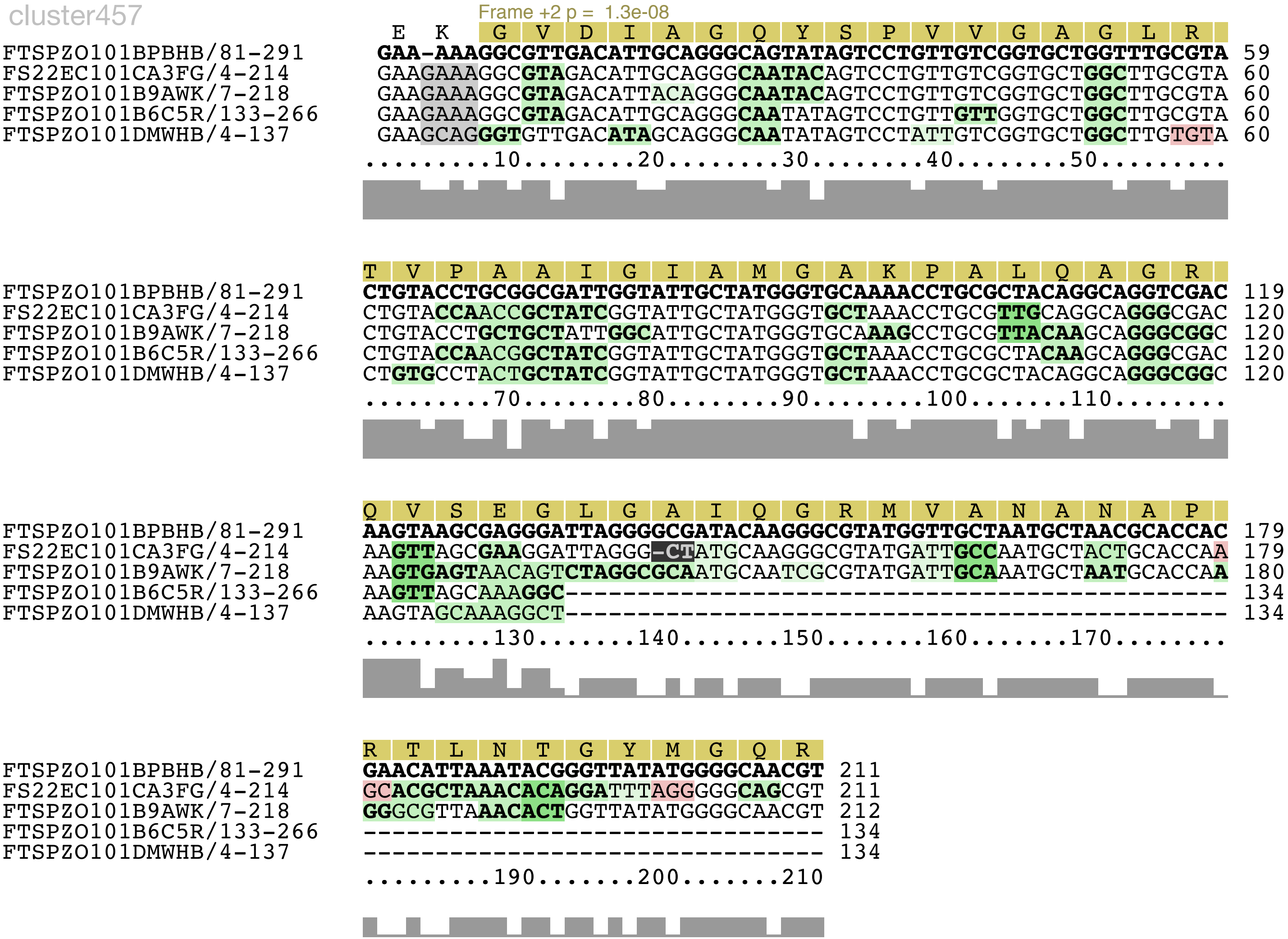
Frame +2 p = 1.3e-08
E K G V D I A G Q Y S P V V G A G L R
FTSPZO101BPHBH/81-291 GAA-AAAGGCGTTGACATTGCAGGGCAGTATAGTCCTGTTGTCGGTGCTGGTTTGCGTA 59
FS22EC101CA3FG/4-214 GAAGAAAGGCGTAGACATTGCAGGGCAATACAGTCCTGTTGTCGGTGCTGGCTTGCGTA 60
FTSPZO101B9AWK/7-218 GAAGAAAGGCGTAGACATTACAGGGCAATACAGTCCTGTTGTCGGTGCTGGCTTGCGTA 60
FTSPZO101B6C5R/133-266 GAAGAAAGGCGTAGACATTGCAGGGCAATATAGTCCTGTTGTTGGTGCTGGCTTGCGTA 60
FTSPZO101DMWHB/4-137 GAAGCAGGGTGTTGACATAGCAGGGCAATATAGTCCTATTGTCGGTGCTGGCTTGTGTA 60
.........10.........20.........30.........40.........50.........
T V P A A I G I A M G A K P A L Q A G R
FTSPZO101BPHBH/81-291 CTGTACCTGCGGCGATTGGTATTGCTATGGGTGCAAAACCTGCGCTACAGGCAGGTCGAC 119
FS22EC101CA3FG/4-214 CTGTACCAACCGCTATCGGTATTGCTATGGGTGCTAAACCTGCGTTGCAGGCAGGGCGAC 120
FTSPZO101B9AWK/7-218 CTGTACCTGCTGCTATTGGCATTGCTATGGGTGCAAAGCCTGCGTTACAAGCAGGGCGGC 120
FTSPZO101B6C5R/133-266 CTGTACCAACGGCTATCGGTATTGCTATGGGTGCTAAACCTGCGCTACAAGCAGGGCGAC 120
FTSPZO101DMWHB/4-137 CTGTGCCTACTGCTATCGGTATTGCTATGGGTGCTAAACCTGCGCTACAGGCAGGGCGGC 120
.........70.........80.........90.........100.......110........
Q V S E G L G A I Q G R M V A N A N A P
FTSPZO101BPHBH/81-291 AAGTAAGCGAGGGATTAGGGGCGATACAAGGGCGTATGGTTGCTAATGCTAACGCACCAC 179
FS22EC101CA3FG/4-214 AAGTTAGCGAAGGATTAGGG-CTATGCAAGGGCGTATGATTGCCAATGCTACTGCACCAA 179
FTSPZO101B9AWK/7-218 AAGTGAGTAACAGTCTAGGCGCAATGCAATCGCGTATGATTGCAAATGCTAATGCACCAA 180
FTSPZO101B6C5R/133-266 AAGTTAGCAAAGGC-- 134
FTSPZO101DMWHB/4-137 AAGTAGCAAAGGCT-- 134
.........130.......140.......150.......160.......170........
R T L N T G Y M G Q R
FTSPZO101BPHBH/81-291 GAACATTAAATACGGGTTATATGGGGCAACGT 211
FS22EC101CA3FG/4-214 GCACGCTAAACACAGGATTTAGGGGGCAGCGT 211
FTSPZO101B9AWK/7-218 GGGCGTTAAACACTGGTTATATGGGGCAACGT 212
FTSPZO101B6C5R/133-266 -------------------------------- 134
FTSPZO101DMWHB/4-137 -------------------------------- 134
.........190.......200.......210

cluster502

```
                                           Frame +1 p =  3.6e-07
                       D  W  L  E  T  V  Y  T  A  G  K  Y  L  D  R  P  E  T  P  V
GB3LKKR01BXXY4/1-146   GATTGGTTAGAAACAGTATATACGGCAGGAAAATACCTTGACAGACCGGAAACACCGGTA 60
contig17153/1-146      GATTGGTTGGAAACAGTATATACGGCAGGAAAGTACCTTGACAGACCGGAAACACCGGTA 60
GB3LKKR02FQIJ5/1-127   -------------------ATACGGCAGGAAAATACCTTGACAGACCGGAAACACCGGTA 41
GB3LKKR02FUJR7/1-146   GATTGGTTGGAAACAGTATATACAGCAGGTAAATACCTTGACAGACCAGAGACACCAGTG 60
                       .........10........20........30........40........50.........
```

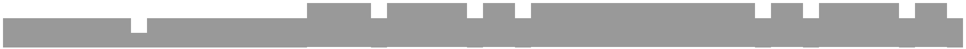

```
                       F  I  G  G  M  T  Q  Y  I  E  F  D  E  V  I  S  K  S  A  T
GB3LKKR01BXXY4/1-146   TTTATCGGCGGTATGACCCAATATATCGAGTTCGATGAAGTAATTTCAAAATCAGCAACA 120
contig17153/1-146      TTCATCGGTGGTATGACCCAATATATTGAGTTCGATGAAGTAATCTCAAAATCAGCGACA 120
GB3LKKR02FQIJ5/1-127   TTTATCGGAGGTATGACACAATATATCGAATTCGACGAAGTGATCTCAAAAAGTGCGACA 101
GB3LKKR02FUJR7/1-146   TTTATTGGAGGTATGACACAGTACATTGAATTCGACGAAGTTATCTCAAAAAGTGCGACA 120
                       .........70........80........90........100.......110........
```

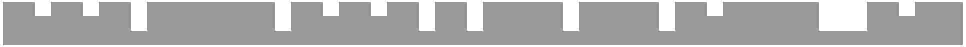

```
                       E  T  I  Y  G  S  Q  P
GB3LKKR01BXXY4/1-146   GAAACAATATACGGCTCACAACCA   146
contig17153/1-146      GATACAATATATGGCTCACAACCA   146
GB3LKKR02FQIJ5/1-127   GAAACAGCATACGGAAGTCAACCA   127
GB3LKKR02FUJR7/1-146   GAAACAGCATACGGAAGTCAACCA   146
                       .........130.......140....
```

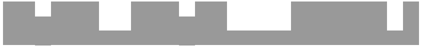

cluster532

```
                          Frame -1 p =    0.001
                          V  A  S  S  V  D  A  V  V  E  M  D  S  E  P  E  M  V  E  I
contig08608/212-370       GTCGCTTCTTCGGTGGACGCAGTGGTGGAGATGGATAGCGAGCCGGAGATGGTGGAGATA 101
GB3LKKR01BKK2R/1-158      GTCGCCTCTTCGGTGGACGCAGTGGTGGAGATGGATAGCGAGCCGGAGATGGTGGAGATA 101
GB3LKKR02HC1UT/118-244    ------------------------------GGATAGCGAGCCGGAGATGGTGGAGATA 69
GB3LKKR01CXJZO/1-159      GTAGCTTCTTCGGTGGACGCAGTGGTGGAGATGGATAGCGAGCCAGAGATGGTGGAGATA 101
GB3LKKR01BBV60/1-159      GTGGCTTCCTCTGCAGATATCGTGGTGGAGATGGATAGCGAGCCGGAGATGGTGGAGATA 101
                          .........10........20........30........40........50.........

                          E  V  E  T  G  E  F  Y  K  T  G  A  K  K  G  Q  P  K  T  G
contig08608/212-370       GAGGTCGAGACAGGAGAGTTCTATAAGACGGGAGCTAAGAAAGGTCAGCCTAAGACGG-G 41
GB3LKKR01BKK2R/1-158      GAGGTCGAGACAGGAGAGTTCTATAAGACGGGAGCTAAGAAAGGTCAGCCTAAGACG--G 41
GB3LKKR02HC1UT/118-244    GAGGTCGAGACAGGAGAGCTCTACAAGACTGGAGCCAAGAAAGGCCAACCTAAGACAG-G 9
GB3LKKR01CXJZO/1-159      GAGGTCGAGACAGGAGAGCTCTACAAGACTGGAGCCAAGAAAGGCCAACCTAAGACAGAG 41
GB3LKKR01BBV60/1-159      GAGGTCGAGACAGGAGAGCTCTACAAGACTGGAGCCAAGAAAGGCCAACCTAAGACAGAA 41
                          .........70........80........90........100.......110........

                            K  R  R  *
contig08608/212-370       AAAAAGAAGATAA                                           1
GB3LKKR01BKK2R/1-158      AAAAAGAAGATAA                                           1
GB3LKKR02HC1UT/118-244    AAAAAGAAGATAA                                           1
GB3LKKR01CXJZO/1-159      AAAAAGAAGATAA                                           1
GB3LKKR01BBV60/1-159      AAAAAGAAGATAA                                           1
                          .........130.......140.......150.......1
```

cluster540

```
                                F  K  P  C  V  L  G  W  Q  P  F  L  K  K  S  L  L  L  I  V
FTSPZO101CLSF2/1-245             TTCAAGCCGTGTGTGCTAGGGTGGCAACCCTTCTTAAAGAAAAGTCTGTTATTGATAGT 60
ER8QEOW01CIRYO/1-228             --------------CAACGAGCGAGG-AGTTGATGAATGAGCAATCAGAAATTGGAAGT 44
DBA-SLE_c2415/1-183              ----------------------------------------------------------- 0
FC8LRL301AU4EL/48-245            TACCCGCCGCATTACCAACAAGCGAGGAGTTGATGAATGAGCAATCT---GTTGATAGT 57
is_serum_c2894/36-273            TACCTGCCGCATTACCGACAAGCGAGGAGTTGATGAATGAGCAATCT---ATTGATAGT 57
cerebrospinal_rep_c831/82-323    TACCTGCCGCATTACCGACAAGCGAGGAGTTATTAGATGAGCAATCT---ATTGATAGT 57
FS22EC101AWJ2M/34-274            TACCTGCCGCATTACCAACAAGCGAGGAGTTGATGAATGAGCAATCA---ATTGATAGT 57
                                .........10........20........30........40........50.........

                                   Frame +2 p =  3.8e-04
                                E  K  V  S  K  E  D  Y  H  Q  W  L  S  N  P  V  T  K  A  M
FTSPZO101CLSF2/1-245            GGAGAAAGTGAGCAAGGAGGACTATCACCAATGGTTGAGCAATCCAGTAACGAAGGCAAT 120
ER8QEOW01CIRYO/1-228            GGAGAAGGTGAGCAAGGCGGATTACCTGCAATGGTTGAGCAATCCAGTAACCAAGGCAAT 104
DBA-SLE_c2415/1-183             --AGAAGGTGAGTATAGCGGATTACCACCAATGGTTGAGCAATCCAGTAACCAAGGCAAT 58
FC8LRL301AU4EL/48-245           GGAGAAGGTGAGTATAGCGGATTACCACCAATGGTTGAGCAATCCAGTAACCAAGGCAAT 117
is_serum_c2894/36-273           GGAGAAGGTGAGCAAGGC-GATTA-CA-CAATGGT-GGACAATCCAGTGACCAAAGCGAT 113
cerebrospinal_rep_c831/82-323   GGAGAAGGTGAGTATAGCGGATTACCACCAATGGTTGAGCAATCCAGTGACCAAGGCRAT 117
FS22EC101AWJ2M/34-274           GGAGAAGGTGAGTATAGCGGATTATCACCAATGGTTGAGCAATCCAGTAACGAAGGCGAT 117
                                .........70........80........90........100.......110........

                                   T  A  Q  L  T  N  E  L  M  S  L  N  S  L  T  S  L  P  N  P
FTSPZO101CLSF2/1-245            GACGGCGCAATTGACCAACGAGTTGATGAGTCTCAACAGCCTAACGAGCCTACCCAATCC 180
ER8QEOW01CIRYO/1-228            GACGGCGCAATTGACCAACGAGTTGATGAGTCTCAACAGCCTAACGAGCCT-CCCAACCC 163
DBA-SLE_c2415/1-183             GACAGCTCACTTGACCAACGAATGTTTGAGCCTGAACCAACTGAGCAGCCTACCGAACCC 118
FC8LRL301AU4EL/48-245           GACGGCGCAATTGACCAACGAGTTGATGAGTCTCAACAGCCTAACGAGCCTCCCCAATCC 177
is_serum_c2894/36-273           GACGGCGCAATTGACCAACGAGTTGATGAGTCTCAACAGTTTAACGAGCCTACCCAATCC 173
cerebrospinal_rep_c831/82-323   GACGGCGCAATTGACCAACGAGTTGATGAGCCTCAACAGCCTAACGAGCCTCCCCAATCC 177
FS22EC101AWJ2M/34-274           GACGGCACAATTGACCAACGAGTTGATGAGTCTCAACAGCTTAACGAGCCTACCCAACCC 177
                                .........130.......140.......150.......160.......170........

                                   L  T  L  E  K  V  H  Y  Q  L  G  K  V  Q  G  L  T  A  V  L
FTSPZO101CLSF2/1-245            GTTGACATTGGAGAAGGTGCATTACCAATTGGGCAAGGTGCAGGGATTGACGGCGGTGTT 240
ER8QEOW01CIRYO/1-228            ACAGACATTGGAGAAGGTACATTACCAATTGGGCAAGGTGCAGGGATTGACGGCGGTGCT 223
DBA-SLE_c2415/1-183             ATTGACATTGGAGGCGGTGAACTACCAACGGGGCAAGGTGTCGGGCTTGATGGCGGTCTT 178
FC8LRL301AU4EL/48-245           ATTGACATTGGAGAAGGTACA--------------------------------------- 198
is_serum_c2894/36-273           ATTGACATTGGAGAAGGTGCATTACCAATTGGGCAAGGTGCAGGGATTGACGGCGGTGCT 233
cerebrospinal_rep_c831/82-323   ATTGGCATTGGAGAAGGTACATTACCAATTGGGCAAGGTGCAGGGATTGACGGCGGTGCT 237
FS22EC101AWJ2M/34-274           ATTAACATTGGAGAAGGTGCATTACCAATTGGGGAAGGTGGTTCGATCGACTTGTTGGGC 237
                                .........190.......200.......210.......220.......230........

                                   D
FTSPZO101CLSF2/1-245            GGAT   245
ER8QEOW01CIRYO/1-228            GGAT   228
DBA-SLE_c2415/1-183             GGAT   183
FC8LRL301AU4EL/48-245           ----   198
is_serum_c2894/36-273           GGAT   238
cerebrospinal_rep_c831/82-323   GGAT   242
FS22EC101AWJ2M/34-274           GGG-   241
                                .....
```

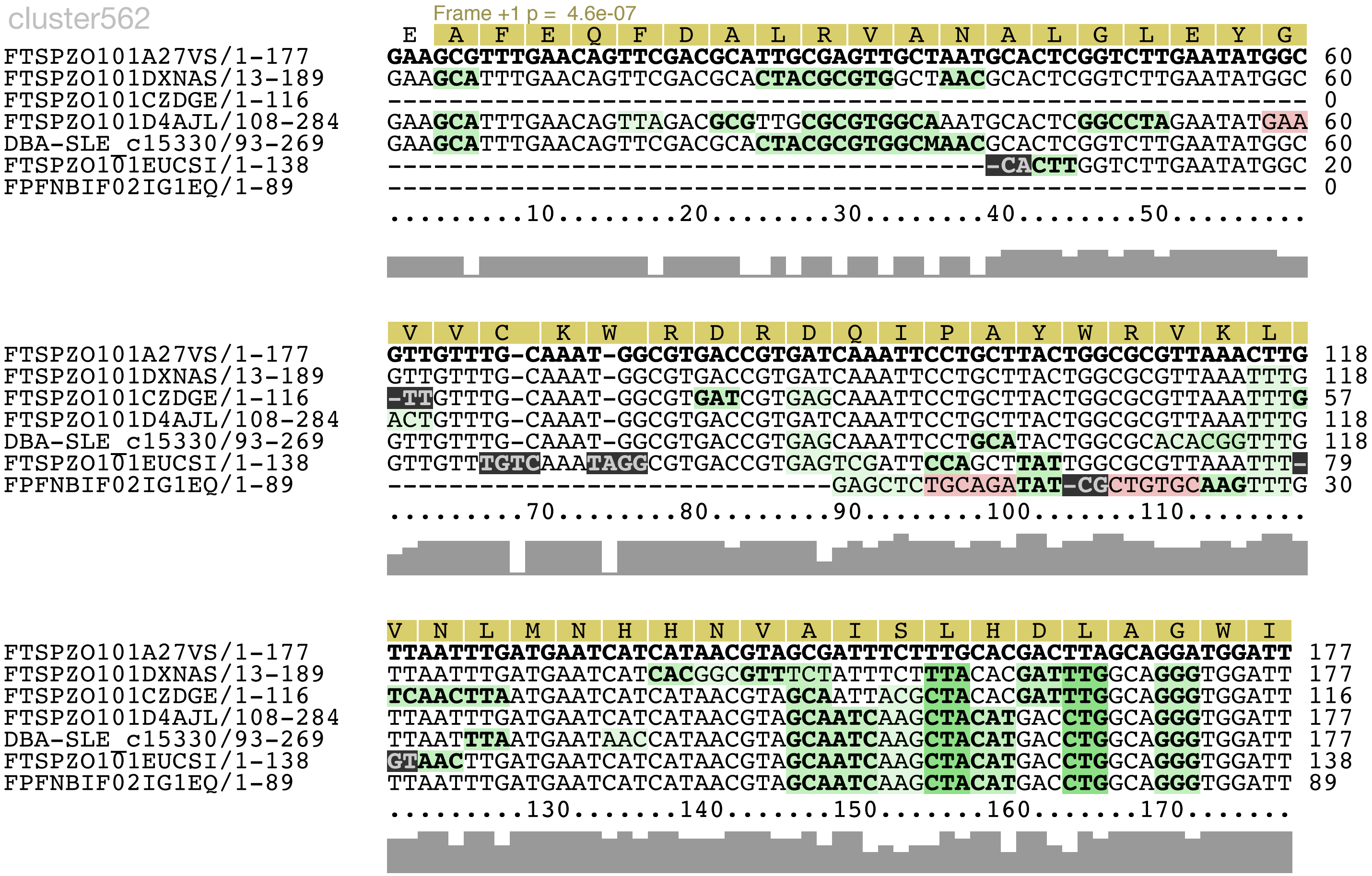
cluster562
Frame +1 p = 4.6e-07
E A F E Q F D A L R V A N A L G L E Y G
FTSPZO101A27VS/1-177 GAAGCGTTTGAACAGTTCGACGCATTGCGAGTTGCTAATGCACTCGGTCTTGAATATGGC 60
FTSPZO101DXNAS/13-189 GAAGCATTTGAACAGTTCGACGCACTACGCGTGGCTAACGCACTCGGTCTTGAATATGGC 60
FTSPZO101CZDGE/1-116 -- 0
FTSPZO101D4AJL/108-284 GAAGCATTTGAACAGTTAGACGCGTTGCGCGTGGCAAATGCACTCGGCCTAGAATATGAA 60
DBA-SLE_c15330/93-269 GAAGCATTTGAACAGTTCGACGCACTACGCGTGGCMAACGCACTCGGTCTTGAATATGGC 60
FTSPZO101EUCSI/1-138 ---------------------------------------CACTTGGTCTTGAATATGGC 20
FPFNBIF02IG1EQ/1-89 -- 0
.........10.........20.........30.........40.........50.........
V V C K W R D R D Q I P A Y W R V K L
FTSPZO101A27VS/1-177 GTTGTTTG-CAAAT-GGCGTGACCGTGATCAAATTCCTGCTTACTGGCGCGTTAAACTTG 118
FTSPZO101DXNAS/13-189 GTTGTTTG-CAAAT-GGCGTGACCGTGATCAAATTCCTGCTTACTGGCGCGTTAAATTTG 118
FTSPZO101CZDGE/1-116 -TTGTTTG-CAAAT-GGCGTGATCGTGAGCAAATTCCTGCTTACTGGCGCGTTAAATTTG 57
FTSPZO101D4AJL/108-284 ACTGTTTG-CAAAT-GGCGTGACCGTGATCAAATTCCTGCTTACTGGCGCGTTAAATTTG 118
DBA-SLE_c15330/93-269 GTTGTTTG-CAAAT-GGCGTGACCGTGAGCAAATTCCTGCATACTGGCGCACACGGTTTG 118
FTSPZO101EUCSI/1-138 GTTGTTTGTCAAATAGGCGTGACCGTGAGTCGATTCCAGCTTATTGGCGCGTTAAATTT- 79
FPFNBIF02IG1EQ/1-89 -----------------------------GAGCTCTGCAGATAT-CGCTGTGCAAGTTTG 30
.........70.........80.........90.........100.......110.........
V N L M N H H N V A I S L H D L A G W I
FTSPZO101A27VS/1-177 TTAATTTGATGAATCATCATAACGTAGCGATTTCTTTGCACGACTTAGCAGGATGGATT 177
FTSPZO101DXNAS/13-189 TTAATTTGATGAATCATCACGGCGTTTCTATTTCTTTACACGATTTGGCAGGGTGGATT 177
FTSPZO101CZDGE/1-116 TCAACTTAATGAATCATCATAACGTAGCAATTACGCTACACGATTTGGCAGGGTGGATT 116
FTSPZO101D4AJL/108-284 TTAATTTGATGAATCATCATAACGTAGCAATCAAGCTACATGACCTGGCAGGGTGGATT 177
DBA-SLE_c15330/93-269 TTAATTTAATGAATAACCATAACGTAGCAATCAAGCTACATGACCTGGCAGGGTGGATT 177
FTSPZO101EUCSI/1-138 GTAACTTGATGAATCATCATAACGTAGCAATCAAGCTACATGACCTGGCAGGGTGGATT 138
FPFNBIF02IG1EQ/1-89 TTAATTTGATGAATCATCATAACGTAGCAATCAAGCTACATGACCTGGCAGGGTGGATT 89
.........130.......140.......150.......160.......170.......

cluster565b

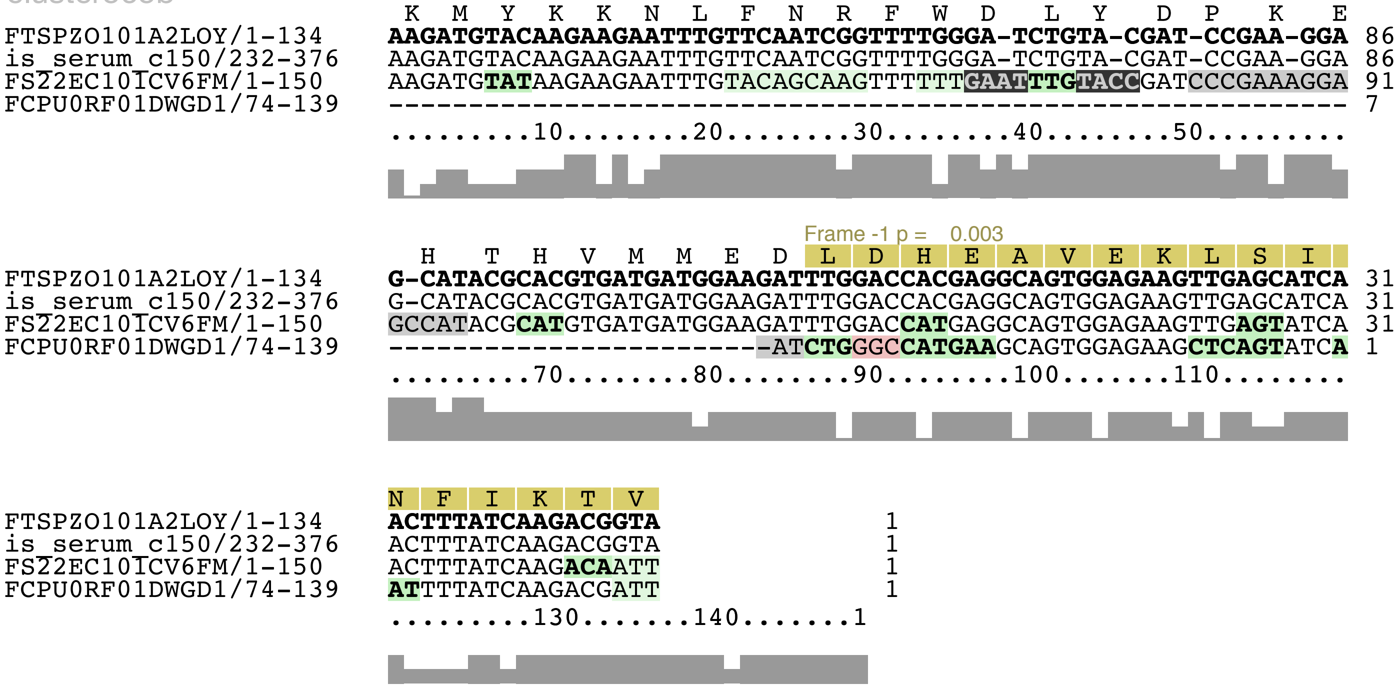

cluster589

```
                        Frame -3 p =  8.4e-04
                          E  S  Y  I  N  K  L  Y  R  L  F  L  H  V  Y  K  F  F  R
GB3LKKR01DFSJ1/79-314     GAGTCTTATATTAATAAGTTGTATAGGCTATTCTTACATGTCTATAAATTCTTCCGGA 177
GB3LKKR02FHHQ3/61-296     GAGTCTTATATTAATAAGTTGTATAGGCTATTCTTACATGTCTATAAATTCTTCCGGA 177
GB3LKKR02FG5RN/1-236      GAGTCTTATATTAATAAGTTGTATAGGCTATTCTTACATGTCTATAAATTCTTCCGGA 177
contig16113/1-235         GAATCTTATATTGGTAAGTTATATAGGTTGTTCTTATATGTCTCTAAATTCTTCCGTA 176
                        .........10........20........30........40........50.........

                        N  W  H  L  P  E  F  G  S  D  L  S  A  Y  S  G  R  I  M  F
GB3LKKR01DFSJ1/79-314   ATTGGCATTTGCCTGAGTTCGGCTCTGATCTTAGTGCTTATTCCGGTCGTATTATGTTTA 117
GB3LKKR02FHHQ3/61-296   ATTGGCATTTGCCTGCGTTCGGCTCTGATCTTAGTGCTTATTCCGGTCGTATTATGTTTA 117
GB3LKKR02FG5RN/1-236    ATTGGCATTCGCCTGAGTTCGGCTCTGATCTTAGTGCTTATTCCGGTCGTATTATGTTTA 117
contig16113/1-235       ACT-GCATTTACCTGACTTTGGTTCTGATATTAGTGCTTACTCCGGTCGTATTAATTTTA 116
                        .........70........80........90........100.......110........

                        I  L  K  T  G  I  E  Y  E  K  K  K  N  Y  E  S  L  R  D  V
GB3LKKR01DFSJ1/79-314   TTCTTAAAACGGGTATAGAGTATGAAAAGAAAAAGAATTATGAAAGTCTACGAGACGTAT 57
GB3LKKR02FHHQ3/61-296   TTCTTAAAACGGGTATAGAGTATGAAAAGAAAAAGAATTATGAAAGTCTACGAGACGTAT 57
GB3LKKR02FG5RN/1-236    TCCTTAAAACGGGTATAGAGTATGAAAAGAAAAAGAATTATGAAAGTCTACGAGACGTAT 57
contig16113/1-235       TCATTAAAACAGGTATAGAGTATGAAAAGAAAGCGGATTATGAAAGTATGCGAAATGTAT 57
                        .........130.......140.......150.......160.......170........

                        F  N  I  R  S  A  N  P  D  I  S  D  C  M  F  A  L  P  A
GB3LKKR01DFSJ1/79-314   TCAACATACGTTCCGCTAACCCGGACATATCGGATTGTATGTTTGCGTTGCCTGCG 1
GB3LKKR02FHHQ3/61-296   TCAACATACGTTCCGCTAACCCGGACATATCGGATTGTATGTTTGCGTTGCCTGCG 1
GB3LKKR02FG5RN/1-236    TCAACATACGTTCCGCTAACCCGGACATATCGGATTCTATGTTTGCGTTGCCTGCG 1
contig16113/1-235       ATGCCCTTCGTTCCCAATACTCCGAACTCTCGGATTGTATGTTTGTTTTGCCTGCG 1
                        .........190.......200.......210.......220.......230....
```

cluster612a

Frame +3 p = 0.004

```
                             L  C  A  F  K  M  S  Q  N  L  N  I  T  R  K  E  L  E  Q
GB3LKKR01DCM8C/36-229      TTATGTGCATTCAAGATGTCACAGAACTTGAACATTACAAGAAAAGAACTTGAACAGA 60
GB3LKKR01CL8OT/1-157       -----------------------------------TACAAGAAAAGAACTTGAACAGA 23
GB3LKKR01CR3JI/368-459     TTATGTGCATTTAAGATGTCACAGAACTTGAACATTACAAGAAAAGA-CTTGAACAGA 59
GB3LKKR01ESSK1/178-371     TTATGTGCATTTAAGATGTCACAGAACTTGAACATTACAAGAAAAGAACTTGAACAGA 60
GB3LKKR01E2YMD/41-145      CTATGTGCATTCAAGATGTCTCAGAACTTGAATATCACAAGGAA-GAGCTAGAACAGA 59
GB3LKKR01CLFJW/1-193       CGATGTGCATTCAAGATGTCTCAGAACTTGAATATCACAAGGGAAGAGCTAGAACAGA 60
GB3LKKR01BDX16/1-193       CTTAGTGCGTTCAAGATGTCCCAGAACTTGAACATTACAAGGAAAGAATTAGAACAGA 60
                         .........10........20........30........40........50.........

                          K  N  Q  Y  K  D  L  D  V  L  E  D  T  V  G  I  K  K  Y  I
GB3LKKR01DCM8C/36-229    AGAATCAATACAAGGATTTGGATGTACTTGAAGATACCGTTGGTATCAAGAAATACATCC 120
GB3LKKR01CL8OT/1-157     AGAATCAATACAAGGATTTGGATGTACTTGAAGATACCGTTGGTATCAAGAAATACATCC 83
GB3LKKR01CR3JI/368-459   AGAATCAATACAAGGATTTGGATGTACTTGAAG--------------------------- 92
GB3LKKR01ESSK1/178-371   AGAATCAATACAAGGATTTGGATGTACTTGAAGATACCGTTGGTATCAAGAAATACATCC 120
GB3LKKR01E2YMD/41-145    AGAATCAATACAAAGATTTGGATGTATTAGAAGATACGGTTGGTAT-------------- 105
GB3LKKR01CLFJW/1-193     AGAATCAATACAAAGATTTGGATGTATTAGAAGATACGGTTGGTATCAAGAAATACATCC 120
GB3LKKR01BDX16/1-193     AGAATCAATACAAAGATTTGGATGTATTAGAAGATACGGTTGGTATCAAGAAATACATCC 120
                         .........70........80........90........100.......110........

                         R  G  L  I  R  K  G  K  G  K  K  E  I  I  S  K  T  V  E  K
GB3LKKR01DCM8C/36-229    GAGGCTTGATTCGGAAAGGCAAAGGGAAAAAAGAAATTATCTCAAAGACAGTTGAGAAAT 180
GB3LKKR01CL8OT/1-157     GAGGCTTGATTCGGAAAGGCAAAGGGAAAAAAGAAATTATCTCAAAGACAGTTGAGAAAT 143
GB3LKKR01CR3JI/368-459   ------------------------------------------------------------ 92
GB3LKKR01ESSK1/178-371   GATGCTTGATTCGGAAAGGCAAAGAGAAAAAAGAAATTATCTCAAAGACAGTTGAGAAAT 180
GB3LKKR01E2YMD/41-145    ------------------------------------------------------------ 105
GB3LKKR01CLFJW/1-193     GAGGCTTGGTTCG-AAAGGGAAAGACAGAAAAGAAATTATCTCAAAGACAGTTGAGAAAT 179
GB3LKKR01BDX16/1-193     GAGGCTTGATTCGGAAAGGGAA-GATAGAAAAGAGGTTATTACCAAAACCGTTGAGCTAA 179
                         .........130.......140.......150.......160.......170........

                         F  G  V  P  R
GB3LKKR01DCM8C/36-229    TCGGAGTACCAAGA 194
GB3LKKR01CL8OT/1-157     TCGAAGTACCAAGA 157
GB3LKKR01CR3JI/368-459   -------------- 92
GB3LKKR01ESSK1/178-371   TTGAAGTACCAAGA 194
GB3LKKR01E2YMD/41-145    -------------- 105
GB3LKKR01CLFJW/1-193     TCGAAGTACCTAAG 193
GB3LKKR01BDX16/1-193     GGGAGCGTAAAAGG 193
                         .........190..
```

cluster787b

Frame +2 p = 0.016

```
                           H  N  Q  L  T  C  Q  P  I  T  N  I  T  Y  E  S
GB3LKKR01CCRLP/11-280           CACAACCAACTCACATGCCAACCAATCACCAACATCACCTA-CGAAAGCA 50
GB3LKKR01A9PZ6/1-317            -------AACTCACATGCCAACCAATCACCAACATCACCTA-CGAAAGCA 42
GB3LKKR01B6CHJ/4-336            TACTGCCAACTCACCTGCCAGCCAATCACCAACATCACCTA-CGAAAGCA 59
GB3LKKR01D9CHJ/12-353           TACTGTCAACTCACCTGTCAGCCAATCACCGACATCACCTA-CGAATCAA 59
contig03861/84-406              TACTGCCAACTCACCTGCCAACCAATCACCGACATCACCTA-CCAGTCAA 59
GB3LKKR02JV51K/1-304            --------ACCCGGCCAGCCAACCGAATACGCAAGC-CGTACCGCCACAC 41
                       .........10........20........30........40........50.........

I  R  T  H  P  A  T  A  I  D  L  D  A  I  D  H  E  G  H
GB3LKKR01CCRLP/11-280  TCCG---CACCCACCCAGCCACAGCAATAGACCTAGACGCCATCGACCACGAAGGCCACT 107
GB3LKKR01A9PZ6/1-317   TCCG---CACCCACCCAGCCACAGCAATAGACCTAGACGCCATCGACCACGAAGGCCACT 99
GB3LKKR01B6CHJ/4-336   TCCG---CACCCACCCAGCCTCCGCAATCGACCTCGACGCCATCGACCACGAAGGCCACT 116
GB3LKKR01D9CHJ/12-353  TCCG---CACCCACCCAGCAACCGCAATAGACCTAGACGCCATCGACCACGAAGGCCACT 116
contig03861/84-406     TCCG---CACACATCCAGCCACAGCCATCGACCTAGACGCCATCGACCATGAAGGCCACT 116
GB3LKKR02JV51K/1-304   GCCGCTTC-CACATCCAACCACAGCCATCGACCTAGACGCCATCGACCATGAAGGCCACT 100
                       .........70........80........90........100.......110........

W  V  K  A  S  A  S  K  S  T  T  N  S  T  P  Q  H  C  K  I
GB3LKKR01CCRLP/11-280  GGGTAAAAGCATCCGCTTCCAAATCGACTACAAACTCAACCCCACAACACTGTAAAATAA 167
GB3LKKR01A9PZ6/1-317   GGGTAAAAGCATCCGCTTCCAAATCGACTACAAACTCAACCCCA-AACACTGTAAAATAA 158
GB3LKKR01B6CHJ/4-336   GGGTAAAAGCATCCGCTTCCAAATCGACTACAAACTCAACCCCACAACACTGTAAAATAA 176
GB3LKKR01D9CHJ/12-353  GGGTAAAAGCATCCGCTTCCAAATCGACTACAAACTCAACCCCGCAACACTGTAAAATAA 176
contig03861/84-406     GGGTAAAAGCATCCGATTCCAAATCGACTACAAGCTCGACCCCACGACACTGTAAAATA- 175
GB3LKKR02JV51K/1-304   GGGTGAAAGCATCCGATTCCAAATCGACTACAAGCTCGACCCCA-GACACTGTAAAATA- 158
                       .........130.......140.......150.......160.......170........

K  T  A  *
GB3LKKR01CCRLP/11-280  AAACGGCATAA                                                 215
GB3LKKR01A9PZ6/1-317   AA-CGGCATAA                                                 203
GB3LKKR01B6CHJ/4-336   AAACGGCATAA                                                 222
GB3LKKR01D9CHJ/12-353  AAACGGCATAA                                                 229
contig03861/84-406     -----------                                                 210
GB3LKKR02JV51K/1-304   -----------                                                 192
                       .........190.......200.......210.......220.......230........

GB3LKKR01CCRLP/11-280                                                              270
GB3LKKR01A9PZ6/1-317                                                               263
GB3LKKR01B6CHJ/4-336                                                               282
GB3LKKR01D9CHJ/12-353                                                              288
contig03861/84-406                                                                 269
GB3LKKR02JV51K/1-304                                                               250
                       .........250.......260.......270.......280.......290........

GB3LKKR01CCRLP/11-280                                                        270
GB3LKKR01A9PZ6/1-317                                                         317
GB3LKKR01B6CHJ/4-336                                                         333
GB3LKKR01D9CHJ/12-353                                                        342
contig03861/84-406                                                           323
GB3LKKR02JV51K/1-304                                                         304
                       .........310.......320.......330.......340.......350..
```

cluster956b

Frame -1 p = 0.003

```
                        F  Q  T  G  N  S  T  Q  F  G  Y  N  S  Y  K  P  E  E  N  K
is_serum_c7126/2-160    TTTCAAACTGGTAACAGCACCCAGTTTGGATACAATTCCTACAAACCAGAAGAAAACAAA 100
FCPU0RF0ICTE83/27-185   TTCAAACTTGGTAACAGCACCCAGTTTGGGTACAACTCCTACAAGCCAGACATAAACAAC 100
is_serum_c1111/1-136    TTTCAAACTGGTAACAGCACCCAGTTTGGCTACAACTCATACAATCCACAAACAACCAAA 100
is_serum_c3480/1-159    TTCAAACTGGTAACAGCACCCAGATTTGGGTACAACTCCTACAAGCCAGACACATCCAAC 100
                        .........10........20........30........40........50.........

                        I  K  Q  A  N  A  A  Y  W  K  A  L  T  Q  Q  N  D  Q  A  T
is_serum_c7126/2-160    ATAAAACAAGCAAATGCAGCATACTGGAAGGCACTAACACAACAAAACGACCAAGCAACA 40
FCPU0RF0ICTE83/27-185   ATAAAGCAAGCAAATGAAGCATACTGGACAGCACTCACAAAAAGCAACGACCAGGCAACA 40
is_serum_c1111/1-136    ATACAAAATGCAAATAACGCATACTGGACAGCACTCACAAAACAAAACGACCAAGCAACA 40
is_serum_c3480/1-159    ATAAAGCAAGCAAATGAAGCATACTGGACAGCACTCACAAAAAGCAACGACCAGGCAACA 40
                        .........70........80........90........100.......110........

                        Q  I  G  Q  A  R  A  Q  Q  F  E  Y  H
is_serum_c7126/2-160    CAAATAGGACAAGCTCGTGCCCAACAATTTGAATACCAC 1
FCPU0RF0ICTE83/27-185   CAAATAGGCCAAGCTCGTGCCAAGCAATTTGAATACCAC 1
is_serum_c1111/1-136    CAAATAGGACAAGCCC----------------------- 1
is_serum_c3480/1-159    CAAATAGGCCAAGCTCGTGCCAAGCAATTTGAATACCAC 1
                        .........130.......140.......150.......
```

cluster1057

```
                        Frame +1 p =  3.4e-06
                         R  Y  Q  S  F  N  G  W  L  D  R  L  Q  Q  M  A  Q  T  G  F
FTSPZO101CXZAB/1-265     AGATATCAATCGTTTAATGGTTGGCTAGACCGTCTGCAACAAATGGCACAGACAGGCTTT 60
FTSPZO101C1IV6/1-308     --------ATCGTTTAATGGTTGGTTAGACCGTTTACAGCAAATGGCACAGACAGGCTTT 52
FTSPZO101BQG61/1-318     GCTAATCAGTCTTTCAATGGATGGTTAGACCGTTTACAACAAATGGCACAGACAGGCTTT 60
FTSPZO101D4HEW/1-224     AGCTCTGCAGATATCAATGGTTGGTTAGACCGTCTGCAACAAATGGCACAGACGGGCTTT 60
FS22EC101BJEV2/1-317     GCTAATCAGTCGTTCAATGGTTGGTTAGACCGTTTACAACAAATGGCACAGACTGGCTTT 60
FSTRRC101DG34E/1-94      ------------------------------------------------------------ 0
FTSPZO101A9YRG/1-105     ------------------------------------------------------------ 0
FS22EC101BSKRG/1-127     ------------------------------------------------------------ 0
                         .........10.......20.......30.......40.......50.........

                         N  A  Q  S  N  L  S  Q  A  Q  N  N  L  T  T  G  R  T  N  A
FTSPZO101CXZAB/1-265     AACGCTCAATCTAACTTATCTCAAGCTCAAAATAATCTTACGACAGGCCGCACAAATGCG 120
FTSPZO101C1IV6/1-308     AACGCTCAAACAAACTTGTCGCAAGCT-CAAACAACCTAACCACAGGCCGTACAAATGCG 111
FTSPZO101BQG61/1-318     AATGCTCAATCTAACTTGTCTCAAGCTCAAAATAACCTTACGGCAGGGCGTACAAATGCG 120
FTSPZO101D4HEW/1-224     AATGCTCAATCTAACTTATCACAAGCTCAAAATAACCTTACGGCAGGTCGCACAAATGCG 120
FS22EC101BJEV2/1-317     AATGCTCAATCTAACTTGTCGCAAGCTCAAAATAACCTTACGGCAGGTCGCACAAATGCG 120
FSTRRC101DG34E/1-94      ------------------------------------------------------------ 0
FTSPZO101A9YRG/1-105     ------------------------------------------------------------ 0
FS22EC101BSKRG/1-127     ------------------------------------------------------------ 0
                         .........70.......80.......90.......100......110........

                         L  Q  Y  G  D  T  S  A  L  D  V  G  M  G  K  D  V  L  G  I
FTSPZO101CXZAB/1-265     CTACAGTATGGCGATACAAGTGCGCTTGATGTTGGCATGGGCAAAGACGTTCTAGGTATC 180
FTSPZO101C1IV6/1-308     CTACAGTATGGCGATACAAGTGCGTTAGATGTTGGCTTGGGTAAAGATGTTTTAGGCATC 171
FTSPZO101BQG61/1-318     TTACAGTATGGTGATACAAGTGCGCTTGATGTTGGCATGGGCAAGGACGTTTTAGGCATT 180
FTSPZO101D4HEW/1-224     CTACAGTATGGCGATACAAGTGCGCTTGATGTT--------------------------- 153
FS22EC101BJEV2/1-317     CTACAGTATGGTGATACAAGTGCGCTTGATGTTGGCATGGGTAAAGACGTTTTAGGCATC 180
FSTRRC101DG34E/1-94      ------------------------------------------------------------ 0
FTSPZO101A9YRG/1-105     ------------------------------------------------------------ 0
FS22EC101BSKRG/1-127     ------------------------------------------------------------ 0
                         .........130......140......150......160......170........

                         N  Q  Q  R  G  D  V  G  I  N  R  G  Q  D  M  A  N  L  A  L
FTSPZO101CXZAB/1-265     AATCAACAACGTGGCGACGTTGGTATTAATCGCGGTCAAGACATGGCAAACTTAGCTCTT 240
FTSPZO101C1IV6/1-308     AATCAACAACGTGGTGACATTGGTATAAATCGCGGTCAAGACATGGCAAACTTAGCTCTT 231
FTSPZO101BQG61/1-318     AATCAACAGCGTGGTGACATTGGTATTAATCGTGGTCAAGACATGGCAAACTTAGCTTTA 240
FTSPZO101D4HEW/1-224     ------------------------------------------------------------ 153
FS22EC101BJEV2/1-317     AATCAACAACGTGGTGACATTGGTATAAATCGTGGTCAAGACATGGCAAACTTAGCTCTA 240
FSTRRC101DG34E/1-94      ------------------------------------------ATGGCAAACTTAGCTCTA 18
FTSPZO101A9YRG/1-105     --------------------------------CGGTCAAGACATGGC-AACTTAGCTCTA 27
FS22EC101BSKRG/1-127     -----------TGGTGACATTGGTATTAATCGTGGTCAAGACATGGCAAACTTAGCTTTA 49
                         .........190......200......210......220......230........

                         G  R  G  T  I  G  A  N
FTSPZO101CXZAB/1-265     GGTCGTGGTACGATTGGTGCTAAT                                     265
FTSPZO101C1IV6/1-308     GGTCGTGGTACGATTGGTGCTAAT                                     290
FTSPZO101BQG61/1-318     GGTCGTGGTACGATTGGTGCTAAT                                     300
FTSPZO101D4HEW/1-224     ------GGCACGATATCTGCAGAG                                     206
FS22EC101BJEV2/1-317     GGTCGTGGTACGATTGGTGCTAAT                                     299
FSTRRC101DG34E/1-94      GGTCGTGGTACGATTGGTGCTAAT                                     78
FTSPZO101A9YRG/1-105     GGTCGTGGTACGATTGGTGCTAAT                                     87
FS22EC101BSKRG/1-127     GGTCGTGGTACGATTGGTGCTAAT                                     109
                         .........250......260......270......280......290........

FTSPZO101CXZAB/1-265                       265
FTSPZO101C1IV6/1-308                       308
FTSPZO101BQG61/1-318                       318
FTSPZO101D4HEW/1-224                       224
FS22EC101BJEV2/1-317                       317
FSTRRC101DG34E/1-94                        94
FTSPZO101A9YRG/1-105                       105
FS22EC101BSKRG/1-127                       127
                         .........310......
```

cluster1073

Frame -3 p = 1.7e-10

```
                          K  T  P  E  L  S  K  A  K  K  A  L  D  A  Y  L  K
contig05414/1-269     AAGACTCCGGAGCTTTCAAAAGCAAAAAAGGCATTGGATGCTTATCTTAAA 219
GB3LKKR01DOS1W/1-272  ----ATCCAGACCTCACAAAGGCAAAGAAAGCTTTGGACGCTTATCTCAAA 213
contig07331/1-285     AAGACTCCAGAACTCACAAAGGCAAAGAAAGCTTTGGACGCTTATCTCAAA 226
GB3LKKR01CLC55/1-255  AAGACTCCAGAACTCACAAAGGCAAAGAAAGCTTTGGACGCTTATCTCAAA 226
GB3LKKR01A150I/1-276  AAGACTCCGGAACTCACCAAGGCAAAGAAAAGCTTTGATGCTTACCTTAAA 226
             .........10.........20.........30.........40.........50.........

                      E  N  K  L  D  P  Q  K  D  W  T  K  D  K  K  H  G  K  K  V
contig05414/1-269     GAAAACAAGTTGGACCCTCAAAAGGATTGGACCAAAGACAAGAAACACGGTAAGAAGGTT 159
GB3LKKR01DOS1W/1-272  GAGAACAAGTTGGACCCAACTAAAGATTGGACCAAGGACAAGAAACATGGTAAGAAGATT 153
contig07331/1-285     GAGAACAAGTTGGACCCAACTAAAGATTGGACCAAGGACAAGAAACATGGTAAGAAGATT 166
GB3LKKR01CLC55/1-255  GAGAACAAGTTGGACCCAACTAAAGATTGGACCAAGGACAAGAAACATGGTAAGAAGATT 166
GB3LKKR01A150I/1-276  GAGAACAAGTTGGACCCGACTAAGGATTGGACCAAGGACAAGAAACATGGTAAGAAGGTT 166
                      .........70.........80.........90.........100.......110........

                      T  E  L  V  N  K  L  N  K  E  R  D  K  V  A  A  E  Y  P  E
contig05414/1-269     ACCGAACTTGTTAACAAGCTCAACAAGGAACGGGATAAAGTCGCTGCTGAATATCCTGAA 99
GB3LKKR01DOS1W/1-272  ACCGAACTTGTTAACAAGCTGAACAAGGAAAGAGACAAAGTTGCTGCTGCCTATCCGGAA 93
contig07331/1-285     ACCGAACTTGTTAACAAGCTGAACAAGGAAAGAGACAAAGTTGCTGCTGCCTATCCGGAA 106
GB3LKKR01CLC55/1-255  ACCGAACTTGTTAACAAGCTGAACAAGGAAAGAGACAAAGTTGCTGCTGCCTATCCGGAA 106
GB3LKKR01A150I/1-276  ACAGAACTTGTAAATAAGCTCAACAAAGAACGGGACAAGGTAGCTGCTGCTTACCCAGAA 106
                      .........130.......140.......150.......160.......170........

                      K  D  L  K  N  E  A  K  L  V  K  M  K  E  K  K  N  A  E  K
contig05414/1-269     AAGGATTTGAAGAATGAGGCTAAACTCGTTAAGATGAAAGAGAAGAAGAATGCCGAAAAG 39
GB3LKKR01DOS1W/1-272  AAGGATGCCGACAACAACAAGAAGTTGGTAAAACTCAAAGAGAAGAAGGATAAAGAAAAA 33
contig07331/1-285     AAGGATGCCGACAACAACAAGAAGTTGGTAAAACTCAAAGAGAAGAAGGATAAAGAAAAA 46
GB3LKKR01CLC55/1-255  AAGGATGCCGACAACAACAAGAAGTTGGTAAAACTCAAAGAGAAGAAGGATAAAGAAAAA 46
GB3LKKR01A150I/1-276  GGTGACAAAGAGAATACCAAAAAATTGGTAAAACTCAGTAAAGAAAAAGGCAAGAAAGAG 46
                      .........190.......200.......210.......220.......230........

                      T  E  K  K        K        E  K  K  E  K  L  L
contig05414/1-269     ACTGAAAAGAAA---------AAAGAGAAGAAGGAAAAGCTTCTT 1
GB3LKKR01DOS1W/1-272  TCCGGCAAGAAAGAAGACAAGAAGGAGAAGAAAGAAAAGAAATCT 1
contig07331/1-285     TCCGGCAAGAAAGAAGACAAGAAGGAGAAGAAAGAAAAGAAATCT 1
GB3LKKR01CLC55/1-255  TCCGGCAAGGAAGAT------------------------------ 1
GB3LKKR01A150I/1-276  GAATCTGAAACC---------AAAGAGAAGAAGGAAAAGAAATCT 1
                      .........250.......260.......270.......280...
```

cluster1085

```
                                                                                  Frame +2 p =   0.006
                                                                   *  T  H  K  A  A
FTSPZO101A5XSA/49-223                                              TGAACACACAAAGCGGC 60
ER8QEOW01BCS2I/1-68                                                ----------------- 0
FTSPZO101A2ZKG/1-94                                                ----------------- 0
FCPU0RF01CACI4/1-115                                               --------------TGC 3
FTSPZO101C8F3X/19-176                                              CGAACGCACAAAGCGGC 60
FS22EC101A98SE/1-175                                               TGAACACATAAAGCGGC 60
FSTRRC101CPTN7/37-211                                              TGAACGCACAAAGCGGC 60
                       .........10.........20.........30.........40.........50.........

                        N  V  S  Q  F  T  R  A  E  M  A  A  T  D  S  G  L  D  V  Q
FTSPZO101A5XSA/49-223  CAACGTCTCGCAGTTTACACGCGCAGAAATGGCAGCAACAGACAGTGGCCTTGATGTGCA 120
ER8QEOW01BCS2I/1-68    ----------------------------------------------GCCTTGATGTGCA 13
FTSPZO101A2ZKG/1-94    -------------------CGCGTAG-AATGGCAGCAACAGACAGTGGCCTT-ATGTGCA 39
FCPU0RF01CACI4/1-115   AAACGTCTCGCAG---ACACGCGCAGAAATGGCCACAACAGATAGCGGCCTTGATGTTCG 60
FTSPZO101C8F3X/19-176  CAATGTCTCGCAGTTCACACGCGCAGAAATGGCAGCAACAGATAGTGGTCTTGATGTGCA 120
FS22EC101A98SE/1-175   AAACGTCTCGCAATTCACACGCGCAGAGATGGCAGCAACAGACAGCGGCCTTGATGTGCG 120
FSTRRC101CPTN7/37-211  CAATGTCTCGCAGTTCACACGCGCAGAGATGGCAGCGACAGATAGTGGTCTTGATGTGCA 120
                       .........70.........80.........90.........100.......110........

                        I  E  W  I  S  T  N  D  G  R  V  R  D  S  H  R  S  T
FTSPZO101A5XSA/49-223  AATCGAATGGATTAGCACGAATGACGGCAGGGTTAGAGACTCACACAGAAGCACA 175
ER8QEOW01BCS2I/1-68    AATCGAATGGATTAGCACGAATGACGGCAGGGTTAGAGACTCACACAGAAGCACA 68
FTSPZO101A2ZKG/1-94    AATCGAATGGATTAGCACGAATGATGGCAGGGTTAGAGACTCGCACCGAAGCGTC 94
FCPU0RF01CACI4/1-115   CATTGAGTGGATTAGCACAAATGATAGTCGGGTTAGAGACTCACACAGAAGCACA 115
FTSPZO101C8F3X/19-176  AATCGAATGGATTAGCACGAATGACGGCAGGGTTTGAT----------------- 158
FS22EC101A98SE/1-175   AATCGAGTGGATTAGCACGAATGATGGCAGGGTCAGAGACTCACATCGAGCAGTC 175
FSTRRC101CPTN7/37-211  AATCGAGTGGATTAGCACGAATGATGGCAGGGTCAGAGACCCACATCGCAATGTA 175
                       .........130.......140.......150.......160.......170...
```

cluster1217

```
                              Frame -3 p =  7.7e-10
                             *  S  T  F  G  G  Q  F  K  T  Y  K  M  T  N
GB3LKKR01DTQ2E/16-280                      TAGTCT-ACTTTTGGTGGTCAGTTCAAGACTTACAAGATGACTAAC 207
GB3LKKR01DOG04/1-117                       ---------------------------------------------- 58
GB3LKKR01AL7BN/1-265                       TAGTCT-ACTTTTGGTGGTCAGTTCAAGACTTACAAGATGACTAAC 207
GB3LKKR02FUFQQ/1-266                       TAGTCTTACTTTTGGTGGTCAGTTCAAGACTTACAAGATGACTAAC 208
contig04236/16-280                         -GGCTTTGGTTTTGGTGGTCAGTTTGTAACATACAAGATGACAAAT 207
GB3LKKR02GUTL4/19-199                      TAACTTGACTTTCGGTGGTCAGTTCAAGACTTATAAGATGACTAAT 182
                             .........10........20........30........40........50.........

                              G  I  E  L  T  L  K  Y  F  P  L  Y  D  D  T  T  Y  N  R  M
GB3LKKR01DTQ2E/16-280        GGTATCGAACTTACTCTGAAATATTTCCCATTGTATGATGATACCACTTATAACCGTATG 147
GB3LKKR01DOG04/1-117         ------------------------------------------------------------ 1
GB3LKKR01AL7BN/1-265         GGTATCGAACTTACTCTGAAGTATTTCCCATTGTATGATGATACTACTTATAATCGTATG 147
GB3LKKR02FUFQQ/1-266         GGTATCGAACTTACTCTGAAGTATTTCCCATTGTATGATGATACTACTTATAATCGTATG 148
contig04236/16-280           GGCATCGAGTTGACATTGAAACATTTCCCGTTGTATGATGATACTACTTATAATCGTTTG 147
GB3LKKR02GUTL4/19-199        GGTATCGAATTGACGCTGAAATACTTCCCATTTCACCCAGGATGTATACAGTATAACCGT 148
                             .........70........80........90........100.......110........

                              L  H  P  I  T  L  K  P  L  E  S  Y  R  L  T  F  L  D  L  G
GB3LKKR01DTQ2E/16-280        TTGCATCCTATCACACTGAAACCTCTGGAATCATATCGTTTGACATTCCTTGATCTGGGT 87
GB3LKKR01DOG04/1-117         ------------------------------TCATATCGTATGACATTCCTTGATCTGGGT 1
GB3LKKR01AL7BN/1-265         CTGCATCCGATCACACTGAAACCTCTGGAATCATATCGTATGACATTCCTTGATCTGGGT 87
GB3LKKR02FUFQQ/1-266         CTGCATCCGATCACACTGAAACCTCTGGAATCATATCGTATGACATTCCTTGATCTGGGT 88
contig04236/16-280           TTACACCCGGTATCTGGTAAACCACTGGAATCTTATAGAATGACATTCTTGGATCTTGGT 87
GB3LKKR02GUTL4/19-199        CCTTACCATTAGATCTCAGAGTATCTTTAGATTTAGCATATCCAGACGGAGCAACAGCAC 88
                             .........130.......140.......150.......160.......170........

                              R  R  D  G  E  A  N  I  V  K  V  V  R  K  D  R  E  F  V  T
GB3LKKR01DTQ2E/16-280        AGACGTGATGGTGAAGCTAACATTGTTAAGGTAGTTCGTAAAGATCGTGAATT-CGTTAC 27
GB3LKKR01DOG04/1-117         AGACGTGATGGTGAAGCTAACATTGTTAAGGTAGTTCGTAAAGATCGTGAATTCGGTTAC 1
GB3LKKR01AL7BN/1-265         AGACGTGATGGTGAAGCTAACATCGTAAAAGTAGTTCGTAAAGACCGTGA-GTTTGTAAC 27
GB3LKKR02FUFQQ/1-266         AGACGTGATGGTGAAGCTAACATCGTAAAAGTAGTTCGTAAAGACCGTGA-GTTTGTAAC 28
contig04236/16-280           AGACGTGATGGTCAAGCTAATATCGTTAAGGTTGTTCGTAAGGATCGTGAGAT-GGTTAT 27
GB3LKKR02GUTL4/19-199        C----------------------------------------------------------- 28
                             .........190.......200.......210.......220.......230........

                              W  Y  T  G  G  A  V  A
GB3LKKR01DTQ2E/16-280        TTGGTATACTGGTGGTGCTGTTGCT   1
GB3LKKR01DOG04/1-117         TTGGTATACTGGTGGTGCTGTTGCT   1
GB3LKKR01AL7BN/1-265         TTGGTACACTGGTGGTGCTGTTGCT   1
GB3LKKR02FUFQQ/1-266         TTGGTACACTGGTGGTGCTGTTGCT   1
contig04236/16-280           CTGGAATACTTCAGGTTCTGTAGCT   1
GB3LKKR02GUTL4/19-199        -------------------------   1
                             .........250.......260.....
```

cluster1423

```
                      Frame +1 p =  1.0e-04
                       Q  R  V  Q  Y  I  S  P  L  E  D  G  S  Y  K  V  T  L  V  S
DBA-SLE_c4340/1-188    CAGAGGGTGCAGTATATTAGTCCTTTAGAAGATGGAAGCTACAAAGTTACATTAGTCAGT 60
FTSPZO101BASEK/1-188   CAGAGGGGAAGGTATATTAGTCCTTTAGAAGATGGAAGCTACAAAGTTACATTAGTCAGT 60
DBA-SLE_c13402/1-183   CAGAGGGTGCAGTATATTAGTCCTATTGACAAGGGCAGCTATAAAGTTACATTGGTTAGC 60
FTSPZO101CIWNM/1-146   ---------------------------------------CTGCAGATATCATTGGTTAGC 21
FTSPZO101DPI37/1-130   ---------------------------------------------------TTGGTTAGC 9
                       .........10.........20.........30.........40.........50.........
```

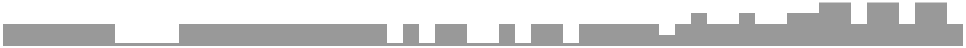

```
                       G  W  E  M  K  L  T  D  K  L  Y  S  R  E  K  L  I  G  L  W
DBA-SLE_c4340/1-188    GGGTGGGAAATGAAGCTTACAGATAAGTTGTACAGTAGAGAGAAGTTGATTGGGTTGTGG 120
FTSPZO101BASEK/1-188   GGGTGGGAAATGAAGCTTACAGATAAGTTGTACAGTAGAGAGAAGTTGATTGGGTTGTGG 120
DBA-SLE_c13402/1-183   GGGTGGGAAATGCCGTTGACTGATAAGTTGTACAGTAGAGAGAAGTTGATTGGACAGTGG 120
FTSPZO101CIWNM/1-146   GGGTGGGAAATGAAGTTGACGGGTGAGGTGTATAGTAGAGAGAAGTTGACTGGGTTGTGG 81
FTSPZO101DPI37/1-130   GGGTGGGAAATGCCGTTGACTGATAAGTTGTACAGTAGAGAGAAGTTGATTGGGTTGTGG 69
                       .........70.........80.........90.........100.......110........
```

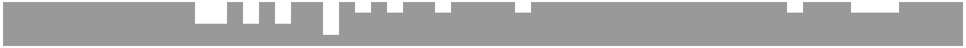

```
                       G  V  L  Q  N  K  *
DBA-SLE_c4340/1-188    GGAGTTTTGCAGAATAAATAG                                        180
FTSPZO101BASEK/1-188   GGAGTTTTGCAGAATAAATAG                                        180
DBA-SLE_c13402/1-183   CGCATGTTGCAGAATAAATA-                                        173
FTSPZO101CIWNM/1-146   AAGGGGTTGCAGAACAAATAA                                        140
FTSPZO101DPI37/1-130   CGTATATTGCAGAATAAATAG                                        122
                       .........130.......140.......150.......160.......170........
```

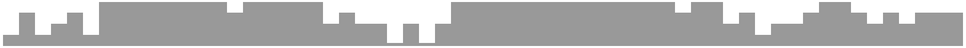

```
DBA-SLE_c4340/1-188              188
FTSPZO101BASEK/1-188             188
DBA-SLE_c13402/1-183             183
FTSPZO101CIWNM/1-146             146
FTSPZO101DPI37/1-130             130
                       .........1
```

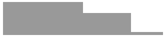

Supplementary Figure S3. Whole genome comparison of the bacteriophage HFM assembly with three metagenomic contigs from the NCBI env_nt database using Mauve[1]. The sequence of the selected env_nt contigs corresponds mostly to our assembled circular genome. Assuming the contigs are derived from closely related bacteriophages, differences in the order of the conserved blocks (in different colors) may be attributed to different cut points during the linearization of the circular genome, rearrangements, or possible misassemblies. The middle sections of the contigs are well conserved between the different contigs, but the flanking regions differ considerably. Corresponding segments in different contigs are connected with lines in the same colour.

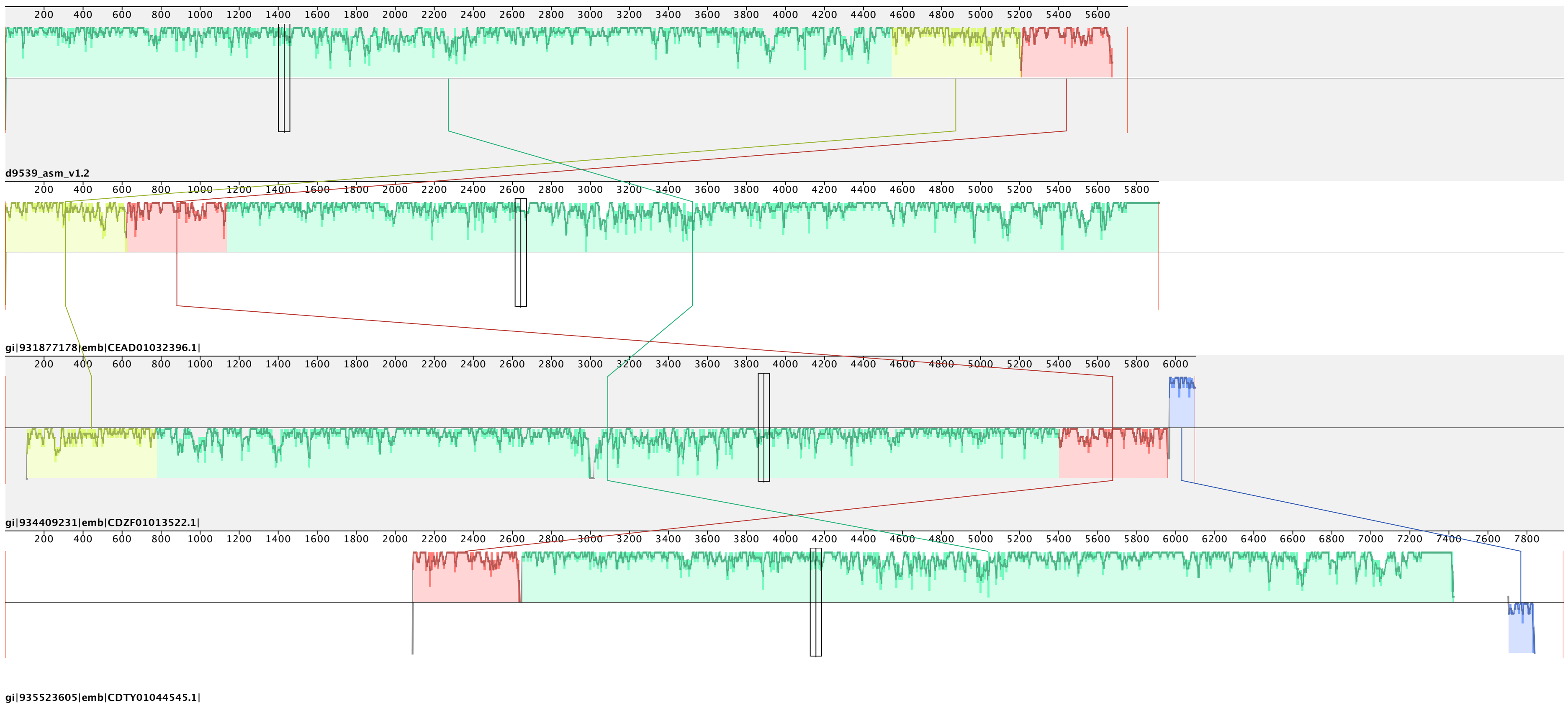
d9539_asm_v1.2
gi|931877178|emb|CEAD01032396.1|
gi|934409231|emb|CDZF01013522.1|
gi|935523605|emb|CDTY01044545.1|

Supplementary Figure S4. Neighbour-joining tree of 331 phage capsid F proteins from Uniprot, as well as bacteriophage HFM ORF6 (highlighted in gray). Tree nodes are marked with bootstrap support as percentages. The species as listed in Uniprot is shown to the right and colour coded. Note however that in many cases the listed species is the host in which the phage was found. This will happen for proteins from e.g. “Unassembled WGS sequence” entries.

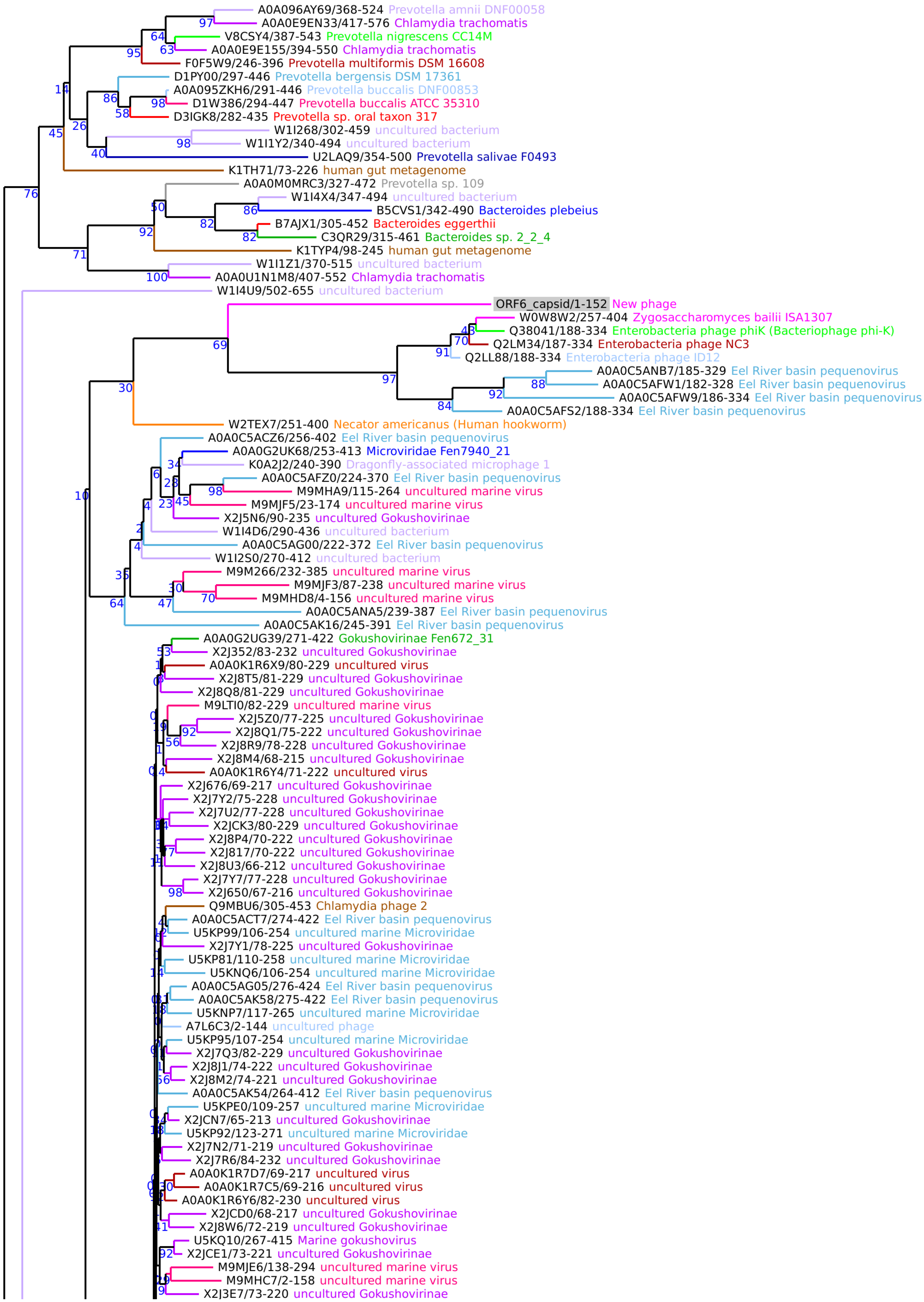

A0A096AY69/368-524 Prevotella amnii DNF00058
A0A0E9EN33/417-576 Chlamydia trachomatis
V8CSY4/387-543 Prevotella nigrescens CC14M
A0A0E9E155/394-550 Chlamydia trachomatis
F0F5W9/246-396 Prevotella multiformis DSM 16608
D1PY00/297-446 Prevotella bergensis DSM 17361
A0A095ZKH6/291-446 Prevotella buccalis DNF00853
D1W386/294-447 Prevotella buccalis ATCC 35310
D3IGK8/282-435 Prevotella sp. oral taxon 317
W1I268/302-459 uncultured bacterium
W1I1Y2/340-494 uncultured bacterium
U2LAQ9/354-500 Prevotella salivae F0493
K1TH71/73-226 human gut metagenome
A0A0M0MRC3/327-472 Prevotella sp. 109
W1I4X4/347-494 uncultured bacterium
B5CVS1/342-490 Bacteroides plebeius
B7AJX1/305-452 Bacteroides eggerthii
C3QR29/315-461 Bacteroides sp. 2_2_4
K1TYP4/98-245 human gut metagenome
W1I1Z1/370-515 uncultured bacterium
A0A0U1N1M8/407-552 Chlamydia trachomatis
W1I4U9/502-655 uncultured bacterium
ORF6_capsid/1-152 New phage
W0W8W2/257-404 Zygosaccharomyces bailii ISA1307
Q38041/188-334 Enterobacteria phage phiK (Bacteriophage phi-K)
Q2LM34/187-334 Enterobacteria phage NC3
Q2LL88/188-334 Enterobacteria phage ID12
A0A0C5ANB7/185-329 Eel River basin pequenovirus
A0A0C5AFW1/182-328 Eel River basin pequenovirus
A0A0C5AFW9/186-334 Eel River basin pequenovirus
A0A0C5AFS2/188-334 Eel River basin pequenovirus
W2TEX7/251-400 Necator americanus (Human hookworm)
A0A0C5ACZ6/256-402 Eel River basin pequenovirus
A0A0G2UK68/253-413 Microviridae Fen7940_21
K0A2J2/240-390 Dragonfly-associated microphage 1
A0A0C5AFZ0/224-370 Eel River basin pequenovirus
M9MHA9/115-264 uncultured marine virus
M9MJF5/23-174 uncultured marine virus
X2J5N6/90-235 uncultured Gokushovirinae
W1I4D6/290-436 uncultured bacterium
A0A0C5AG00/222-372 Eel River basin pequenovirus
W1I2S0/270-412 uncultured bacterium
M9M266/232-385 uncultured marine virus
M9MJF3/87-238 uncultured marine virus
M9MHD8/4-156 uncultured marine virus
A0A0C5ANA5/239-387 Eel River basin pequenovirus
A0A0C5AK16/245-391 Eel River basin pequenovirus
A0A0G2UG39/271-422 Gokushovirinae Fen672_31
X2J352/83-232 uncultured Gokushovirinae
A0A0K1R6X9/80-229 uncultured virus
X2J8T5/81-229 uncultured Gokushovirinae
X2J8Q8/81-229 uncultured Gokushovirinae
M9LTI0/82-229 uncultured marine virus
X2J5Z0/77-225 uncultured Gokushovirinae
X2J8Q1/75-222 uncultured Gokushovirinae
X2J8R9/78-228 uncultured Gokushovirinae
X2J8M4/68-215 uncultured Gokushovirinae
A0A0K1R6Y4/71-222 uncultured virus
X2J676/69-217 uncultured Gokushovirinae
X2J7Y2/75-228 uncultured Gokushovirinae
X2J7U2/77-228 uncultured Gokushovirinae
X2JCK3/80-229 uncultured Gokushovirinae
X2J8P4/70-222 uncultured Gokushovirinae
X2J817/70-222 uncultured Gokushovirinae
X2J8U3/66-212 uncultured Gokushovirinae
X2J7Y7/77-228 uncultured Gokushovirinae
X2J650/67-216 uncultured Gokushovirinae
Q9MBU6/305-453 Chlamydia phage 2
A0A0C5ACT7/274-422 Eel River basin pequenovirus
U5KP99/106-254 uncultured marine Microviridae
X2J7Y1/78-225 uncultured Gokushovirinae
U5KP81/110-258 uncultured marine Microviridae
U5KNQ6/106-254 uncultured marine Microviridae
A0A0C5AG05/276-424 Eel River basin pequenovirus
A0A0C5AK58/275-422 Eel River basin pequenovirus
U5KNP7/117-265 uncultured marine Microviridae
A7L6C3/2-144 uncultured phage
U5KP95/107-254 uncultured marine Microviridae
X2J7Q3/82-229 uncultured Gokushovirinae
X2J8J1/74-222 uncultured Gokushovirinae
X2J8M2/74-221 uncultured Gokushovirinae
A0A0C5AK54/264-412 Eel River basin pequenovirus
U5KPE0/109-257 uncultured marine Microviridae
X2JCN7/65-213 uncultured Gokushovirinae
U5KP92/123-271 uncultured marine Microviridae
X2J7N2/71-219 uncultured Gokushovirinae
X2J7R6/84-232 uncultured Gokushovirinae
A0A0K1R7D7/69-217 uncultured virus
A0A0K1R7C5/69-216 uncultured virus
A0A0K1R6Y6/82-230 uncultured virus
X2JCD0/68-217 uncultured Gokushovirinae
X2J8W6/72-219 uncultured Gokushovirinae
U5KQ10/267-415 Marine gokushovirus
X2JCE1/73-221 uncultured Gokushovirinae
M9MJE6/138-294 uncultured marine virus
M9MHC7/2-158 uncultured marine virus
X2J3E7/73-220 uncultured Gokushovirinae

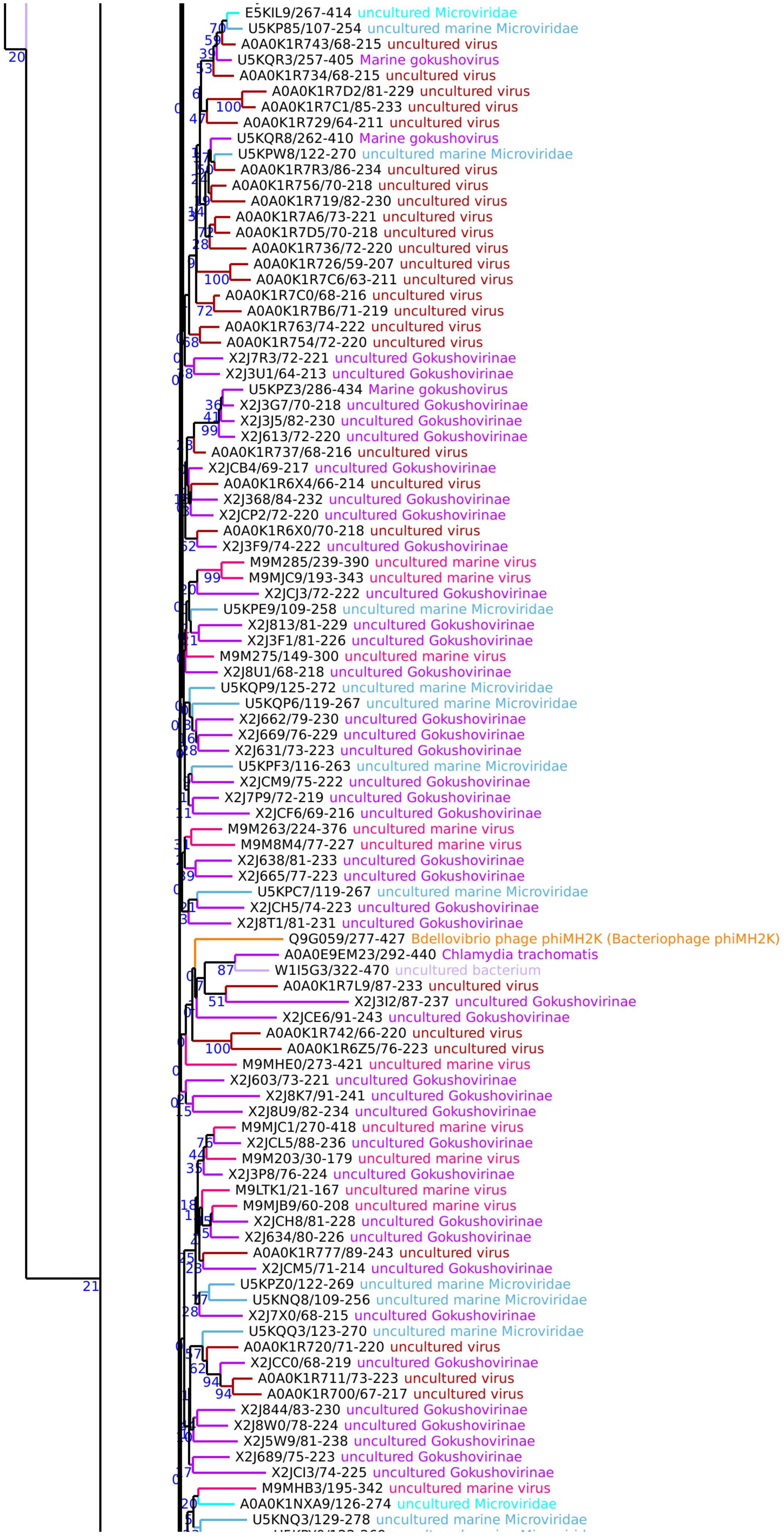
E5KIL9/267-414 uncultured Microviridae
U5KP85/107-254 uncultured marine Microviridae
A0A0K1R743/68-215 uncultured virus
U5KQR3/257-405 Marine gokushovirus
A0A0K1R734/68-215 uncultured virus
A0A0K1R7D2/81-229 uncultured virus
A0A0K1R7C1/85-233 uncultured virus
A0A0K1R729/64-211 uncultured virus
U5KQR8/262-410 Marine gokushovirus
U5KPW8/122-270 uncultured marine Microviridae
A0A0K1R7R3/86-234 uncultured virus
A0A0K1R756/70-218 uncultured virus
A0A0K1R719/82-230 uncultured virus
A0A0K1R7A6/73-221 uncultured virus
A0A0K1R7D5/70-218 uncultured virus
A0A0K1R736/72-220 uncultured virus
A0A0K1R726/59-207 uncultured virus
A0A0K1R7C6/63-211 uncultured virus
A0A0K1R7C0/68-216 uncultured virus
A0A0K1R7B6/71-219 uncultured virus
A0A0K1R763/74-222 uncultured virus
A0A0K1R754/72-220 uncultured virus
X2J7R3/72-221 uncultured Gokushovirinae
X2J3U1/64-213 uncultured Gokushovirinae
U5KPZ3/286-434 Marine gokushovirus
X2J3G7/70-218 uncultured Gokushovirinae
X2J3J5/82-230 uncultured Gokushovirinae
X2J613/72-220 uncultured Gokushovirinae
A0A0K1R737/68-216 uncultured virus
X2JCB4/69-217 uncultured Gokushovirinae
A0A0K1R6X4/66-214 uncultured virus
X2J368/84-232 uncultured Gokushovirinae
X2JCP2/72-220 uncultured Gokushovirinae
A0A0K1R6X0/70-218 uncultured virus
X2J3F9/74-222 uncultured Gokushovirinae
M9M285/239-390 uncultured marine virus
M9MJC9/193-343 uncultured marine virus
X2JCJ3/72-222 uncultured Gokushovirinae
U5KPE9/109-258 uncultured marine Microviridae
X2J813/81-229 uncultured Gokushovirinae
X2J3F1/81-226 uncultured Gokushovirinae
M9M275/149-300 uncultured marine virus
X2J8U1/68-218 uncultured Gokushovirinae
U5KQP9/125-272 uncultured marine Microviridae
U5KQP6/119-267 uncultured marine Microviridae
X2J662/79-230 uncultured Gokushovirinae
X2J669/76-229 uncultured Gokushovirinae
X2J631/73-223 uncultured Gokushovirinae
U5KPF3/116-263 uncultured marine Microviridae
X2JCM9/75-222 uncultured Gokushovirinae
X2J7P9/72-219 uncultured Gokushovirinae
X2JCF6/69-216 uncultured Gokushovirinae
M9M263/224-376 uncultured marine virus
M9M8M4/77-227 uncultured marine virus
X2J638/81-233 uncultured Gokushovirinae
X2J665/77-223 uncultured Gokushovirinae
U5KPC7/119-267 uncultured marine Microviridae
X2JCH5/74-223 uncultured Gokushovirinae
X2J8T1/81-231 uncultured Gokushovirinae
Q9G059/277-427 Bdellovibrio phage phiMH2K (Bacteriophage phiMH2K)
A0A0E9EM23/292-440 Chlamydia trachomatis
W1I5G3/322-470 uncultured bacterium
A0A0K1R7L9/87-233 uncultured virus
X2J3I2/87-237 uncultured Gokushovirinae
X2JCE6/91-243 uncultured Gokushovirinae
A0A0K1R742/66-220 uncultured virus
A0A0K1R6Z5/76-223 uncultured virus
M9MHE0/273-421 uncultured marine virus
X2J603/73-221 uncultured Gokushovirinae
X2J8K7/91-241 uncultured Gokushovirinae
X2J8U9/82-234 uncultured Gokushovirinae
M9MJC1/270-418 uncultured marine virus
X2JCL5/88-236 uncultured Gokushovirinae
M9M203/30-179 uncultured marine virus
X2J3P8/76-224 uncultured Gokushovirinae
M9LTK1/21-167 uncultured marine virus
M9MJB9/60-208 uncultured marine virus
X2JCH8/81-228 uncultured Gokushovirinae
X2J634/80-226 uncultured Gokushovirinae
A0A0K1R777/89-243 uncultured virus
X2JCM5/71-214 uncultured Gokushovirinae
U5KPZ0/122-269 uncultured marine Microviridae
U5KNQ8/109-256 uncultured marine Microviridae
X2J7X0/68-215 uncultured Gokushovirinae
U5KQQ3/123-270 uncultured marine Microviridae
A0A0K1R720/71-220 uncultured virus
X2JCC0/68-219 uncultured Gokushovirinae
A0A0K1R711/73-223 uncultured virus
A0A0K1R700/67-217 uncultured virus
X2J844/83-230 uncultured Gokushovirinae
X2J8W0/78-224 uncultured Gokushovirinae
X2J5W9/81-238 uncultured Gokushovirinae
X2J689/75-223 uncultured Gokushovirinae
X2JCI3/74-225 uncultured Gokushovirinae
M9MHB3/195-342 uncultured marine virus
A0A0K1NXA9/126-274 uncultured Microviridae
U5KNQ3/129-278 uncultured marine Microviridae

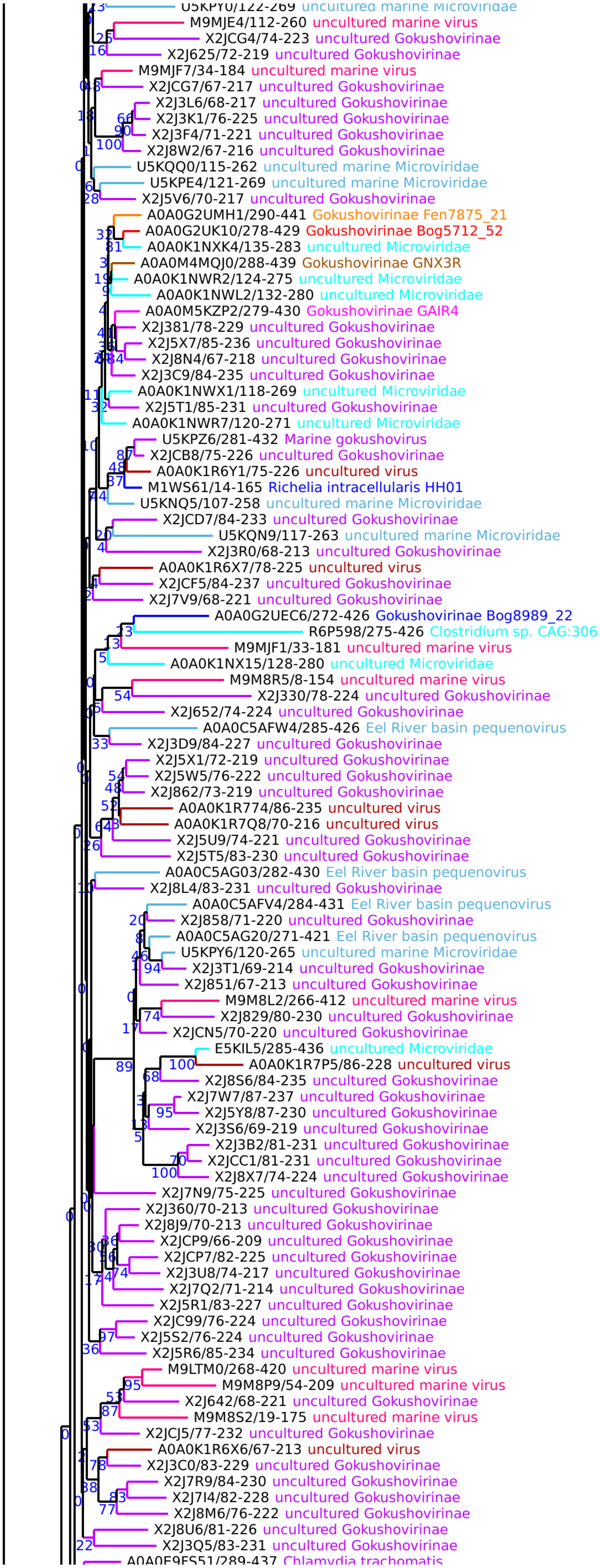

U5KPY0/122-269 uncultured marine Microviridae
M9MJE4/112-260 uncultured marine virus
X2JCG4/74-223 uncultured Gokushovirinae
X2J625/72-219 uncultured Gokushovirinae
M9MJF7/34-184 uncultured marine virus
X2JCG7/67-217 uncultured Gokushovirinae
X2J3L6/68-217 uncultured Gokushovirinae
X2J3K1/76-225 uncultured Gokushovirinae
X2J3F4/71-221 uncultured Gokushovirinae
X2J8W2/67-216 uncultured Gokushovirinae
U5KQQ0/115-262 uncultured marine Microviridae
U5KPE4/121-269 uncultured marine Microviridae
X2J5V6/70-217 uncultured Gokushovirinae
A0A0G2UMH1/290-441 Gokushovirinae Fen7875_21
A0A0G2UK10/278-429 Gokushovirinae Bog5712_52
A0A0K1NXK4/135-283 uncultured Microviridae
A0A0M4MQJ0/288-439 Gokushovirinae GNX3R
A0A0K1NWR2/124-275 uncultured Microviridae
A0A0K1NWL2/132-280 uncultured Microviridae
A0A0M5KZP2/279-430 Gokushovirinae GAIR4
X2J381/78-229 uncultured Gokushovirinae
X2J5X7/85-236 uncultured Gokushovirinae
X2J8N4/67-218 uncultured Gokushovirinae
X2J3C9/84-235 uncultured Gokushovirinae
A0A0K1NWX1/118-269 uncultured Microviridae
X2J5T1/85-231 uncultured Gokushovirinae
A0A0K1NWR7/120-271 uncultured Microviridae
U5KPZ6/281-432 Marine gokushovirus
X2JCB8/75-226 uncultured Gokushovirinae
A0A0K1R6Y1/75-226 uncultured virus
M1WS61/14-165 Richelia intracellularis HH01
U5KNQ5/107-258 uncultured marine Microviridae
X2JCD7/84-233 uncultured Gokushovirinae
U5KQN9/117-263 uncultured marine Microviridae
X2J3R0/68-213 uncultured Gokushovirinae
A0A0K1R6X7/78-225 uncultured virus
X2JCF5/84-237 uncultured Gokushovirinae
X2J7V9/68-221 uncultured Gokushovirinae
A0A0G2UEC6/272-426 Gokushovirinae Bog8989_22
R6P598/275-426 Clostridium sp. CAG:306
M9MJF1/33-181 uncultured marine virus
A0A0K1NX15/128-280 uncultured Microviridae
M9M8R5/8-154 uncultured marine virus
X2J330/78-224 uncultured Gokushovirinae
X2J652/74-224 uncultured Gokushovirinae
A0A0C5AFW4/285-426 Eel River basin pequenovirus
X2J3D9/84-227 uncultured Gokushovirinae
X2J5X1/72-219 uncultured Gokushovirinae
X2J5W5/76-222 uncultured Gokushovirinae
X2J862/73-219 uncultured Gokushovirinae
A0A0K1R774/86-235 uncultured virus
A0A0K1R7Q8/70-216 uncultured virus
X2J5U9/74-221 uncultured Gokushovirinae
X2J5T5/83-230 uncultured Gokushovirinae
A0A0C5AG03/282-430 Eel River basin pequenovirus
X2J8L4/83-231 uncultured Gokushovirinae
A0A0C5AFV4/284-431 Eel River basin pequenovirus
X2J858/71-220 uncultured Gokushovirinae
A0A0C5AG20/271-421 Eel River basin pequenovirus
U5KPY6/120-265 uncultured marine Microviridae
X2J3T1/69-214 uncultured Gokushovirinae
X2J851/67-213 uncultured Gokushovirinae
M9M8L2/266-412 uncultured marine virus
X2J829/80-230 uncultured Gokushovirinae
X2JCN5/70-220 uncultured Gokushovirinae
E5KIL5/285-436 uncultured Microviridae
A0A0K1R7P5/86-228 uncultured virus
X2J8S6/84-235 uncultured Gokushovirinae
X2J7W7/87-237 uncultured Gokushovirinae
X2J5Y8/87-230 uncultured Gokushovirinae
X2J3S6/69-219 uncultured Gokushovirinae
X2J3B2/81-231 uncultured Gokushovirinae
X2JCC1/81-231 uncultured Gokushovirinae
X2J8X7/74-224 uncultured Gokushovirinae
X2J7N9/75-225 uncultured Gokushovirinae
X2J360/70-213 uncultured Gokushovirinae
X2J8J9/70-213 uncultured Gokushovirinae
X2JCP9/66-209 uncultured Gokushovirinae
X2JCP7/82-225 uncultured Gokushovirinae
X2J3U8/74-217 uncultured Gokushovirinae
X2J7Q2/71-214 uncultured Gokushovirinae
X2J5R1/83-227 uncultured Gokushovirinae
X2JC99/76-224 uncultured Gokushovirinae
X2J5S2/76-224 uncultured Gokushovirinae
X2J5R6/85-234 uncultured Gokushovirinae
M9LTM0/268-420 uncultured marine virus
M9M8P9/54-209 uncultured marine virus
X2J642/68-221 uncultured Gokushovirinae
M9M8S2/19-175 uncultured marine virus
X2JCJ5/77-232 uncultured Gokushovirinae
A0A0K1R6X6/67-213 uncultured virus
X2J3C0/83-229 uncultured Gokushovirinae
X2J7R9/84-230 uncultured Gokushovirinae
X2J7I4/82-228 uncultured Gokushovirinae
X2J8M6/76-222 uncultured Gokushovirinae
X2J8U6/81-226 uncultured Gokushovirinae
X2J3Q5/83-231 uncultured Gokushovirinae
A0A0E9FS51/289-437 Chlamydia trachomatis

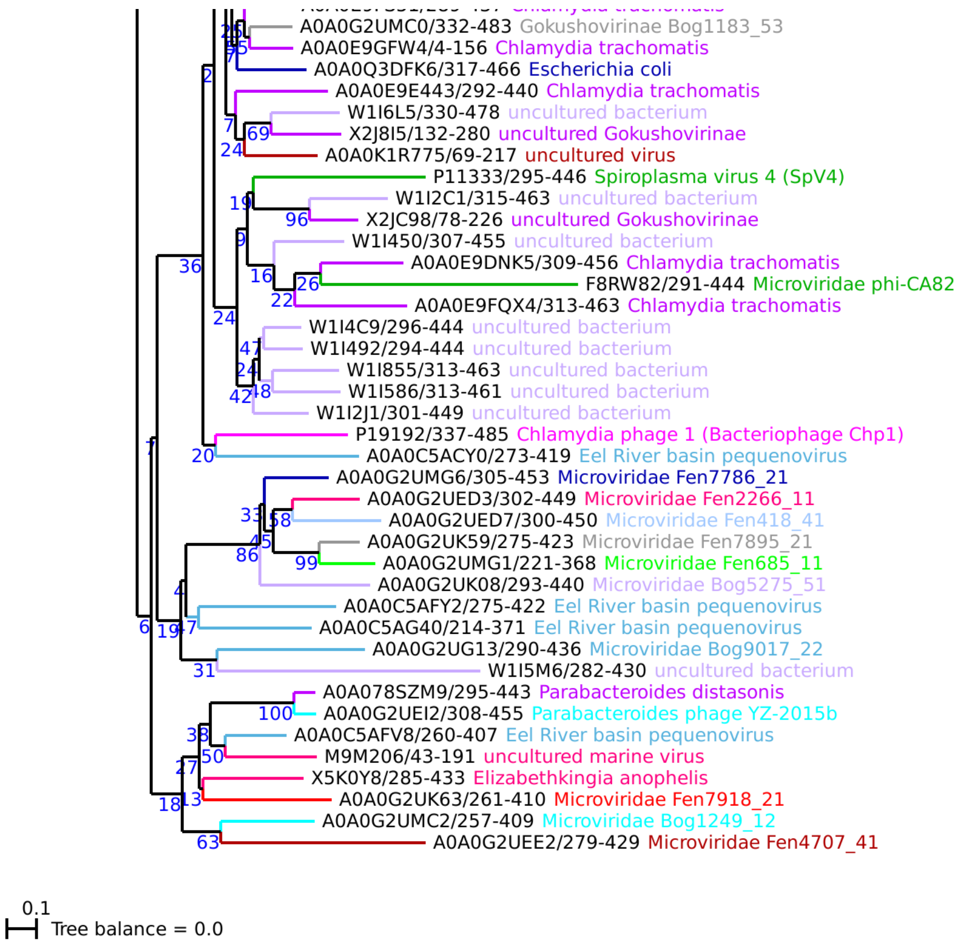
A0A0G2UMC0/332-483 Gokushovirinae Bog1183_53
A0A0E9GFW4/4-156 Chlamydia trachomatis
A0A0Q3DFK6/317-466 Escherichia coli
A0A0E9E443/292-440 Chlamydia trachomatis
W1I6L5/330-478 uncultured bacterium
X2J8I5/132-280 uncultured Gokushovirinae
A0A0K1R775/69-217 uncultured virus
P11333/295-446 Spiroplasma virus 4 (SpV4)
W1I2C1/315-463 uncultured bacterium
X2JC98/78-226 uncultured Gokushovirinae
W1I450/307-455 uncultured bacterium
A0A0E9DNK5/309-456 Chlamydia trachomatis
F8RW82/291-444 Microviridae phi-CA82
A0A0E9FQX4/313-463 Chlamydia trachomatis
W1I4C9/296-444 uncultured bacterium
W1I492/294-444 uncultured bacterium
W1I855/313-463 uncultured bacterium
W1I586/313-461 uncultured bacterium
W1I2J1/301-449 uncultured bacterium
P19192/337-485 Chlamydia phage 1 (Bacteriophage Chp1)
A0A0C5ACY0/273-419 Eel River basin pequenovirus
A0A0G2UMG6/305-453 Microviridae Fen7786_21
A0A0G2UED3/302-449 Microviridae Fen2266_11
A0A0G2UED7/300-450 Microviridae Fen418_41
A0A0G2UK59/275-423 Microviridae Fen7895_21
A0A0G2UMG1/221-368 Microviridae Fen685_11
A0A0G2UK08/293-440 Microviridae Bog5275_51
A0A0C5AFY2/275-422 Eel River basin pequenovirus
A0A0C5AG40/214-371 Eel River basin pequenovirus
A0A0G2UG13/290-436 Microviridae Bog9017_22
W1I5M6/282-430 uncultured bacterium
A0A078SZM9/295-443 Parabacteroides distasonis
A0A0G2UEI2/308-455 Parabacteroides phage YZ-2015b
A0A0C5AFV8/260-407 Eel River basin pequenovirus
M9M206/43-191 uncultured marine virus
X5K0Y8/285-433 Elizabethkingia anophelis
A0A0G2UK63/261-410 Microviridae Fen7918_21
A0A0G2UMC2/257-409 Microviridae Bog1249_12
A0A0G2UEE2/279-429 Microviridae Fen4707_41
0.1
Tree balance = 0.0

Supplementary Figure S5. PHACTS [2] prediction on the bacteriophage HFM predicted ORFs. PHACTS uses a machine learning approach based on protein sequences to classify if a phage is more likely to be temperate or lytic and whether it has a gram-positive or gram-negative host preference. The results do not yield information about the lifestyle of the phage, but they suggest that the phage is likely to invade gram-negative hosts

✔ Lifestyle:

Analysis Statistics

Probability that phage is **Temperate**

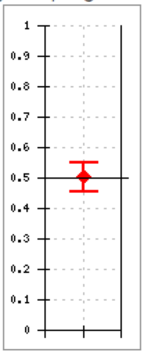

Ten iterations of PHACTS were performed using the default settings. The phage was **non-confidently** predicted as having a **Temperate lifestyle**.

Predicted Class	Averaged Probability	Standard Deviation
Temperate	0.503	0.048

✔ Gram-stain of host:

Analysis Statistics

Probability that phage is **Negative**

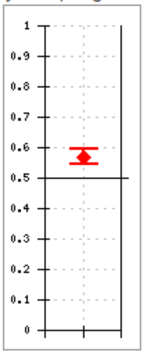

Ten iterations of PHACTS were performed using the default settings. The phage was **confidently** predicted as infecting a **Gram Negative** host.

Predicted Class	Averaged Probability	Standard Deviation
Negative	0.57	0.025

Supplementary Figure S6. Results from two separate Bacteriophage HFM PCRs from the the same set of samples. The main image shows one complete gel. The two smaller gel images show the sizes of the bands in the two size markers used in the main gel, see below for description. We have chosen an image where all bands are clearly distinguishable, despite variations in intensities. The PCR amplifications are of bacteriophage HFM in individual FESC D samples. Bands of the expected size were only observed in sample F32, where DNA fragments of 267 bp and 155 bp were obtained in PCR 1 and 2, respectively. Ntc, L50 and L100 refer to non-template control, 100 bp DNA Ladder (ThermoFisher Scientific, cat.no. 15628050) and 50 bp DNA Ladder (ThermoFisher Scientific, cat.no. 10416014).

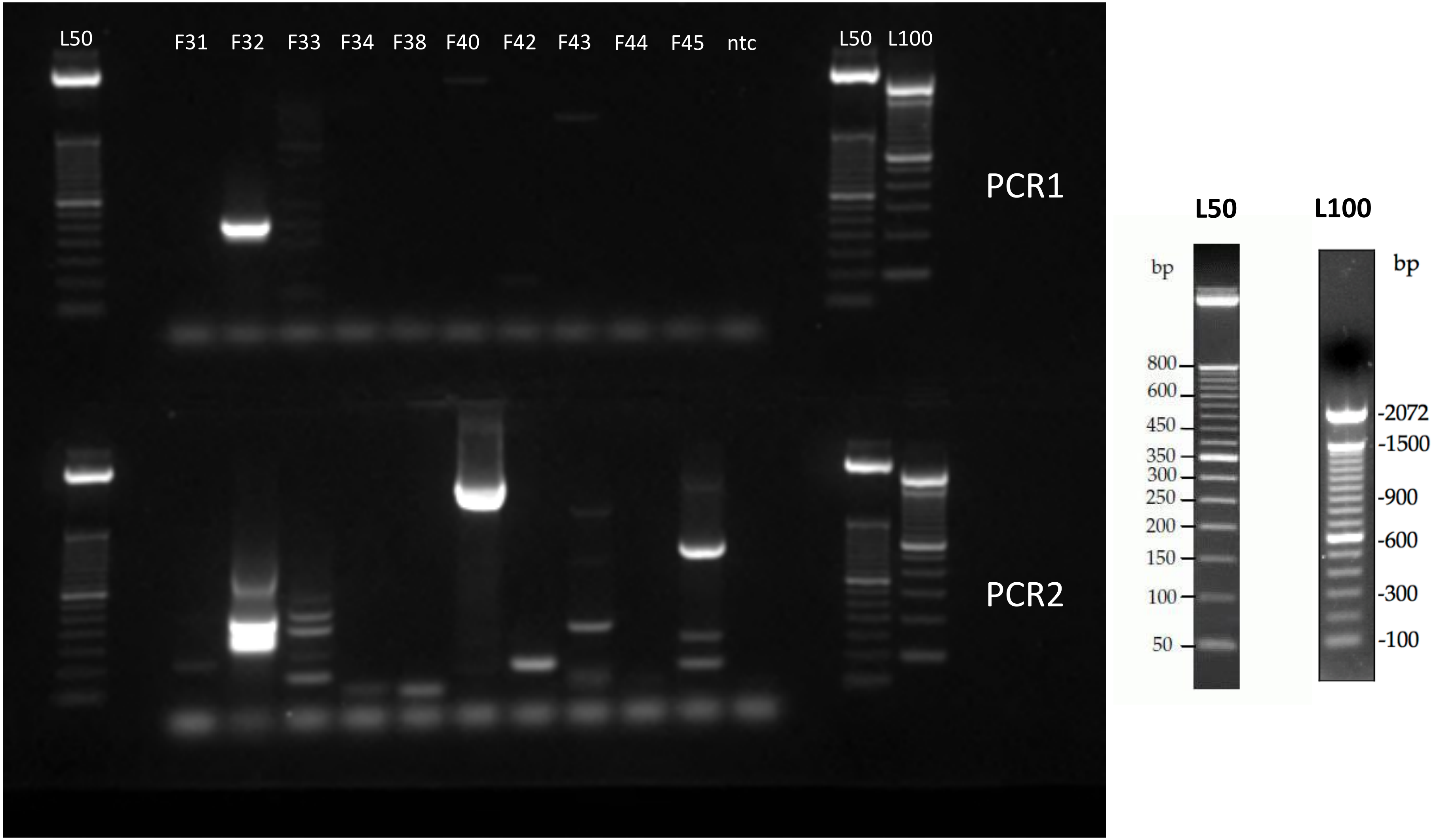
L50
F31
F32
F33
F34
F38
F40
F42
F43
F44
F45
ntc
L50
L100
PCR1
PCR2
L50
L100
bp
800
600
450
350
300
250
200
150
100
50
bp
-2072
-1500
-900
-600
-300
-100

Supplementary Table S1 - Description of viral metagenomics libraries

library_id	sample_type	nucleic_acid	platform	reads	avg_read_len
**ER8QEOW01**	nasopharyngeal swabs	DNA	GS	160,120	99.6
**ER8QEOW02**	nasopharyngeal swabs	RNA	GS	138,941	102.8
**FC8LRL301**	nasopharyngeal swabs	DNA	GS FLX	165,715	185.9
**FC8LRL302**	nasopharyngeal swabs	RNA	GS FLX	239,014	208.1
**FCPU0RF01**	serum	DNA	GS FLX	177,184	143.7
**FCPU0RF02**	serum	RNA	GS FLX	113,542	140.5
**FPFNBIF01**	serum	DNA	GS FLX	25,767	170.1
**FPFNBIF02**	serum	RNA	GS FLX	305,191	199.1
**FSTRRC101**	serum	DNA	GS FLX	28,217	177.9
**FS22EC101**	serum	DNA	Titanium	256,310	193.2
**FS22EC102**	serum	RNA	Titanium	189,704	187.6
**FTSPZO101**	cerebrospinal fluid	DNA	Titanium	158,065	220.1
**FTSPZO102**	cerebrospinal fluid	RNA	Titanium	51,683	134.5
**GB7HT0Z01**	nasopharyngeal & throat swabs	DNA	Titanium	199,388	258.8
**GB7HT0Z02**	nasopharyngeal & throat swabs	RNA	Titanium	233,531	264.4
**GB3LKKR01**	feces	DNA	Titanium	723,577	327.0
**GB3LKKR02**	feces	RNA	Titanium	736,239	323.7
			**OVERALL**	3,902,188	

Supplementary Table S2 - Summary of predicted families hits to different databases

Cluster	sample_origin	RNAcode_pvalue	composite score	hits_to
1217	feces	7.70E-10	7.57	env_nt;metahit_2014_cds;metahit_2014_pep;nr;env_nr
339b	feces	1.07E-06	7.52	metahit_2014_cds;env_nt;metahit_2014_pep
211b	feces	3.36E-14	7.49	env_nt;metahit_2014_cds;metahit_2014_pep
562	CSF;serum	4.59E-07	7.39	
297a	feces	0.001	7.38	metahit_2014_cds;env_nt;metahit_2014_pep;
457	CSF;serum	1.35E-08	7.32	env_nt
348	feces	1.42E-08	7.32	env_nt;metahit_2014_cds;metahit_2014_pep;
502	feces	3.60E-07	7.3	metahit_2014_cds;env_nt;metahit_2014_pep;
540	CSF;mucus;serum	3.76E-04	7.24	env_nt
241b	feces	5.54E-06	7.24	env_nt;metahit_2014_cds;metahit_2014_pep;
179a	feces	0.005	7.23	metahit_2014_cds;env_nt;metahit_2014_pep;
406b	CSF;feces	0.001	7.21	env_nt;metahit_2014_cds;metahit_2014_pep
1073	feces	1.66E-10	7.19	metahit_2014_cds;env_nt;metahit_2014_pep
211a	feces	3.90E-06	7.17	metahit_2014_cds;env_nt;metahit_2014_pep;
589	feces	8.36E-04	7.12	metahit_2014_cds;env_nt;metahit_2014_pep;
375a	feces	0.013	7.09	metahit_2014_cds;env_nt;metahit_2014_pep;
182a	feces	5.23E-04	7.05	env_nt;metahit_2014_cds;metahit_2014_pep;
1423	CSF;serum	1.00E-04	7.01	
258	feces	0.015	6.99	env_nt;metahit_2014_cds;metahit_2014_pep;
297b	feces	0.006	6.99	metahit_2014_cds;env_nt;metahit_2014_pep;
1057	CSF;serum	3.44E-06	6.99	
241a	feces	0.002	6.94	env_nt;metahit_2014_cds;metahit_2014_pep;
956b	serum	0.003	6.94	nr
321b	feces	1.88E-07	6.93	metahit_2014_cds;env_nt;metahit_2014_pep;
182b	feces	1.54E-04	6.9	metahit_2014_cds;env_nt;metahit_2014_pep;
532	feces	0.001	6.79	metahit_2014_cds;env_nt;metahit_2014_pep;nr
787b	feces	0.016	6.78	env_nt;metahit_2014_pep;

565b	CSF;serum	0.003	6.74	nr
1085	CSF;mucus;serum	0.006	6.73	metahit_2014_pep
612a	feces	0.004	6.56	metahit_2014_cds;env_nt;metahit_2014_pep;
179b	feces	0.01	6.54	metahit_2014_cds;env_nt;
113b	feces	0.008	6.32	metahit_2014_cds;env_nt;metahit_2014_pep;

Supplementary Table S3 - Summary of hits of protein families to MetaHIT database. "n" stands for the CDS version, "p"stands for the protein version of the database

Cluster	composite score	sample_origin	MetaHIT
1217	7.57	feces	2014_n;2014_p
339b	7.52	feces	2014_n;2014_p
211b	7.49	feces	2014_n;2014_p
562	7.39	CSF;serum	
297a	7.38	feces	2014_n;2014_p
457	7.32	CSF;serum	
348	7.32	feces	2014_n;2014_p
502	7.3	feces	2014_n;2014_p
241b	7.24	feces	2014_n;2014_p
540	7.24	CSF;mucus;serum	
179a	7.23	feces	2014_n;2014_p
406b	7.21	CSF;feces	2014_n;2014_p
1073	7.19	feces	2014_n;2014_p
211a	7.17	feces	2014_n;2014_p
589	7.12	feces	2014_n;2014_p
375a	7.09	feces	2014_n;2014_p
182a	7.05	feces	2014_n;2014_p
1423	7.01	CSF;serum	
1057	6.99	CSF;serum	
297b	6.99	feces	2014_n;2014_p
258	6.99	feces	2014_n;2014_p
241a	6.94	feces	2014_n;2014_p
956b	6.94	serum	
321b	6.93	feces	2014_n;2014_p
182b	6.9	feces	2014_n;2014_p

532	6.79	feces	2014_n;2014_p
787b	6.78	feces	2014_p
565b	6.74	CSF;serum	
1085	6.73	CSF;mucus;serum	2014_p
612a	6.56	feces	2014_n;2014_p
179b	6.54	feces	2014_n
113b	6.32	feces	2014_n;2014_p

Supplementary Table S4 - Summary of hits of protein families to the NCBI environmental databases. (n) stands for env_nt,(p) for env_nr

Cluster	composite score	sample_origin	env_hits
1217	7.57	feces	marine sediment (p);gut (n)
339b	7.52	feces	gut (n)
211b	7.49	feces	gut (n)
562	7.39	CSF;serum	
297a	7.38	feces	gut (n)
457	7.32	CSF;serum	wastewater (n)
348	7.32	feces	gut (n)
502	7.3	feces	Human gut (n)
241b	7.24	feces	gut (n)
540	7.24	CSF;mucus;serum	wastewater (n)
179a	7.23	feces	gut (n)
406b	7.21	CSF;feces	gut (n)
1073	7.19	feces	gut (n)
211a	7.17	feces	gut (n)
589	7.12	feces	gut (n)
375a	7.09	feces	gut (n)
182a	7.05	feces	Human gut (n)
1423	7.01	CSF;serum	
1057	6.99	CSF;serum	
297b	6.99	feces	gut (n)
258	6.99	feces	gut (n)
241a	6.94	feces	gut (n)
956b	6.94	serum	
321b	6.93	feces	gut (n)
182b	6.9	feces	Human gut (n)

532	6.79	feces	Human gut (n)
787b	6.78	feces	Human gut (n)
565b	6.74	CSF;serum	
1085	6.73	CSF;mucus;serum	
612a	6.56	feces	gut (n)
179b	6.54	feces	gut (n)
113b	6.32	feces	Gut (n)

Supplementary methods

Protein family construction

We employed RNAcode's evolutionary model to distinguish between coding and non-coding sequences. However, because RNAcode depends on long, high-quality multiple alignments for its predictions, it has previously been impossible to use this method for gene prediction from short read metagenomics datasets. We have addressed this issue by adding several filtering steps. In short, we generated candidate protein families by building high-quality multiple sequence alignments from clusters of similar sequences. Subsequently, we calibrated the RNAcode thresholds to detect coding sequences in short reads, and combined these with sequence-based measures like ORF length and sequence complexity and to select high-confidence candidate protein families. These steps are outlined in Figure 1.

Clustering

To build the multiple sequence alignments, we began by clustering sequences by similarity with the MCL Algorithm[3]. This procedure yielded a total of 248,813 raw clusters, of which 13,861 had between 3 and 250 sequences (Supplementary Figure 1a) and were selected for building multiple sequence alignments. However, closer examination of these clusters revealed that many of them contained sequences with very high identity. Considering that RNAcode has been reported to perform poorly on alignments with low sequence diversity[4], we opted to sub-cluster sequences with more than 95% identity within each cluster, using a single-linkage approach, and replaced them with a single representative copy. We hypothesized that these highly similar sequences had likely originated from the same source, and thus could confound RNAcode, which would consider them two independent proteins. Supplementary Figure 1b shows the cluster size distributions before and after sub-clustering. The increase in the number of small clusters, and specifically the large fraction (64%) of clusters were reduced to one or two sequences after sub-clustering, strengthens our hypothesis that most initial clusters included nearly identical sequences, thus validating our strategy. It is possible that some of the singletons are true ORFans, but this cannot be determined without additional homologous sequences.

Multiple sequence alignment of clusters

It is well known that multiple sequence alignment quality has a large effect on bioinformatics tools that use them as input[5]. RNAcode is no exception, and unfortunately, viral-enriched metagenomics data is close to a worst-case scenario for a multiple sequence alignment. Factors such as variable coverage, population variability, and sequence quality of next-generation sequencing (NGS) data could lead to clusters composed of sequences of different lengths, which in turn will result in alignments with a large number of gaps. Under these circumstances, RNAcode could erroneously predict mostly-gapped regions to have high coding potential, due to the low effective number of triplets that can be scored (S. Washietl, personal communication). In order to overcome this problem, we used a codon-aware aligner, MACSE, which takes possible frameshifts and stop codons into account[6], coupled with manual inspection and removal of low-coverage regions and poorly-aligning sequences. In the absence of an accurate automated solution, we found this to be the most effective way to ensure that RNAcode would not erroneously predict coding potential due to poor alignment quality. To ensure high quality, we here focus on clusters of more than 8

sequences. 456 clusters were aligned, of which 209 low quality alignments (e.g. poor overlap) were discarded.

Selection of high-confidence protein families

As RNAcode was originally designed to work with longer alignments, we carried out a calibration procedure using simulated reads of varying lengths from coding and non-coding regions to optimize it for shorter alignments. The results of this calibration procedure suggest that the tool can accurately separate true ORFs from non-coding regions for alignment lengths as short as 50 nt, using a p-value threshold of 0.15 (Supplementary Figure 1c). Based on these findings, we applied the $P \leq 0.15$ threshold to the remaining clusters, resulting in the exclusion of 154 families predicted to not have significant coding potential.

RNAcode does not take sequence complexity into account when assessing coding potential, and thus may yield low p-values for short tandem amino-acid repeats. Since such repeats often appear in non-coding regions, we applied the `seg` algorithm[7] to identify and exclude low-complexity candidates, complementing RNAcode's predictions. In total, an additional 61 clusters were excluded based on low complexity.

Finally, to rank our resulting families in terms of coding potential, we calculated a composite score that combines the RNAcode score, sequence complexity, and ORF length. Although *ad hoc*, in our experience the composite score is a convenient heuristic that was more useful than simply evaluating the p-value alone, since it integrates external, biologically relevant information that RNAcode does not consider.

From the original set of 456 clusters selected for further analysis, our approach identified 32 high-confidence candidate novel ORFan protein families, which we ranked by our ad-hoc composite score. Their final trimmed and filtered alignments were composed of a median of 6 sequences and have a predicted ORF length between 30 and 130 amino acids (Figures 3a and 3b). The coding potential was assessed using RNAcode as described in the previous sections, and the RNAcode p-value distribution for the 32 clusters ranged from very confident predictions with p-values around 10^{-10} to values close to 0.02 (Figure 2a). The RNAcode output for cluster 457 is shown in Figure 3d, and RNAcode predictions for all 32 families are available in Supplementary Fig. S2.

Phylogenetic tree construction

The ORF6 protein sequence and all 1657 sequences in the Pfam full alignment of Phage_F (PF02305) were aligned to the Pfam HMM with hmmalign. Unalignable regions in the N- and C-terminus were removed, keeping a conserved core region of about 150 residues. Sequences with >50% gap residues were removed, and sequences >90% identical to other sequences were removed, leaving 331 representative and non-redundant sequences. The tree was built with the Neighbor Joining method using Scoredist distance correction using Belvu[8], with 1000 bootstraps

References

1. Darling, A. C. E., Mau, B., Blattner, F. R. & Perna, N. T. Mauve: multiple alignment of conserved genomic sequence with rearrangements. *Genome Res.* **14,** 1394–403 (2004).
2. McNair, K., Bailey, B. A. & Edwards, R. A. PHACTS, a computational approach to classifying the lifestyle of phages. *Bioinformatics* **28,** 614–618 (2012).
3. Enright, A. J., Van Dongen, S. & Ouzounis, C. A. An efficient algorithm for large-scale detection of protein families. *Nucleic Acids Res.* **30,** 1575–1584 (2002).
4. Washietl, S. *et al.* RNAcode: robust discrimination of coding and noncoding regions in comparative sequence data. *RNA* **17,** 578–94 (2011).
5. Blackburne, B. P. & Whelan, S. Measuring the distance between multiple sequence alignments. *Bioinformatics* **28,** 495–502 (2012).
6. Ranwez, V., Harispe, S., Delsuc, F. & Douzery, E. J. P. MACSE: Multiple alignment of coding SEquences accounting for frameshifts and stop codons. *PLoS One* **6,** (2011).
7. Wootton, J. C. & Federhen, S. Analysis of compositionally biased regions in sequence databases. *Methods Enzymol.* **266,** 554–571 (1996).
8. Sonnhammer, E. L. & Hollich, V. Scoredist: a simple and robust protein sequence distance estimator. *BMC Bioinformatics* **6,** 108 (2005).
